# 2D and 3D anticancer properties of C2-functionalised glucosamine-Pt (IV) prodrugs based on cisplatin scaffold

**DOI:** 10.3389/fchem.2024.1388332

**Published:** 2024-05-06

**Authors:** Eoin Moynihan, Maria Galiana-Cameo, Monica Sandri, Andrea Ruffini, Silvia Panseri, Trinidad Velasco-Torrijos, Monica Montesi, Diego Montagner

**Affiliations:** ^1^ Department of Chemistry, Maynooth University, Maynooth, Ireland; ^2^ Institute of Science, Technology and Sustainability for Ceramics (ISSMC)– National Research Council (CNR), Faenza, Italy; ^3^ Kathleen Londsdale for Human Health Research, Maynooth University, Maynooth, Ireland

**Keywords:** target therapy, cisplatin, glycoconjugate, glucosamine, anticancer properties

## Abstract

A series of C2-functionalied Pt (IV) glycoconjugates based on glucosamine have been synthesised, characterised and tested as anticancer agents on a series of different 2D and 3D cancer cell lines. The carbohydrate will act as a targeted delivery system to improve the selectivity, exploiting the Warburg Effect and the GLUTs receptors that are overexpressed in most of the cancer cells. The hydroxyl at C2 of the carbohydrates does not participate in hydrogen bonding with the GLUTs receptors, making C2 an attractive position for drug conjugation as seen in literature. In this study, we use the amino functionality at the C2 position in glucosamine and Copper-catalysed Azide-Alkyne Cycloaddition “click” (CuAAC) reaction to connect the prodrug Pt (IV) scaffold to the carbohydrate. We have investigated complexes with different linker lengths, as well as acetyl protected and free derivatives. To the best of our knowledge, this study represents the first series of Pt (IV) glucosamine-conjugates functionalised at C2.

## Introduction

**FIGURE 1:**
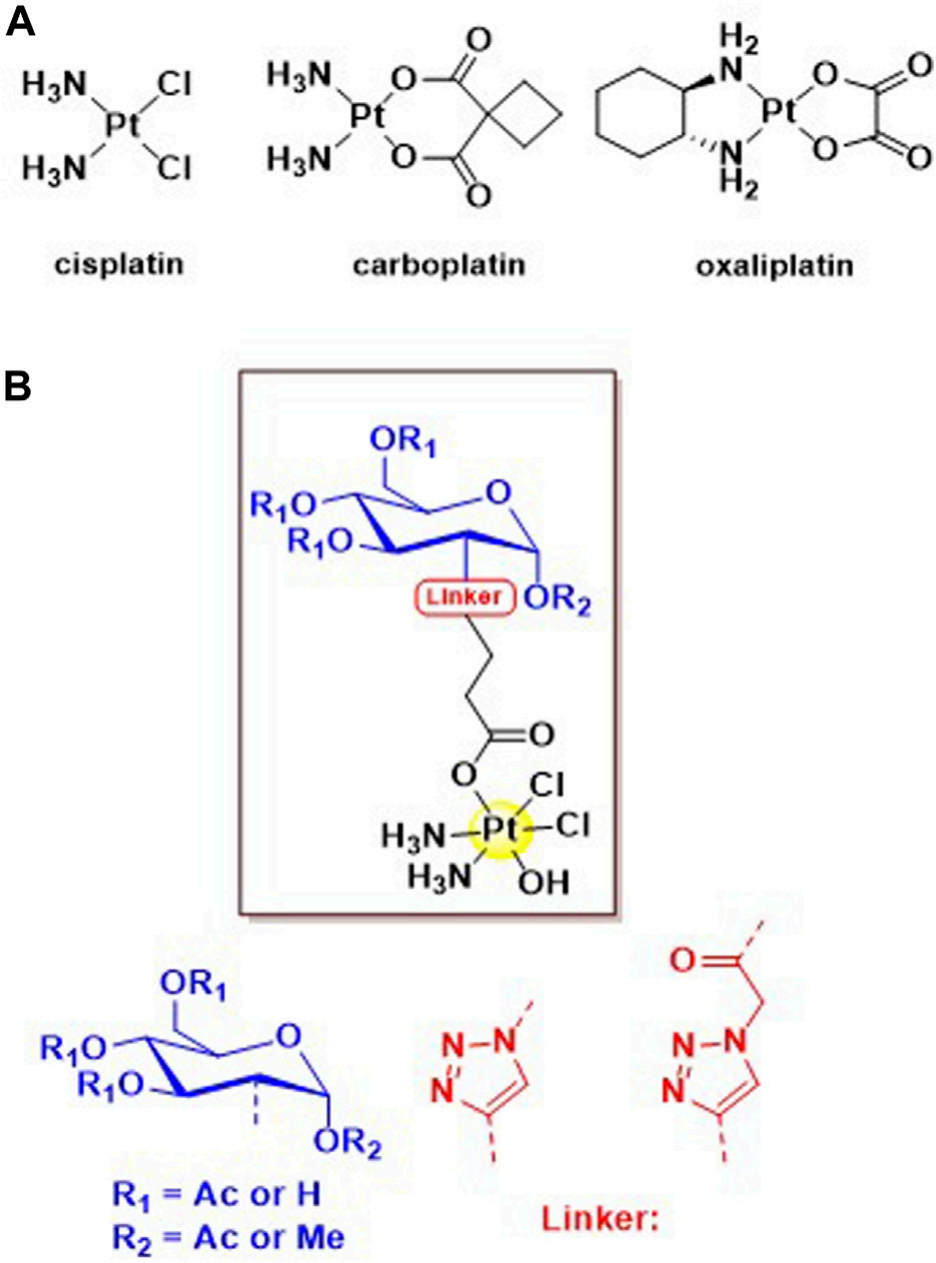
**(A)** Structures of the three globally approved Pt (II) chemotherapeutics, cisplatin, carboplatin and oxaliplatin. **(B)** General structure of the four novel C2 glycoconjugated Pt (IV) pro-drugs based on the cisplatin scaffold.

The most well-known anticancer metal-based drug is cisplatin approved by FDA in 1978, followed shortly by the approval of “second generation Pt (II) chemotherapeutics” carboplatin (Ho et al., 2016) and oxaliplatin (Alcindor and Beauger, 2011) (Figure 1A) in 1989 and 2002 respectively. One of the major drawbacks in the use of cisplatin is that it lacks specificity for cancer cells and causes damage to healthy cells; specific side effects of cisplatin include nephrotoxicity (Miller et al., 2010), ototoxicity (Callejo et al., 2015), neurotoxicity (Kanat et al., 2017), cardiotoxicity (Hu et al., 2018) and hepatotoxicity (Taghizadeh et al., 2021). Some of these side effects can be overcome by the use of second generation chemotherapeutics, however, more recently, Pt (IV) prodrugs have shown great potential in overcoming cisplatin’s drawbacks (Brandon and Dabrowiak, 1984). Pt (IV) complexes are considered prodrugs because they must be reduced intracellularly to the counterparts Pt (II) to carry out their anticancer activity (Beirne et al., 2023).

This study aims to use carbohydrate targeting vectors to circumvent the drawbacks of Pt (II)-based chemotherapeutics and to improve selectivity for cancer cells compared to non-cancerous tissues. Carbohydrates are metabolised differently in cancer cells compared to healthy cells by deviating from the typical cycle of ATP production. Cells switch from oxidative phosphorylation (OXPHOS) to glycolysis for the synthesis of ATP, reducing ATP production efficiency (Ma et al., 2017b). This lowered energy production results in the overexpression of glucose transporter (GLUT) proteins on the cell surface, which can be used to selectively target cancer cells. This observation was first noted by Otto Warburg in the 1920s and was coined “The Warburg Effect” (Warburg et al., 1927). This is indeed a viable method of tumour targeting, which can be seen in the work from the group of Wang et al. (2016), Ma et al. (2016), Ma et al. (2017a), Ma et al. (2018) who were among the first to synthesize Pt (IV) glycoconjugates.

Previously, our group has synthesized libraries of acetylated and free glycoconjugated Pt (IV) prodrugs where the carbohydrate moieties were functionalized through Copper-catalysed Azide-Alkyne Cycloaddition “click” (CuAAC) chemistry on the anomeric C1 carbon to target GLUTs on osteosarcoma cell lines (Moynihan et al., 2021; Moynihan et al., 2023). However, it is recognised that C1 is not an optimal site of functionalization for the transport of glucose through GLUTs. GLUTs 1-4 are among the most meticulously characterized, with GLUT1 being one of the first characterized and crystallized (Deng et al., 2014). Based on the data provided by the crystal structure of GLUT1, the optimal position to functionalize glucose was determined to be C2. The hydroxyl groups at C1, C3, C4, C6 and the ring oxygen are all important for hexose binding within the protein binding pocket. C4, when switched to the axial position in galactose led to a strong decrease in binding affinity in comparison to when the hydroxyl is in the equatorial position as in glucose (Barnett et al., 1973; Holman, 2020). The hydrogen bond donor and acceptor ability of each of these hydroxyl groups is also important for substrate recognition. Hydrogen bond acceptor positions being C1, C3 and C6, while C4 is the only hydrogen bond donor. The hydroxyl at C2 however, does not participate in hydrogen bonding, proven by the fact that 2-deoxyglucose is transported by all class 1 GLUTs, making C2 an attractive position for drug conjugation (Holman, 2020). This was further proven by Patra et al. (2016) who determined that functionalization in the C2 position of their Pt (II) glycoconjugates both improved intracellular platinum accumulation as well as the cytotoxicity of the complex.

Interestingly, the C2 position has long been used to improve the uptake of imaging and sensing agents, but to our knowledge, it is scarcely taken advantage of in anticancer agents. In particular, 2-[^18^F]-fluoro-2-deoxy-D-glucose (FDG) is used for the detection and staging of cancer through positron emission tomography (PET) (Carbó and Rodríguez, 2023). FDG is transported into the cell in the same manner as glucose, however, In the cytosol, it is phosphorylated into fluorodeoxyglucose 6-phosphate by hexokinase enzymes. Once in this form, further glycolysis is prevented as it cannot be converted to deoxyfructose 6-phosphate, leaving fluorodeoxyglucose 6-phosphate trapped inside the cell (Pauwels et al., 2000). This accumulation can then serve as a contrast agent for imaging by detection of high energy gamma rays emitted from the decaying ^18^F atoms (Sung et al., 2020). The C2 position has also been used to functionalise fluorescent probes as sensing agents, which is expertly reviewed by Kim et al. (2012).

To this end, our group successfully synthesized and completely characterized a series of C2 modified Pt (IV) glycoconjugates based on glucosamine (Figure 1B); the reduction properties and the physiological stability through treatment with ascorbic acid is also assessed. We took advantage of the amino functionality already present in this monossacharide, to avoid lenghty synthetic strategies aimed at the selective functionalization of the C2 position in glucose. (Wang and Demchenko, 2019). To the best of our knowledge, this is the first time that Pt (IV) glucosamine-conjugates functionalised at C2 are reported in literature.

In this study, we aim to comprehensively evaluate the biological activity of this series of C2-functionalised glucosamine-conjugates Pt (IV) prodrugs, shedding light on their cytotoxicity profiles, and potential for targeting different cancer cells. As already demonstrated, the presence of the glycoconjugates enhanced the tumour-targeting capabilities of the drugs and improved pharmacokinetic profiles compared to traditional platinum agents (Moynihan et al., 2021). Through their interaction with the class 1 glucose transporters (GLUTs), which are overexpressed in cancer cells, these glycoconjugates enable targeted therapy, delivering cisplatin specifically to malignant tissues while minimizing off-target effects (Zambrano et al., 2019).

Through the exploration of the proposed complexes in 2D and 3D scaffold-based *in vitro* model, we seek to elucidate the therapeutic potential of these novel compounds and their role in advancing the field of platinum-based chemotherapy.

## Materials and methods

All reagents and reactants were purchased from commercial sources. The two sources used were Sigma-Aldrich and Fluorochem. All solvents were used without further purification. Compounds **9**–**13** were synthesised as previously reported (Nokami et al., 2009; Miller et al., 2015; Arimitsu et al., 2020). Cisplatin and oxoplatin were synthesised as previously reported (Dhara, 1970; Chen et al., 2020).

The elemental analysis studies (carbon, hydrogen, and nitrogen) were performed by means of a PerkinElmer 2400 series II analyzer. HR-Mass Spectra were recorded with a Waters LCT Premier XE Spectrometer. NMR: ^1^H, ^13^C and ^195^Pt NMR spectra were obtained in a solution of D_2_O or DMSO-*d*_*6*_ at 300 K, in 5-mm sample tubes, with a premium shielded Agilent Varian 500 MHz (operating at 500.13, 125.75, and 107.49 MHz, respectively). The ^1^H and ^13^C chemical shift was referenced to the residual impurity of the solvent. The external reference was Na_2_PtCl_4_ in D_2_O (adjusted to δ = −1628 ppm from Na_2_PtCl_6_) for ^195^Pt. The stability was followed using high-performance liquid chromatography (HPLC) with a Phenomenex Luna C18 (5 μM, 100 Å, 250 mm × 4.60 mm i.d.) column at room temperature at a flow rate of 1.0 mL/min with 254 nm UV detection. Mobile phase containing 80:20 acetonitrile (0.1% trifluoroacetic acid): water (0.1% trifluoroacetic acid): the complexes were dissolved in DMF (0.5 mL) and diluted to a final concentration of 0.5 mM using acetonitrile and water solution (1/1) and 2 mM 4-(2-hydroxyethyl)piperazine-1-ethanesulfonic acid (HEPES) buffer (pH 6.8). Infrared (IR) spectra were recorded in the region 4000–400 cm^−1^ on a Perkin Elmer precisely spectrum 100 FT/IR spectrometer. The solid samples were run using ATR.

### Reduction studies

Measurements were obtained in a mixture 1/1 of DMSO-d_6_ and PBS buffer (pH = 6.8) at 300 K, in 5-mm sample tubes, with a premium shielded Agilent Varian 500 MHz (operating at 500.13 MHz). Complex **3** (5 mg, 6.12 µmol) was dissolved in 250 µL of DMSO-*d*_*6*_. Ascorbic acid (10 mg, 10 Equiv.) was dissolved in 250 µL of PBS buffer and added to the platinum complex. ^1^H NMR spectra were recorded every 7 min for 1 h. After no reduction had taken place initially, the ^1^H NMR spectrum was recorded every hour for 4 hours, and finally every 24 h until full reduction of Pt (IV) to Pt (II) and cleavage of the axial ligand was observed after 72 h. Complex **4** (5 mg, 7.19 µmol) was dissolved in 250 µL of DMSO-*d*_*6*_. Ascorbic acid (12 mg, 10 Equiv.) was dissolved in 250 µL of PBS buffer and added to the solution containing the complex. The^1^H NMR spectra were recorded every 7 min for 1 h. After no reduction had taken place initially, the ^1^H NMR spectrum was recorded every hour for 2 hours, and finally every 24 h until full reduction of Pt (IV) to Pt (II) and cleavage of the axial ligand was observed after 24 h.

### *In vitro* biological evaluation

*In vitro* screening of the four complexes (1–4) were performed to evaluate their biological activity towards different phenotypes of tumour cells: three osteosarcoma cell lines (MG63, U2-OS and SAOS-2) and one Human Adenocarcinoma cell lines (MDA-MB-468) and one Human Glioblastoma cell lines (U87), compared to cisplatin. The drugs were reconstituted in Dimethyl Sulfoxide (DMSO) at 1 mg/mL concentration before being diluted in culture media at the required concentration. A 2D *in vitro* screening of all the drugs was performed at 72 h at different concentrations (15, 30, and 60 µM) on all the different cell lines in terms of cell viability.

Based on the outcome of the screening, the anticancer activity of complexes 3 and 4 supplied at 30 µM was evaluated in a more predictive 3D *in vitro* scaffold-based model of osteosarcoma. For the model, a composite hydroxyapatite-based scaffold (MgHA/Coll) as a bone-like matrix was used in combination with MG63 cells and after 72 h of culture with the complexes, cellular uptake, cell viability and morphology were evaluated. For both 2D and 3D *in vitro* cell cultures, cisplatin was used as a control group and cells only were used as a negative control.

### Cell culture

Human Osteosarcoma cell lines MG63 (ATCC^®^ CRL1427™), SAOS-2 (ATCC^®^ HTB-85™), U2-OS (ATCC^®^ HTB-96™); Human Adenocarcinoma cell lines isolated from breast cancers MDA-MB-468 (ATCC^®^ HTB231™); and Human Glioblastoma cell lines U87 (ATCC^®^ HTB14™) were purchased from American Type Culture Collection (ATCC) and used for this study.

MG63 cell line was cultured in DMEM F12-GlutaMAX™ Modified Medium (Gibco) supplemented with 10% Foetal Bovine Serum (FBS) (Gibco) and 1% of penicillin/streptomycin mixture (pen/strep) (100 U/mL–100 μg/mL, Gibco). SAOS-2 cell line was cultured in McCoy’s 5A Modified Medium (Gibco) supplemented with 15% and 10% FBS, respectively, and 1% pen/strep. U2-OS cells were cultured using McCoy’s 5 A (modified) medium supplemented with 10% FBS and 1% Pen/Strep. MDA MB 468 cells were cultured in growth media using RPMI 1640 (Gibco), 10% FBS and 1% Pen/Strep; and U87 cells were grown in a complete medium composed of MEM-nucleosides no-ascorbic-acid medium (Gibco), 10% FBS and 1% Pen/Strep.

Cells were kept in an incubator at 37°C under controlled humidity and 5% CO_2_ atmosphere conditions. Cells were detached from culture flasks by trypsinization and centrifuged. The cell number and viability were determined by Trypan Blue Dye Exclusion test and all cell handling procedures were performed under laminar flow hood in sterility conditions.

### Synthesis of bone mimetic scaffolds

The Mg-doped hydroxyapatite collagen composite scaffolds were designed and synthesized at ISSMC of CNR of Italy (Krishnakumar et al., 2018). Briefly, an acid aqueous suspension was prepared by dispersing 150 g of type I collagen gel (Typeone Biomaterials S.r.l., Calimera, LE, Italy) was diluted into a phosphoric acid solution (2.4 g in 500 mL; H_3_PO_4_, 85 wt%, Sigma) at room temperature to obtain an acidic aqueous homogenous suspension. Separately, a basic aqueous suspension was obtained by mixing 2.7 g of calcium hydroxide (Ca(OH)_2_, 95 wt%, Sigma) and 0.35 g of magnesium chloride (MgCl_2_·6H_2_O, 99 wt%, Sigma) in 500 mL of milli-Q water at room temperature to obtain a basic aqueous homogenous suspension. The acidic suspension was dripped into the basic one at 25°C ± 2°C under continuous stirring conditions and maturated for 2 h. Later, the slurry solution was rinsed thrice in milli-Q water and filtered through a metallic sieve (150 m) to exclude unreacted counter ions. The recovered slurry solution was cross-linked with 2 wt% BDDGE (respect to Collagen) at 25°C ± 2°C for 24 h and at 4°C for other 24 h. Later, the solution was rinsed thrice in milli-Q water to remove any residues and freeze-dried (– 40°C and + 25°C) for 48 h under 0.086 mbar vacuum conditions (LIO 3000 PLT, 5PASCAL, Italy). The obtained scaffolds (6 × 4 mm) named bone mimetic scaffolds were sterilized by 25 kGy γ-ray irradiation before the use.

### 3D scaffold-based osteosarcoma models (3D OS model)

For the development of the *in vitro* 3D scaffold-based osteosarcoma model, bone mimetic scaffolds were used as bone-like matrix in combination with MG63 cells. The scaffolds were conditioned in culture media for 24 h before the cell seeding. MG63 cell line was seeded with a density of 3.0 × 10^4^, for cell viability evaluation and 5.0 × 10^4^, for platin uptake evaluation, per scaffold by dropping the cellular suspension on the material upper surface followed by 30 min pre-adhesion at 37°C before cell media addition. The 3D OS model was grown in the incubator under standard culture medium condition for 72 h to allow cell grown and colonization of the scaffold, then the medium was changed, and the drugs were added. The *in vitro* 3D OS models were cultured in the presence of the drugs for 72 h at 37°C under controlled humidity and 5% CO_2_ atmosphere conditions, the cells grown in 3D in standard condition, without the drugs, were used as control group (3D cells only). All cell handling procedures were performed under a laminar flow hood in sterility conditions.

### MTT cell viability assay

A quantitative analysis of cell viability was carried out by MTT assay, following the manufacturer’s instructions. For the *in vitro* 2D cell cultures, all cell lines were seeded at a density of 5.0 × 10^3^ cells/well in 96 well-plates. For *in vitro* 3D cell cultures, see the “3D scaffold-based models of osteosarcoma” paragraph. The MTT reagent [3-(4,5-dimethylthiazol-2-yl)-2,5-diphenyltetrazolium bromide] (5 mg/mL) was dissolved in Phosphate Saline Buffer 1X (PBS 1X). At 72 h, the cells were incubated with 10% media volume MTT solution for 2 h at 37°C, 5% CO_2_ and controlled humidity conditions. The cell culture media was removed and substituted with DMSO (Merck) dissolving formazan crystals derived from MTT conversion by metabolically active cells. For 3D scaffold-based models of osteosarcoma, each scaffold was transferred into a 2 mL Eppendorf and broken by pestles after DMSO addition. After 15 min of incubation under slight stirring conditions, the absorbance of formazan was red at 570 nm by using a Multiskan FC Microplate Photometer (Thermo Fisher Scientific). The values of absorbance are directly proportional to the number of metabolic active cells in each well. One experiment was carried out and a biological triplicate for each condition was performed. For the 3D tumour models, one biological experiment was performed, and two scaffolds for each condition were used.

### Cell morphology evaluation

For the *in vitro* 3D cell cultures cells were treated as previously described. 3D cell cultures were fixed in 4% buffered Paraformaldehyde (PFA) following the manufacturer’s instructions. The fixed samples were permeabilized in PBS 1X with 0.1% (v/v) Triton X-100 (Merck) for 5 min at room temperature and F-actin filaments were highlighted with Alexa Fluor 488 Phalloidin (Invitrogen) for 20 min at room temperature in the dark. DAPI (600 nM) counterstaining was performed for cell nuclei identification, following the manufacturer’s instructions. The images were acquired by using an Inverted Ti-E Fluorescent Microscope.

### Quantification of cellular uptake of Pt

To evaluate the Pt cell internalization in the 3D tumour models, inductively coupled plasma optical emission spectrometry (ICP-OES, Agilent Technologies 5100 ICP-OES, Santa Clara, United States) was used. The 3D model was prepared and treated as mentioned in the previous section. The ICP analysis were performed 24 h after the complexes supply.

Briefly, each sample was washed with 400 µL PBS 1X and stored at −80°C until the analysis. ICP-OES was used for the quantitative determination of Pt ions. Briefly, the samples were dissolved with 500 µL nitric acid (65 wt%) and 2.1 mL of Milli-Q water followed by sonication for 30 min in an ultrasonicated bath. Cells only were prepared similarly. The analytical wavelength of Pt was 265.945 nm. Pt (pg) per scaffold were quantified and a biological triplicate for each condition was performed.

### Statistical analysis

Statistical analysis was performed by using GraphPad Prism Software (8.0.1 version). Cell viability results were represented in the graphs as mean percentage of cell viability respect to cells only (both 2D and 3D model) ± standard deviation. The 2D screening results were analysed by Two-way analysis of variance (Two-way ANOVA) and Tukey’s multiple comparisons test. The Cell viability and Pt Uptake in the 3D model were analysed by One-way analysis of variance (One-way ANOVA) and Tukey’s multiple comparisons test.

## Results and discussion

### Synthesis and characterisation

**SCHEME 1:**
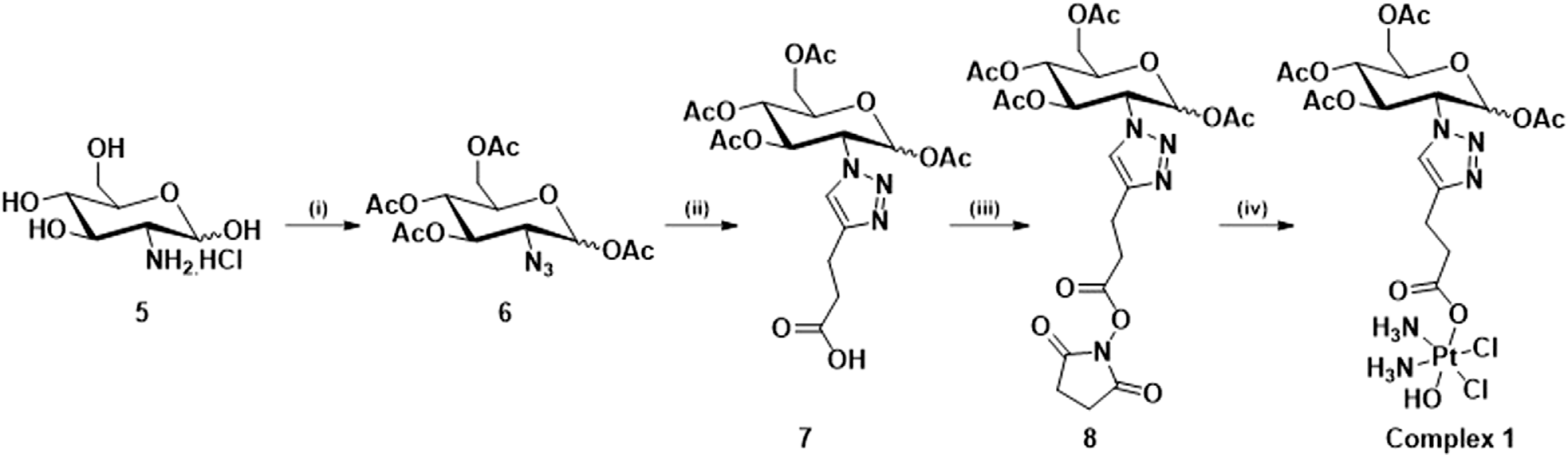
Synthetic route for the synthesis of complex **1**: (i) Tf_2_O, NaN_3_, H_2_O, DCM, K_2_CO_3,_ MeOH, CuSO_4_, Ac_2_O, Pyridine, rt, 16 h, 74%; (ii) 4-pentynoic acid, CuSO_4_, NaAsc, *t*-BuOH, THF, H_2_O, rt, 16 h, 59%; (iii) EDCI, NHS, DCM, rt, 16 h, 89%; (iv) DMSO, 60°C, 16 h, 67%.

**SCHEME 2:**
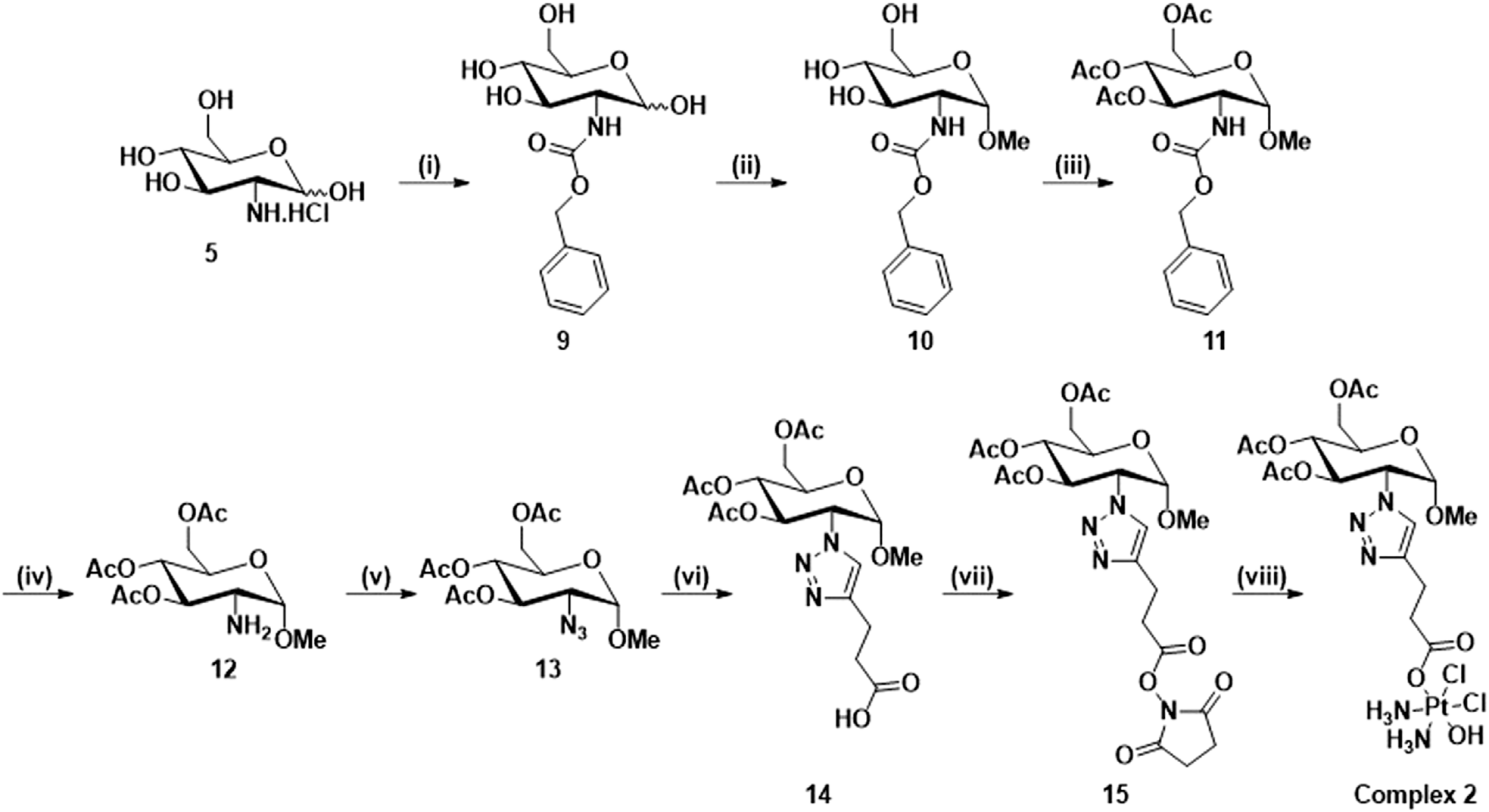
Synthetic route for the synthesis of complex **2**: (i) NaHCO_3,_ Benzyl chloroformate, H_2_O, 16 h, rt, 92%; (ii) HCl/MeOH soln. 1.25 M, 80°C, 18 h; (iii) Pyridine, Ac_2_O, rt, 16 h, 73%; (iv) Pd/C, H_2_, DCM, rt, 16 h; (v) NaN_3_, Tf_2_O, K_2_CO_3_, CuSO_4_.5H_2_O, DCM, MeOH, H_2_O, rt, 16 h, 35%; (vi) 4-pentynoic acid, CuSO_4_.5H_2_O, NaAsc, t-BuOH, H_2_O, rt, 16 h, 65%; (vii) NHS, EDCI, DCM, rt, 16 h, 91%; (viii) Oxoplatin, DMSO, 60°C, 16 h, 86%.

**SCHEME 3:**
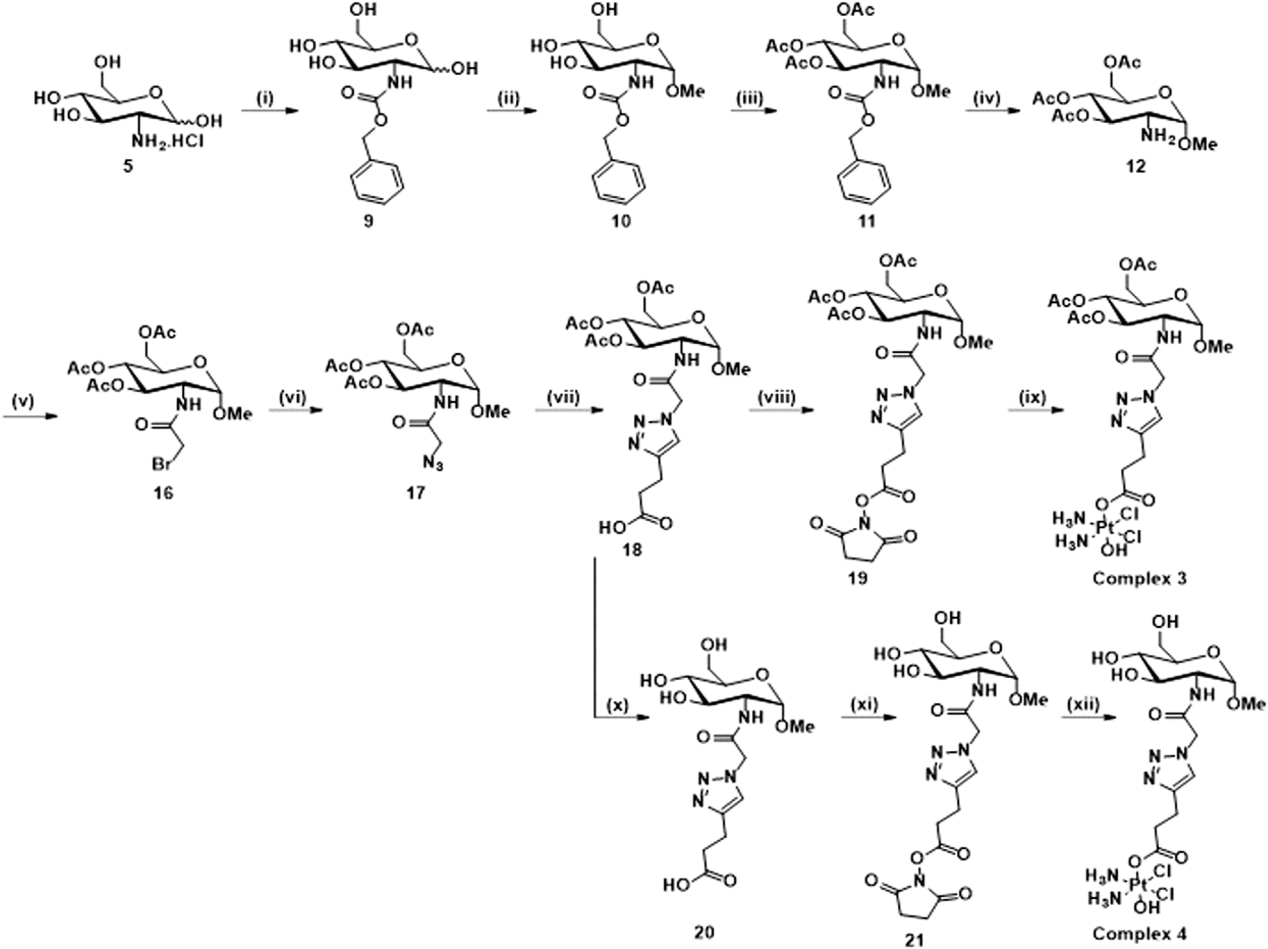
Synthetic route for the synthesis of **3** and **4**: (i) NaHCO_3,_ Benzyl chloroformate, H_2_O, 16 h, rt, 92%; (ii) HCl/MeOH soln. 1.25 M, 80°C, 18 h; (iii) Pyridine, Ac_2_O, rt, 16 h, 73%; (iv) Pd/C, H_2_, DCM, rt, 16 h; (v) Bromoacetyl bromide, NEt_3_, DCM, N_2_, rt, 16 h, 71%; (vi) NaN_3_, DMF, N_2_, 80°C, 16 h, 98%; (vii) 4-pentynoic acid, NaAsc, CuSO_4_.5H_2_O, t-BuOH, H_2_O, rt, 16 h, 41%; (viii) EDCI, NHS, DCM, rt, 16 h, 82%; (ix) Oxoplatin, DMSO, 60°C, 16 h, 82%; (x) NEt_3_, MeOH, H_2_O, 40°C, 16 h, 98%; (xi) TSTU, NEt_3_, DMF, rt, 20 min, 79%; (xii) Oxoplatin, dry DMSO, 40°C, 5 days, 39%.

As described in the introduction, the aim of this work is to synthesize Pt (IV) anticancer prodrugs, based on the cisplatin scaffold which is modified with sugar targeting moieties through conjugation at the C2 position of glucosamine. The sugar moieties are connected to the platinum centre *via* a triazolyl linker exploiting the versatility and mild conditions of the “click” CuAAC reaction, producing four novel complexes, three protected with acetyl protecting groups and one free sugar derivative. The complexes were successfully synthesized as shown in Schemes 1–3, in high yield and purity. (See Supplementary Material for a complete description of the syntheses and spectroscopical characterisation).

Scheme 1 shows the synthetic route to produce complex **1**, which contains a mixture of the α/β-anomers of the glucosamine moiety. The synthesis of compound **6** begins from the commercially available D-glucosamine hydrochloride which undergoes a diazo transfer reaction using the freshly prepared diazo transfer reagent triflyl azide, followed by acetylation with acetic anhydride and pyridine. A CuAAC “click” reaction is then used to conjugate the resulting azide (**6**) to 4-pentynoic acid (ii), the carboxylic acid product (**7**) is then converted to its corresponding active ester (**8**) using EDCI and NHS (iii). Finally, a *trans* esterification reaction is used to obtain complex **1** with a mixture of α- and β-anomers (iv). The final complex **1** was characterised by ^1^H, ^13^C, ^195^Pt-NMR, Elemental Analyses and Mass spectroscopy (See Experimental Section).

To avoid the mixture of α- and β-anomers, complex **2,** which is the *O*-methyl derivative of complex **1**, in which only the α-anomer is present, was synthesized according to Scheme 2. Compounds **9**, **10**, **11**, **12** and **13** were synthesized according to literature procedures (Nokami et al., 2009; Miller et al., 2015; Arimitsu et al., 2020). Briefly, the synthesis begins with the protection of D-glucosamine hydrochloride using benzyl chloroformate **(9)**, which then undergoes Fischer glycosylation with methanol **(10)** followed by the acetylation of the remaining hydroxyl groups, to give the corresponding a-methyl *O*-glycoside after chromatography **(11)**. The C2 amine is reformed using hydrogenative deprotection **(12)** and a diazo transfer with triflyl azide is used to produce **13**. The CUAAC reaction (**14**) and the NHS esterification reactions **(15)** used similar conditions to Scheme 1, however, the click reaction in this scheme and in Scheme 3 required optimisation through the amount of catalyst used and changing the solvent system (See Experimental). The complex **2** was characterised using the same techniques for complexes **1**. Unfortunately, the deprotected derivatives of **1** and **2** could not be synthesized: in the case of complex **1**, the reactivity of the hydroxyl at the anomeric position in the deprotected carboxylic acid (**7**) led to the formation of impurities which we were unable to purify in the final product.

**FIGURE 2:**
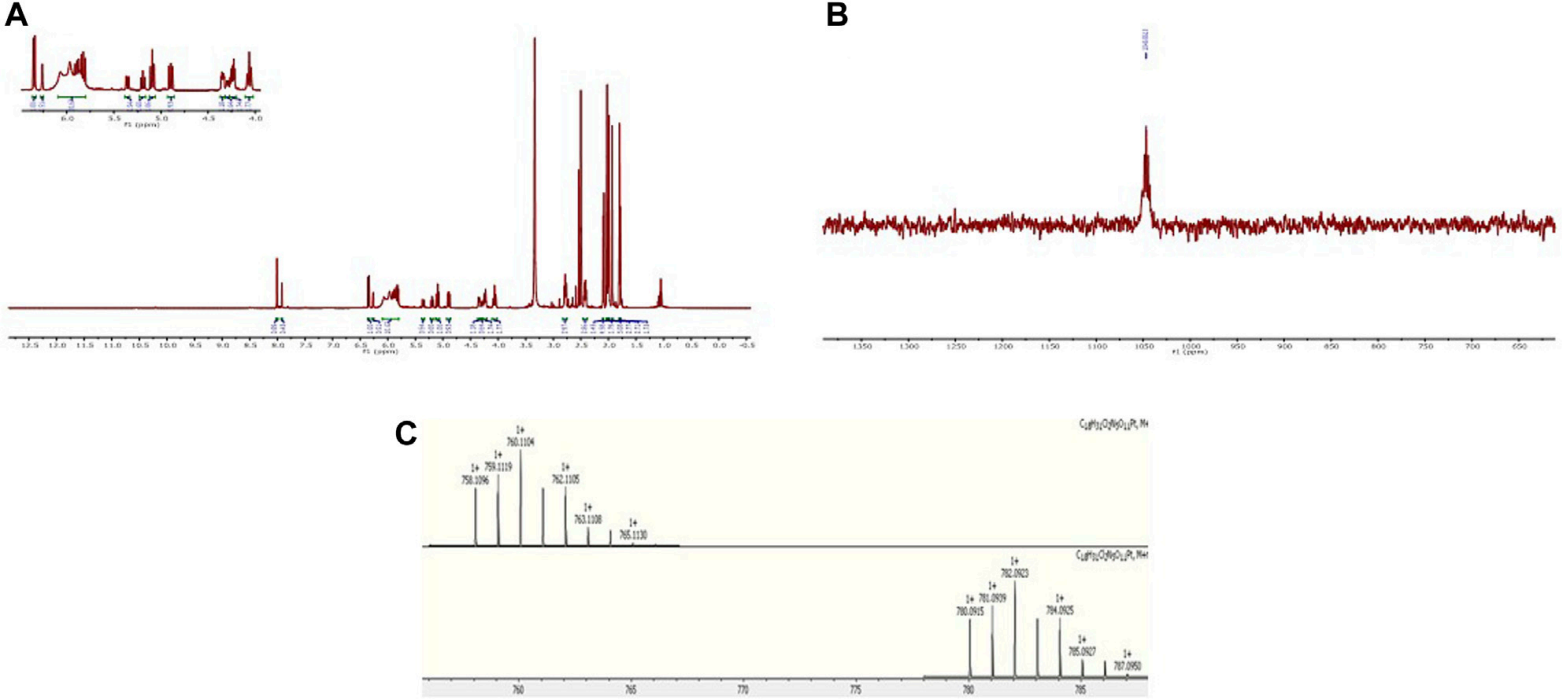
**(A)** ^1^H-NMR of complex **1** in DMSO-d_6_; **(B)** ^195^Pt-NMR of complex **2** in DMSO-d_6_; **(C)** HR-MS spectrum of complex **2**.

Scheme 3 shows the synthesis of complex **3** and its free sugar derivative, complex **4.** For the synthesis of these complexes, the azido functionality (**17**) is not directly connected to the carbohydrate ring as in intermediates (**6, 13**), used in the synthesis of complexes **1** and **2**; instead, a linker featuring an amide rseemblin N-acetyl glucosamine derivatives is used. The free amine (**12**), also intermediate in the synthesis of complex 2, is now converted to an amide through reaction with bromoacetyl bromide 5) and this bromide then undergoes nucleophilic substitution to give the corresponding azide (**17**). Steps (vii), (viii), and (ix) follow the same procedures as for Scheme 2 to yield complex **3**, however in this case it was possible to isolate the free sugar derivative (complex **4**). The synthesis of this complex begins with a deprotection of the carboxylic acid **18** to form compound **20** and the corresponding ester **21** is formed using TSTU (*N*,*N*,*N*′,*N*′-Tetramethyl-*O*-(*N*-succinimidyl)uronium tetrafluoroborate) in just 20 min (xi). This was then added dropwise (under anhydrous conditions) to a suspension of a large excess of oxoplatin at 40°C to yield complex **4** over 5 days (xii). The complexes were characterised using the same techniques as for complexes **1** and **2**. All the complexes show the typical features of Pt (IV) species based on cisplatin scaffold (Supplementary Material). For example, in Figure 2A is reported the ^1^H NMR of complex **1** in DMSO where are clearly visible the NH_3_ protons of the platinum scaffold appearing as a broad triplet at δ 6.00 ppm, that is a typical feature of mono-substituted Pt (IV) complexes. In Figure 2B is reported the ^195^Pt NMR of complex **2** in DMSO which show a peak at δ 1047 ppm, that is also typical of Pt complexes when they are in the oxidation state +4. The HR-MS spectrum of complex **2** is shown in Figure 2C which show a peak m/z (+) 759.1123, 759.1141 corresponding to [M + H]^+^ and another at m/z (+) 781.0964 corresponding to [M + Na]^+^. Both peaks show the typical isotip pattern distribution pf Pt-based complexes.

**FIGURE 3:**
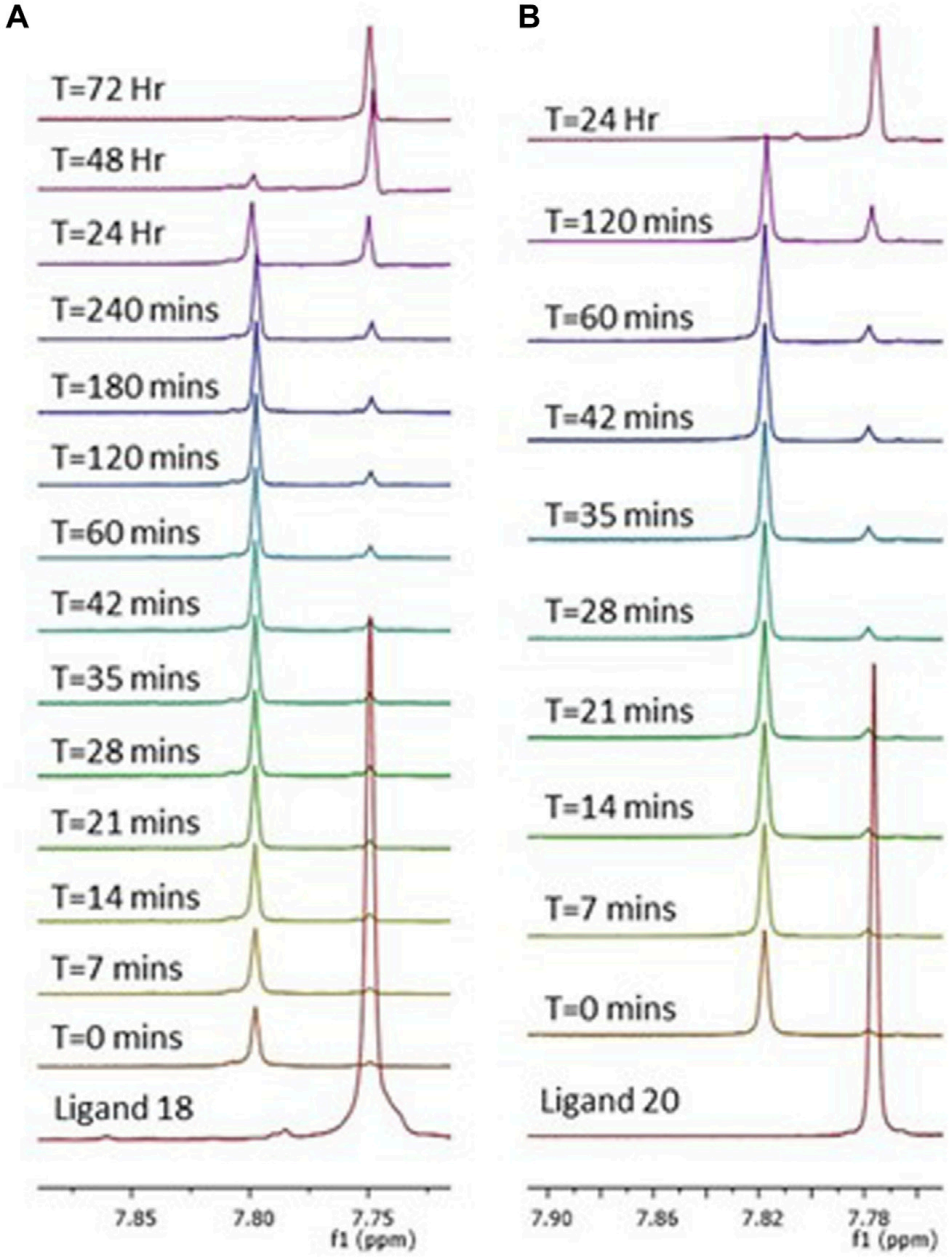
Reduction of complexes **3 (A)** and **4 (B)** by a 10-fold excess of ascorbic acid. Observed as a series of ^1^H NMR spectra over 72 h **(A)** and 24 h **(B)** in a mixture 1/1 of DMSO-d_6_ and PBS (pH = 6.8). Peaks represent the triazole protons of complex **3 (A)** and **4 (B)**, which over time are converted to the triazole protons of their corresponding ligands **18** and **20**, respectively. Showing a complete cleavage of the axial carbohydrate ligand.

The complexes were stable in aqueous condition (DMSO/HEPES Buffer 4/6) for up to 4 days at r.t. For a confirmation of the activation by intracellular reduction that occur in Pt (IV) species, the behaviour of the Pt (IV) prodrugs was studied by the addition of ascorbic acid into a solution of complexes **3** and **4** (DMSO-PBS buffer 1/1), taken as reference for an acetylated and a free sugar derivatives (Nokami et al., 2009). The reduction process was followed by ^1^H-NMR with complete release of the carbohydrate carboxylic acids axial ligand **18** and **20** after 72 h (**3**) and 24 h (**4**). This can be followed by an upfield movement in chemical shift of the singlet triazole peak (Figure 3 and Supplementary Material).

### Biological evaluation

To comprehensively evaluate the biological activity of C2-functionalised glycoconjugates Pt (IV) prodrugs a screening of the anticancer effect of the complexes was performed using different cell lines in 2D and 3D scaffold-based *in vitro* models, including breast cancer (MDAMB 468), osteosarcoma (U2-OS, SOAS-2 and MG63) and glioblastoma (U87) (Giusto et al., 2022).

**FIGURE 4:**
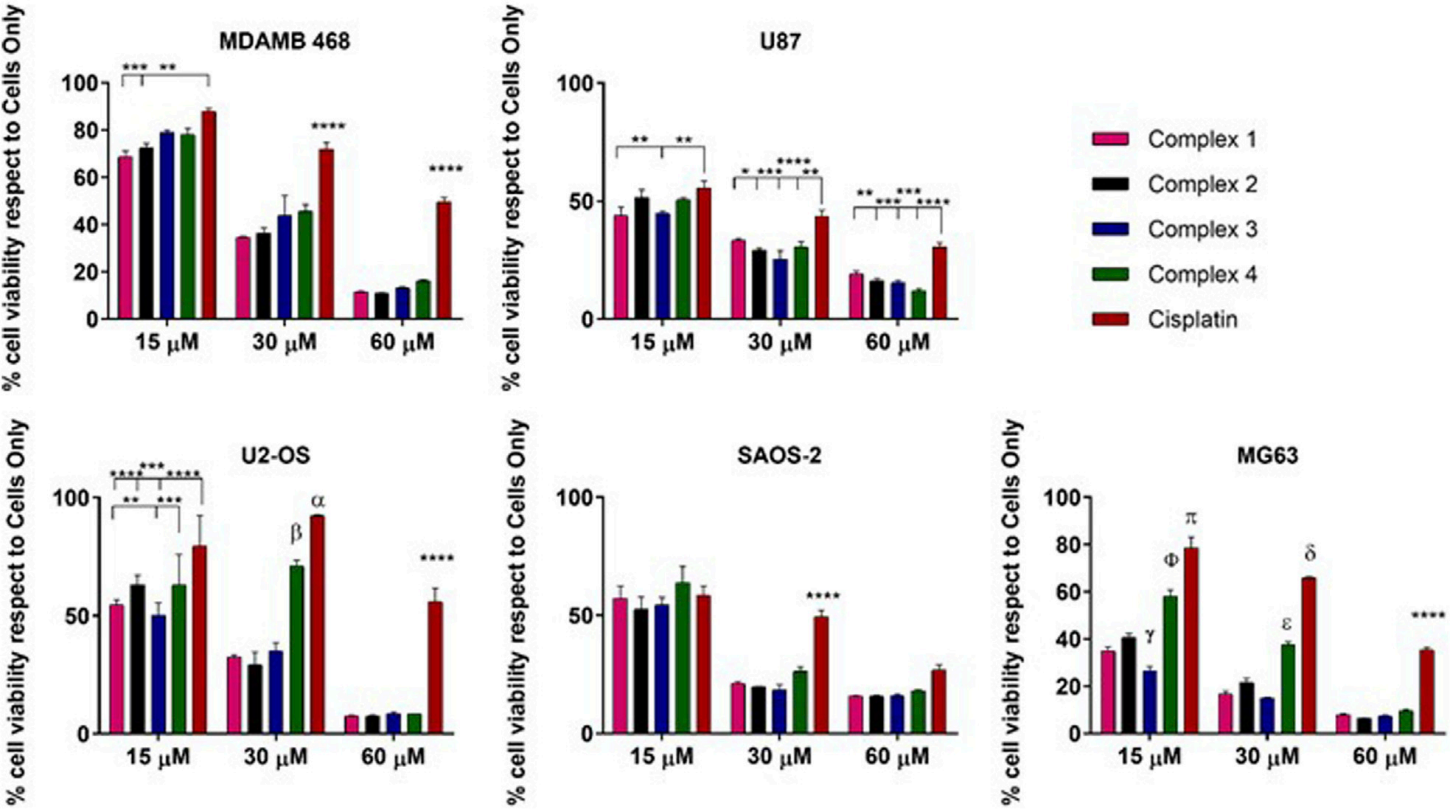
2D *in vitro* drug screening by MTT assay. Cell viability evaluation after 72 h of incubation with complexes. Data are reported in the graph as percentage (%) mean ± standard error of the mean. Statistical analyses are reported in the graph, *p-value ≤ 0.05; **p-value ≤ 0.01; ***p-value ≤ 0.001 and ***p-value ≤ 0.0001. α: cisplatin vs. complex 4 *p*-value ≤ 0.01 and vs. complex 1, 2, 3 *p*-value ≤ 0.0001; β: complex 4 respects to complex 1, 2, 3 *p*-value ≤ 0.0001. γ: complex 3 vs. all the drugs *p*-value ≤ 0.0001 and complex 3 vs. 1 *p*-value ≤ 0.05; ɸ: complex 4 vs. all *p*-value ≤ 0.000; π: cisplatin vs. all *p*-value ≤ 0.0001; δ: cisplatin vs. all *p*-value ≤ 0.0001; ε: complex 4 vs. all *p*-value ≤ 0.0001.

As expected, the cell viability evaluation demonstrated a dose-dependent cytotoxic effect of all the drugs affecting all the cancer cell types, with weak differences depending on the cell phenotypes (Figure 4).

Looking in detail at the effect of the proposed complexes and comparing each other with the reference drug cisplatin, it is possible to observe an increased anticancer effect exerted by all the complexes compared to cisplatin. Even at a low concentration (15 µM), the complexes exhibited a higher cytotoxic impact compared to the reference in all cell lines, with exceptions noted in the SAOS2 cell line. Statistically significant differences were observed in MDAMB 48 cells (complexes **1** and **2**), U87 cells (complexes **1** and **3**), U2-OS cells (complexes **1, 2**, and **3**), and MG63 cells (complexes **1–4**). Notably, complex **3** demonstrated higher efficacy also compared to the other proposed complexes in U2-OS and MG63 cells, with this effect strengthened at 30 µM relative to cisplatin and complex **4**. At a concentration of 60 μM, the complexes consistently exhibited higher toxicity compared to cisplatin, with statistically significant differences. However, at this elevated concentration, discernible distinctions among the complexes were not observed, a trend consistent across all cell lines except SAOS-2, where no differences were detected (Figure 1). The different cellular behaviour observed among the osteosarcoma cell lines can be attributed to the intrinsic biological differences of the three phenotypes used as models. (Visvader, 2011).

Even if complexes **1**, **2** and **3** are acetylated derivatives, these results suggest there may be an involvement of the GLUTs receptor in the internalization and related anticancer effect of the cisplatin glycoconjugates. It was reasonable to hypothesize that the presence of the C2-functionalised glucosamine-conjugates, increases the drug uptake in the cancer cells overexpressing the GLUTs receptor, as already demonstrated in all the cancer cell lines used, osteosarcoma cells, MDAMB 468 and U87 (Adekola et al., 2012). The observed behaviour of SAOS-2 cells, where the differences of the complex effects with respect to cisplatin are negligible, strengthens the role of the GLUT receptor in the increased toxic effect of the complexes. In fact, it has been demonstrated that the GLUT1 mRNA level was lower in SAOS-2 if compared to MG63 and U2-OS (Cifuentes et al., 2011).

To deeply investigate the strongest effect shown from complex **3,** a proof of concept *in vitro* was performed using a more relevant 3D scaffold-based cancer model. MG63 cell line was cultured on a 3D scaffold that mimics the feature of the bone extracellular matrix, as previously demonstrated (Krishnakumar et al., 2018; Bassi et al., 2020). Together with complex **3**, we decided to study the behaviour on 3D of complex **4** that is the analogue of **3** with the free sugar (not acetylated) to shed further light on the role of the carbohydrate moiety.

**FIGURE 5:**
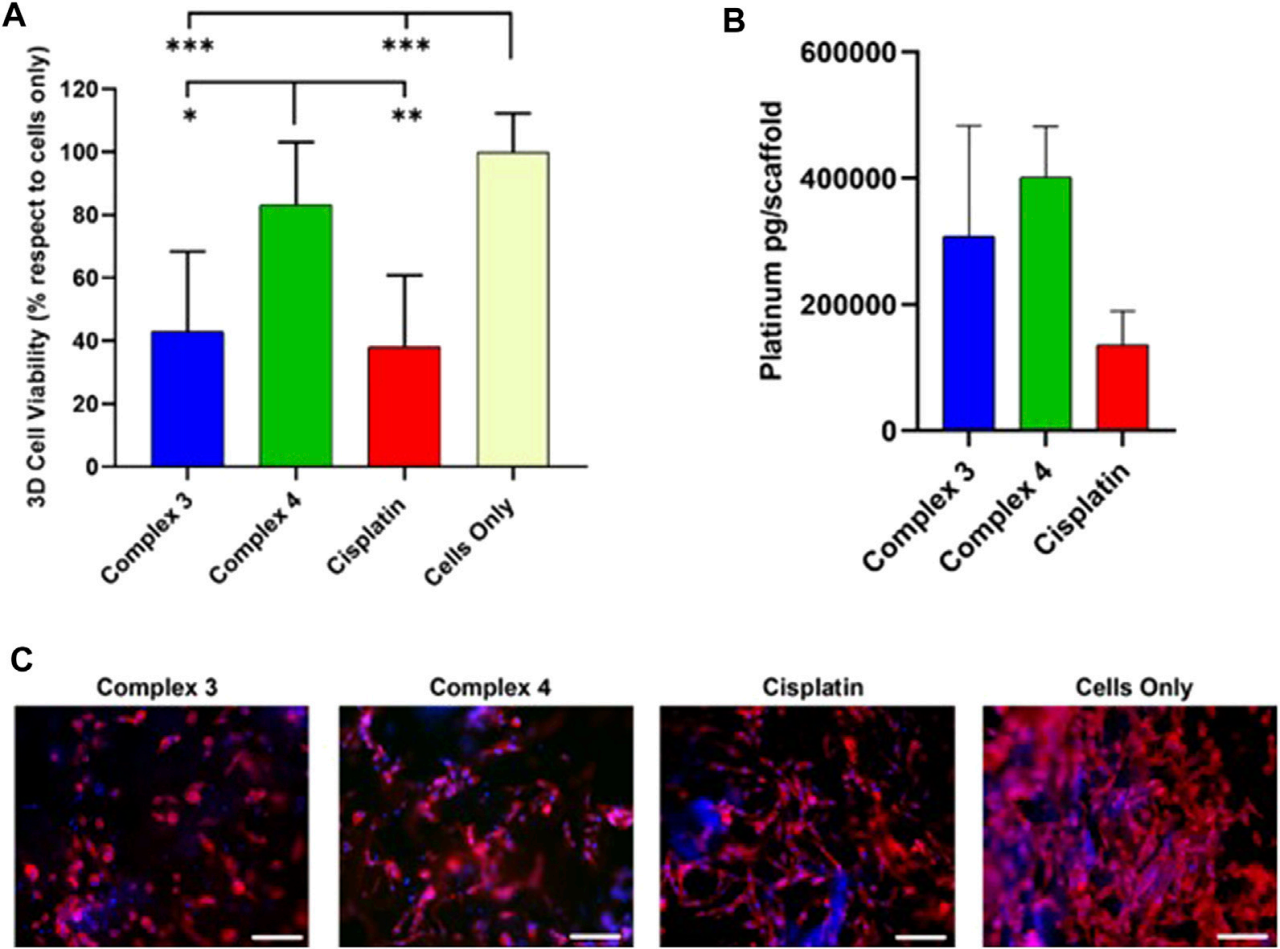
Effect of complexes 3 and 4 complexes on cellular behaviors and uptake in 3D MG63 model. **(A)** Cell viability evaluation after 72 h of incubation with 30 µm of complexes by MTT assay. Data are reported in the graph as percentage (%) mean ± standard error of the mean. Statistical analyses are reported in the graph, *p-value ≤ 0.05; **p-value ≤ 0.01; ***p-value ≤ 0.001. **(B)** ICP-OES analysis of the cell internalized Cisplatin, mean ± standard error. **(C)**. one respective image for each drug and cells only shows cell morphology; phalloidin in red stains for actin filaments and DAPI in blue stains for cell nuclei. Scale bars 200 µm.

The cell viability evaluation confirmed the anticancer effect of **3** (very similar to cisplatin) that shows higher activity with respect complex **4**, in line with what was observed on the 2D studies (Figures 5A,C). To confirm the role of the carbohydrate, we studied the drug uptake by ICP-OES analysis. The results showed that both the tested complexes **3** and **4** are highly internalized by the cells compared to the cisplatin, with the highest cellular internalisation observed for complex **4** possessing the free carbohydrate moiety, supporting the role of the GLUT receptor in the cellular uptake (Figure 5B).

The apparent discrepancy between cytotoxicity and uptake results between complexes **3** and **4** can be rationalised by analysing the reduction behaviour of the two complexes in the presence of ascorbic acid (Figure 3). As discussed earlier, complex **3** is reduced in 72 h while complex **4** only in 24 h. Even if complex **4** is internalised better than **3** as expected due to the presence of the free carbohydrate moiety, once inside the cytoplasm it is quicker reduced, forming cisplatin in 24 h. On the other hand, complex **3** takes 72 h to completely release cisplatin after internalisation and this slow release could explain the higher activity of complex **3** with respect to **4** that releases cisplatin too quickly (cytotoxicity studies were performed at 72 h). The morphological analysis (Figure 5C) reveals that the cells successfully grow and colonize the scaffolds, integrating with the bone mimetic matrix. They exhibit the expected nuclear and cytoplasmic morphologies, along with cytoplasmic extensions characteristic of cells interacting with biomimetic materials. A noticeable decrease in cell number and a decline in cell morphology are observed in the 3D cell culture treated with the complexes and the reference drug compared to untreated cells (cells only). This observation confirms the anticancer activity and viability results.

## Conclusion

In this paper, we have reported the synthesis and characterisation of a series of Pt (IV) complexes based on cisplatin scaffold tethering in axial position a glucosamine moiety conjugated through the C2 position of the sugar ring. While different examples of Pt-glycoconjugates complexes are reported in literature, also by us (all conjugated via the anomeric C1 position), this series represents the first example of Pt (IV) prodrugs conjugated with C2-functionalised glucosamine derivatives. C2 is an attractive position for drug conjugation proven by the fact that 2-deoxyglucose is transported by all class 1 GLUTs and examples of Pt (II) C2 conjugates supported this point. The complexes are very active against a panel of different cancer cell lines including breast cancer (MDAMB 468), osteosarcoma (U2-OS, SOAS-2 and MG63) and glioblastoma (U87). The same very promising results have been obtained also in 3D MG63 cell lines. In 3D, the drug uptake of the selected complexes **3** and **4** is higher with respect to that one of cisplatin indicating a role of the carbohydrate moiety in the drug internalisation. Even if a direct comparison with the other Pt (IV) C1-functionalised glycoconjugates previously reported by us is very difficult to discuss due to the different structures and cell lines used, the role of the carbohydrate is demonstrated in term of higher cellular uptake and higher anticancer activity.

## Data Availability

The original contributions presented in the study are included in the article/Supplementary Material, further inquiries can be directed to the corresponding authors.

## In memoriam

Dedicated to Jan Belza, a Ph.D. candidate of this consortium who deceased too soon.
