# Supplementary material for: 2D and 3D anticancer properties of C2-functionalised glucosamine-Pt (IV) prodrugs based on cisplatin scaffold
Source: Front Chem. 2024 May 6;12:1388332. doi: 10.3389/fchem.2024.1388332 (PMC11102980; doi:10.3389/fchem.2024.1388332)
Supplement: Supplementary file 1 [file DataSheet1.PDF]

Supplementary Information

2D and 3D Anticancer properties of C2-functionalised glucosamine-Pt(IV) prodrugs based on cisplatin scaffold

Eoin Moynihan, Maria Galiana Cameo, Monica Sandri, Andrea Ruffini, Silvia Panseri, Trinidad Velasco-Torrijos, Monica Montesi, Diego Montagner

Synthetic procedures

*1,3,4,6-Tetra-O-acetyl-2-azido-2-deoxy-D-glucopyranoside (**6**)*

A solution of NaN_3 (8.94 g, 137 mmol) in H_2O (22 mL) was cooled to 0°C and stirred for 5 mins and DCM (40 mL) was added. The mixture was stirred vigorously and Tf_2O (4.68 mL, 27.90 mmol) added over a period of 5 mins, and the reaction stirred at 0°C for 2 h. The organic phase was separated, and the aqueous phase was washed with DCM (35 mL). The combined organic phases were washed with saturated sodium carbonate solution. The total volume of TfN_3 in DCM was 75 mL, and this reagent solution was used without further purification. **5** (3.0 g, 13.95 mmol) was dissolved in H_2O (45 mL) and treated with solid K_2CO_3 (2.88 g, 20.93 mmol) and $CuSO_4$ (21 mg, 132 mmol). Methanol (90 mL) was added followed by the dropwise addition of the freshly prepared TfN_3 solution. Methanol was added until the solution was homogeneous, and the reaction was then stirred overnight at room temperature. The solvent was removed under vacuum and the residue was redissolved in pyridine (75 mL). This solution was then cooled to 0°C and Ac_2O (45 mL) was added, and the mixture was allowed to stir at room temperature overnight. Solvent was removed under vacuum and the residue was redissolved in EtOAc (300 mL) the organic phase was washed with saturated $CuSO_4$ solution (200 mL×2) and saturated $NaHCO_3$ solution (200 mL) and the organic phase was dried with Na_2SO_4 and filtered. The solvent was removed under vacuum to give a residue which was purified by column chromatography (EtOAc: Petroleum ether=3:7) to give the product as a colourless syrup (3.848 g, 10.31 mmol, 74%). ^{1}H NMR (500 MHz, $CDCl_3$) δ 6.27 (d, *J* = 3.7 Hz, 0.37H, H-1α), 5.54 (d, *J* = 8.6 Hz, 0.63H, H-1β), 5.43 (dd, *J* = 10.4, 9.5 Hz, 0.39H, H-3α), 5.12 – 4.99 (m, 1.6H, H-3β, H-4), 4.27 (dt, *J* = 12.6, 4.5 Hz, 1H, H-6), 4.08 – 4.00 (m, 1.38H, H-5α, H-6’), 3.79 (ddd, *J* = 9.7, 4.5, 2.1 Hz, 0.61H, H-5β), 3.65 (ddd, *J* = 8.6, 5.8, 2.1 Hz, 0.98H, H-2), 2.18 – 1.99 (m, 12H, 4 x CH_3 of OAc) ppm.

NMR data is in agreement with the data reported in the literature [Cheng, J.; Zhao, W.; Yao, H.; Shen, Y.; Zhang, Y.; Li, Y.Z.; Qi, Q.; Wongprasert, K.; Tang, Y.J. Discovery of 4,6- O-Thenylidene-β- d -Glucopyranoside-(2″-Acetamido, 3″-Acetyl-Di- S-5-Fluorobenzothizole/5-Fluorobenzoxazole)-4′-Demethylepipodophyllotoxin as Potential Less Toxic Antitumor Candidate Drugs by Reducing DNA Damage and Less Inhibition of PI3K. *J Med Chem* **2020**, *63*, 2877–2893, doi:10.1021/acs.jmedchem.9b01354].

*N-(1,3,4,6-Tetra-O-acetyl-2-deoxy-D-glucopyranosyl-1,2,3-triazol-4-yl)-propanoic acid (**7**)*

6 (0.989 g, 2.649 mmol, 1.3 equiv.) and 4-pentynoic acid (0.2 g, 2.03 mmol) were dissolved in a mixture of tetrahydrofuran (6 ml), tert-Butanol (6 mL) and deionised water (4 mL). Separately, copper(II) sulphate pentahydrate (0.1 g, 0.40 mmol) and sodium ascorbate (0.161 g, 0.815 mmol) were dissolved in deionised water (2 mL), added to reaction flask and allowed to stir at r.t. overnight (16 h). The solvent was removed *in vacuo* and the residue was dissolved in DCM (15 mL) and washed with brine (2 x 20 mL). The organic phase was dried with $MgSO_4$, filtered and the solvent was evaporated. The crude product was purified by column chromatography (1:1, petroleum ether : ethyl acetate) to yield a white solid (0.566 g, 1.20 mmol, 59%). R_f = 0.18 (95:5 DCM : MeOH). ^{1}H NMR (500 MHz, $CDCl_3$) δ 7.47 (s, 0.52H, triaz-*H*α), 7.45 (s, 1H, triaz-*H*β), 6.36 (d, *J* = 3.6 Hz, 0.51H, H-1α), 6.18 (d, *J* = 8.8 Hz, 1H, H-1β), 5.92 (dd, *J* = 11.4, 9.2 Hz, 0.61H, H-3α), 5.77 (dd, *J* = 10.8, 9.2 Hz, 1H, H-3β), 5.26 (dd, *J* = 10.2, 9.3 Hz, 0.64H, H-4α), 5.20 (dd, *J* = 10.1, 9.3 Hz, 1H, H-4β), 5.13 (dd, *J* = 11.4, 3.6 Hz, 0.55H, H-2α), 4.67 (dd, *J* =

10.8, 8.8 Hz, 1H, H-2β), 4.40 – 4.32 (m, 1.51H, H-6α, H-6β), 4.20 (ddd, *J* = 10.3, 4.0, 2.3 Hz, 0.55H, H-5α), 4.13 (ddd, *J* = 19.2, 12.5, 2.2 Hz, 1.54H, H-6'α, H-6'β), 4.07 (ddd, *J* = 10.1, 4.4, 2.2 Hz, 1H, H-5β), 3.01 (q, *J* = 6.9 Hz, 3H, α/β triaz-C*H₂*), 2.74 (t, *J* = 6.9 Hz, 3H, α/β C*H₂*CO), 2.11 (s, 1.5H, α CH_3 of OAc), 2.10 (d, *J* = 2.2 Hz, 4.5H, α CH_3 of OAc, β CH_3 of OAc), 2.06 (s, 1.5H, α CH_3 of OAc), 2.03 (s, 3H, β CH_3 of OAc), 1.97 (s, 3H, β CH_3 of OAc), 1.85 (d, *J* = 3.5 Hz, 4.5H, β CH_3 of OAc, α CH_3 of OAc) ppm. ^{13}C NMR (125 MHz, $CDCl_3$) δ 176.68 (*C*OOH), 170.75 (α *C*O of OAc), 170.74 (β *C*O of OAc), 170.17 (α *C*O of OAc), 169.76 (β *C*O of OAc), 169.48 (α *C*O of OAc), 169.44 (β *C*O of OAc), 168.40 (β *C*O of OAc), 168.08 (α *C*O of OAc), 146.65 (triaz-*C*α), 146.49 (triaz-*C*β), 121.74 (triaz-*C*Hβ), 120.67 (triaz-*C*Hα), 91.69 (C-1β), 90.20 (C-1α), 73.03 (C-5β), 72.10 (C-3β), 69.98 (C-5α), 68.92 (C-3α), 68.31 (C-4β), 68.20 (C-4α), 62.76 (C-2β), 61.54 (C-6β), 61.47 (C-6α), 61.13 (C-2α), 33.43 (α/β *C*H_2CO), 20.83 (*C*H_3 of OAc), 20.80 (2 x *C*H_3 of OAc), 20.78 (*C*H_3 of OAc), 20.68 (*C*H_3 of OAc), 20.65 (*C*H_3 of OAc), 20.60 (α/β triaz-*C*H_2), 20.41 (*C*H_3 of OAc), 20.30 (*C*H_3 of OAc) ppm. IR (ATR) 3141.55, 1753.04, 1736.47, 1367.40, 1207.16, 1026.92, 897.06 cm^{-1}. HR-MS (+): m/z calcd for $C_{19}H_{25}N_3O_{11}$ + H^+ $[M+H]^+$ 472.1567, found 472.1607. HR-MS (+): m/z calcd for $C_{19}H_{25}N_3O_{11}$ + Na^+ $[M+Na]^+$ 494.1387, found 494.1431.

*N-(1,3,4,6-Tetra-O-acetyl-2-deoxy-D-glucopyranosyl-1,2,3-triazol-4-yl)-(3-oxopropyl-(oxy(2,5-dioxopyrrolidin-1-yl))) (**8**)*

7 (0.120 g, 0.254 mmol) was added to a flask with NHS (0.035 g, 0.305 mmol, 1.2 equiv.), purged with N_2 and dissolved in dry DCM. EDCI (0.058 g, 0.305 mmol, 1.2 equiv.) was dissolved in DCM and added *via* cannula. The reaction was stirred overnight at room temperature. The DCM was washed with 0.1M HCl, brine and dried with Na_2SO_4. The solvent was filtered and evaporated to yield a white solid which was used without further purification (0.130 g, 0.228 mmol, 89%) (The ratio of alpha:beta was 1:0.51). R_f = 0.69 (95:5 DCM : MeOH). ^{1}H NMR (500 MHz, $CDCl_3$) δ 7.65 (s, 1H, triaz-*H*β), 7.63 (s, 0.84H, triaz-*H*α), 6.31 (d, *J* = 3.5 Hz, 0.81H, H-1α), 6.10 (d, *J* = 8.8 Hz, 1H, H-1β), 5.88 (dd, *J* = 11.4, 9.2 Hz, 0.86H, H-3α), 5.71 (dd, *J* = 10.8, 9.2 Hz, 1H, H-3β), 5.19 (ddd, *J* = 21.6, 10.1, 9.3 Hz, 1.93H, H-4β, H-4α), 5.09 (dd, *J* = 11.4, 3.6 Hz, 0.88H, H-2α), 4.66 (dd, *J* = 10.8, 8.8 Hz, 1H, H-2β), 4.32 (ddd, *J* = 19.6, 12.5, 4.2 Hz, 1.9H, H-6α, H-6β), 4.18 (ddd, *J* = 10.3, 3.9, 2.3 Hz, 0.89H, H-5α), 4.09 (ddd, *J* = 18.8, 12.5, 2.2 Hz, 1.91H, H-6'α, H-6'β), 4.03 (ddd, *J* = 10.2, 4.3, 2.2 Hz, 1H, H-5β), 3.16 – 3.07 (m, 3.67H, α/β triaz-C*H₂*), 3.00 – 2.89 (m, 3.76H, α/β C*H₂*CO), 2.81 (s, 7.52H, α/β CH_2CH_2-Succ), 2.07 (s, 2.57H, α CH_3 of OAc), 2.06 (s, 5.43H, α/β CH_3 of OAc), 2.01 (s, 2.54H, α CH_3 of OAc), 1.99 (s, 3H, β CH_3 of OAc), 1.92 (s, 3H, β CH_3 of OAc), 1.81 (s, 3H, β CH_3 of OAc), 1.79 (s, 2.35H, α CH_3 of OAc) ppm. ^{13}C NMR (125 MHz, $CDCl_3$) δ 170.61 (α *C*O of OAc), 170.59 (β *C*O of OAc), 169.87 (α *C*O of OAc), 169.60 (β *C*O of OAc), 169.40 (α *C*O of OAc), 169.32 (β *C*O of OAc), 169.27 (α/β *C*O-Succ), 168.30 (β *C*O of OAc), 168.01 (α *C*O of OAc), 167.72 (*C*O β), 167.68 (*C*O α), 145.20 (triaz-*C*α), 145.16 (triaz-*C*β), 121.91 (triaz-*C*Hβ), 121.20 (triaz-*C*Hα), 91.59 (C-1β), 90.07 (C-1α), 72.84 (C-5β), 71.97 (C-3β), 69.78 (C-5α), 68.85 (C-3α), 68.36 (C-4α), 68.12 (C-4β), 62.61 (C-2β), 61.42 (C-6β), 61.40 (C-6α), 60.96 (C-2α), 31.16 (β *C*H_2CO), 31.13 (α *C*H_2CO), 25.64 (β *C*H_2*C*H2-Succ), 25.63 (α *C*H_2*C*H2-Succ), 21.10 (α triaz-*C*H_2), 21.05 (β triaz-*C*H_2), 20.73 (β *C*H_3 of OAc), 20.71 (α *C*H_3 of OAc), 20.69 (α *C*H_3 of OAc), 20.59 (β *C*H_3 of OAc), 20.57 (α *C*H_3 of OAc), 20.50 (β *C*H_3 of OAc), 20.31 (α *C*H_3 of OAc), 20.21 (β *C*H_3 of OAc) ppm. IR (ATR) 2959.88, 1731.79, 1366.73, 1201.79, 1067.81, 1043.20, 813.86 cm^{-1}. HR-MS (+): m/z calcd for $C_{23}H_{28}N_4O_{13}$ + H^+ $[M+H]^+$ 569.1731, found 569.1800. HR-MS (+): m/z calcd for $C_{23}H_{28}N_4O_{13}$ + Na^+ $[M+Na]^+$ 591.1551, found 591.1631.

*Methyl-N-(3,4,6-tri-O-acetyl-2-deoxy-α-D-glucopyranosyl-1,2,3-triazol-4-yl)-propanoic acid (**14**)*

13 (0.469 g, 1.35 mmol) and 4-pentynoic acid (0.264 g, 2.7 mmol, 2 equiv.) were dissolved in t-BuOH (12mL), H_2O (12mL) and THF (12mL). $CuSO_4$ (195 mg) and NaAsc (275 mg) were added and allowed to stir overnight. Completion of the reaction was followed by TLC (DCM:MeOH. 9:1). The solvent was then removed *in vacuo* and the resulting residue was redissolved in DCM and washed with brine. The organic layer was dried and filtered, and the crude product was purified by column chromatography (DCM:MeOH. 95:5). The pure product was obtained as a colourless oil (0.394 g, 0.888 mmol, 65%). R_f = 0.37 (95:5 DCM:MeOH). ^{1}H NMR (500 MHz, $CDCl_3$) δ 7.57 (s, 1H, triaz-C*H*), 5.75 (dd, *J* = 11.2, 9.1 Hz, 1H, H-3), 5.18 (dd, *J* = 10.1, 9.2 Hz, 1H, H-4), 5.01 (dd, *J* = 11.3, 3.4 Hz, 1H, H-2), 4.96 (d, *J* = 3.4 Hz, 1H,

H-1), 4.31 (dd, J = 12.4, 4.7 Hz, 1H, H-6), 4.16 (dd, J = 12.3, 2.4 Hz, 1H, H-6'), 4.11 (ddd, J = 10.2, 4.7, 2.3 Hz, 1H, H-5), 3.39 (s, 3H, OCH_3), 3.02 (t, J = 7.3 Hz, 2H, triaz-CH_2), 2.75 (t, J = 7.4 Hz, 2H, CH_2CO), 2.12 (s, 3H, CH_3 of OAc), 2.04 (s, 3H, CH_3 of OAc), 1.80 (s, 3H, CH_3 of OAc) ppm. ^{13}C NMR (125 MHz, CDCl3) δ 177.04 (*C*O), 170.79 (*C*O of OAc), 170.18 (*C*O of OAc), 169.55 (*C*O of OAc), 146.31 (triaz-*C*), 121.14 (triaz-*C*H), 98.10 (C-1), 69.41 (C-3), 68.77 (C-4), 67.88 (C-5), 62.29 (C-2), 61.94 (C-6), 55.77 (O*C*H_3), 33.51 (*C*H_2CO), 20.91 (triaz-*C*H_2), 20.85 (*C*H_3 of OAc), 20.68 (*C*H_3 of OAc), 20.35 (*C*H_3 of OAc) ppm. IR (ATR) 2943.14, 1739.49, 1367.31, 1217.89, 1032.28, 927.19, 733.50, 601.37 cm^{-1}. HR-MS (+): m/z calcd for $C_{18}H_{25}N_3O_{10}$ + H^+ $[M+H]^+$ 444.1618, found 444.1616. HR-MS (+): m/z calcd for $C_{18}H_{25}N_3O_{10}$ + Na^+ $[M+Na]^+$ 466.1438, found 466.1433.

*Methyl-N-(3,4,6-tri-O-acetyl-2-deoxy-α-D-glucopyranosyl-1,2,3-triazol-4-yl)-(3-oxopropyl-(oxy(2,5-dioxopyrrolidin-1-yl))) (**15**)*

14 (0.240 g, 0.541 mmol) and NHS (0.074 g, 0.649 mmol, 1.2 equiv.) were purged with N_2 and dissolved in anhydrous DCM (10 mL). A separate flask containing EDCI (0.124 g, 0.649 mmol, 1.2 equiv.) was also purged with N_2 and dissolved in anhydrous DCM (4 mL). This was added to the solution of **14** and NHS via cannula addition and the reaction was allowed to stir overnight at room temperature. The organic phase was washed with 0.1M HCl, dried, filtered and the solvent was evaporated and isolated as a white powder (0.267 g, 0.494 mmol, 91%). R_f = 0.77 (95:5 DCM:MeOH). ^{1}H NMR (500 MHz, $CDCl_3$) δ 7.68 (s, 1H, triaz-C*H*), 5.74 (dd, J = 11.2, 9.1 Hz, 1H, H-3), 5.18 (dd, J = 10.1, 9.2 Hz, 1H, H-4), 5.02 (dd, J = 11.2, 3.4 Hz, 1H, H-2), 4.98 (d, J = 3.4 Hz, 1H, H-1), 4.32 (dd, J = 12.3, 4.6 Hz, 1H, H-6), 4.17 (dd, J = 12.3, 2.4 Hz, 1H, H-6'), 4.13 (ddd, J = 10.3, 4.6, 2.4 Hz, 1H, H-5), 3.40 (s, 3H, OCH_3), 3.15 (td, J = 7.2, 2.4 Hz, 2H, triaz-CH_2), 3.01 (td, J = 7.2, 2.4 Hz, 2H, CH_2CO), 2.83 (s, 4H, CH_2CH_2-succ), 2.12 (s, 3H, CH_3 of OAc), 2.04 (s, 3H, CH_3 of OAc), 1.81 (s, 3H, CH_3 of OAc) ppm. ^{13}C NMR (125 MHz, $CDCl_3$) δ 170.78 (*C*O of OAc), 170.07 (*C*O of OAc), 169.57 (*C*O of OAc), 169.16 (*C*O succ x2), 167.80 (*C*O), 145.16 (triaz-*C*), 121.50 (triaz-*C*H), 98.13 (C-1), 69.49 (C-3), 68.90 (C-4), 67.84 (C-5), 62.30 (C-2), 61.96 (C-6), 55.80 (O*C*H_3), 31.12 (*C*H_2CO), 25.74 (*C*H_2*C*H_2-succ), 21.13 (triaz-*C*H_2), 20.87 (*C*H_3 of OAc), 20.72 (*C*H_3 of OAc), 20.42 (*C*H_3 of OAc) ppm. IR (ATR) 2962.23, 1731.98, 1366.80, 1204.22, 1029.25, 799.23 cm^{-1}. HR-MS (+): m/z calcd for $C_{22}H_{28}N_4O_{12}$ + H^+ $[M+H]^+$ 541.1782, found 541.1817. HR-MS (+): m/z calcd for $C_{22}H_{28}N_4O_{12}$ + Na^+ $[M+Na]^+$ 563.1601, found 563.1641.

*Methyl-2-(2-bromoacetamido)-2-deoxy-3,4,6-tri-O-acetyl-α-D-glucopyranoside (**16**)*

12 (2.305 g, 7.21 mmol) was purged with N_2 and dissolved in dry DCM (19 mL). NEt_3 (1.2 mL, 8.66 mmol, 1.2 equiv) was added and the solution was allowed to stir over ice. Bromoacetyl bromide (0.75 mL, 8.66 mmol, 1.2 equiv.) was diluted with dry DCM (5 mL) and added to the flask *via* cannula. The reaction was stirred overnight, and the crude product was extracted with 1M HCl, aqueous $NaHCO_3$, brine and dried with Na_2SO_4. Yielding a white solid (2.279 g, 5.176 mmol, 71%). R_f = 0.73 (petroleum ether: EtOAc 1:1). ^{1}H NMR (500 MHz, $CDCl_3$) δ 6.56 (d, J = 9.4 Hz, 1H, N*H*CO), 5.25 (dd, J = 10.7, 9.5 Hz, 1H, H-3), 5.09 (t, J = 10.2 Hz, 1H, H-4), 4.74 (d, J = 3.6 Hz, 1H, H-1), 4.31 – 4.20 (m, 2H, H-2, H-6), 4.09 (dd, J = 12.3, 2.4 Hz, 1H, H-6'), 3.93 (ddd, J = 10.1, 4.7, 2.3 Hz, 1H, H-5), 3.78 (d, J = 2.6 Hz, 2H, CH_2Br), 3.41 (s, 3H, OCH_3), 2.08 (s, 3H, CH_3 of OAc), 2.01 (s, 3H, CH_3 of OAc), 1.99 (s, 3H, CH_3 of OAc) ppm. ^{13}C NMR (125 MHz, $CDCl_3$) δ 171.17 (*C*O of OAc), 170.77 (*C*O of OAc), 169.43 (*C*O of OAc), 166.03 (NH*C*O), 98.04 (C-1), 70.93 (C-3), 68.15 (C-4), 67.78 (C-5), 62.02 (C-6), 55.65 (O*C*H_3), 52.58 (C-2), 28.45 (*C*H_2Br), 20.81 (2x *C*H_3 of OAc), 20.69 (*C*H_3 of OAc) ppm. IR (ATR) 3246.56, 1734.05, 1651.68, 1552.68, 1363.83, 1211.90, 1131.38, 1056.20, 1029.13, 924.95 cm^{-1}. HR-MS (+): m/z calcd for $C_{15}H_{22}BrNO_9$ + Na^+ $[M+Na]^+$ 462.0376, found 462.0319.

*Methyl-2-(2-azidoacetamido)-2-deoxy-3,4,6-tri-O-acetyl-α-D-glucopyranoside (**17**)*

16 (1.818 g, 4.12 mmol) and NaN_3 (0.536 g, 8.25 mmol, 2 equiv.) were dissolved in anhydrous DMF (24 mL) and refluxed at 80°C overnight. DMF was evaporated and the residue was redissolved in DCM and washed with cold water. The organic phase was dried with Na_2SO_4, filtered and dried *in vacuo* to yield a white solid (1.634 g, 4.06 mmol, 98%). R_f = 0.72 (petroleum ether: EtOAc 1:1). ^{1}H NMR (500 MHz,

$CDCl_3$) δ 6.50 (d, *J* = 9.5 Hz, 1H, N*H*CO), 5.25 (dd, *J* = 10.6, 9.5 Hz, 1H, H-3), 5.12 (t, *J* = 9.8 Hz, 1H, H-4), 4.74 (d, *J* = 3.6 Hz, 1H, H-1), 4.32 (ddd, *J* = 10.6, 9.5, 3.7 Hz, 1H, H-2), 4.25 (dd, *J* = 12.3, 4.6 Hz, 1H, H-6), 4.11 (dd, *J* = 12.3, 2.4 Hz, 1H, H-6'), 3.96 – 3.91 (m, 3H, H-5, C$H_2$$N_3$), 3.42 (s, 3H, OC$H_3$), 2.10 (s, 3H, C$H_3$ of OAc), 2.03 (s, 3H, CH_3 of OAc), 2.01 (s, 3H, CH_3 of OAc) ppm. ^{13}C NMR (125 MHz, $CDCl_3$) δ 171.31 (*C*O of OAc), 170.81 (*C*O of OAc), 169.47 (*C*O of OAc), 166.84 (NH*C*O), 98.10 (C-1), 71.27 (C-3), 68.16 (C-4), 67.78 (C-5), 62.08 (C-6), 55.63 (O*C*H_3), 52.66 (*C*H_2N_3), 52.07 (C-2), 20.85 (*C*H_3 of OAc), 20.82 (*C*H_3 of OAc), 20.73 (*C*H_3 of OAc) ppm. IR (ATR) 3332.36, 2104.64, 1736.94, 1670.34, 1542.60, 1368.04, 1217.17, 1030.83, 923.96 cm^{-1}. HR-MS (+): m/z calcd for $C_{15}H_{22}N_4O_9$ + H^+ $[M+H]^+$ 403.1465, found 403.1417. HR-MS (+): m/z calcd for $C_{15}H_{22}N_4O_9$ + Na^+ $[M+Na]^+$ 425.1284, found 425.1237.

*Methyl-N-(2-deoxy-3,4,6-tri-O-acetyl-α-D-glucopyranosyl)-2-acetamido-1,2,3-triazol-4-yl)]-propanoic acid (**18**)*

17 (0.525 g, 1.304 mmol) and 4-Pentynoic acid (0.191 g, 1.956 mmol, 1.5 equiv.) were dissolved in 15 mL each of t-BuOH and H_2O. Sodium ascorbate (0.183 g, 0.92 mmol) and $CuSO_4.5H_2O$ (0.140 g, 0.57 mmol) were added to the solution. This was allowed to stir at room temperature overnight. The solvent was removed *in vacuo* and the residue was redissolved in DCM and washed with brine. The organic phase was dried with Na_2SO_4 and filtered. The crude product was purified with column chromatography (DCM:MeOH 9:1) to yield a white solid (0.267 g, 0.723 mmol, 41%). R_f = 0.78 (90:10 DCM:MeOH). ^{1}H NMR (500 MHz, $CDCl_3$) δ 7.55 (s, 1H, triaz-C*H*), 6.72 (d, *J* = 9.0 Hz, 1H, N*H*CO), 5.16 (t, *J* = 9.9 Hz, 1H, H-3), 5.06 (t, *J* = 9.8 Hz, 1H, H-4), 4.96 (q, *J* = 16.3 Hz, 2H, NHCOCH_2), 4.71 (d, *J* = 3.5 Hz, 1H, H-1), 4.30 (td, *J* = 10.1, 3.6 Hz, 1H, H-2), 4.24 (dd, *J* = 12.4, 4.7 Hz, 1H, H-6), 4.07 (dd, *J* = 12.3, 2.2 Hz, 1H, H-6'), 3.92 (ddd, *J* = 10.0, 4.5, 2.3 Hz, 1H, H-5), 3.37 (s, 3H, OCH_3), 3.01 (br s, 2H, triaz-CH_2), 2.73 (br s, 2H, CH_2CO), 2.07 (s, 3H, CH_3 of OAc), 2.00 (s, 3H, CH_3 of OAc), 1.93 (s, 3H, CH_3 of OAc) ppm. ^{13}C NMR (125 MHz, $CDCl_3$) δ 176.19 (*C*OOH), 171.49 (*C*O of OAc), 170.85 (*C*O of OAc), 169.52 (*C*O of OAc), 165.92 (NH*C*OCH_2), 146.82 (triaz-*C*), 123.44 (triaz-*C*H), 98.04 (C-1), 71.25 (C-3), 68.17 (C-4), 67.69 (C-5), 62.09 (C-6), 55.68 (O*C*H_3), 52.56 (C-2), 52.24 (NHCO*C*H_2), 33.37 (*C*H_2CO), 20.81 (2x *C*H_3 of OAc), 20.72 (triaz-*C*H_2), 20.67 (*C*H_3 of OAc) ppm. IR (ATR) 2952.36, 1738.18, 1548.62, 1366.16, 1220.66, 1031.59, 732.92, 553.38 cm^{-1}. HR-MS (+): m/z calcd for $C_{20}H_{28}N_4O_{11}$ + H^+ $[M+H]^+$ 501.1833, found 501.1783. HR-MS (+): m/z calcd for $C_{20}H_{28}N_4O_{11}$ + Na^+ $[M+Na]^+$ 523.1652, found 523.1594.

*Methyl-N-(2-deoxy-3,4,6-tri-O-acetyl-α-D-glucopyranosyl)-2-acetamido-1,2,3-triazol-4-yl-(3-oxopropyl-(oxy(2,5-dioxopyrrolidin-1-yl))) (**19**)*

18 (0.179 g, 0.357 mmol) was added to a flask with NHS (0.049 g, 0.429 mmol, 1.2 equiv.), purged with N_2 and dissolved in dry DCM. EDCI (0.082 g, 0.429 mmol, 1.2 equiv.) was dissolved in DCM and added *via* cannula. The reaction was stirred overnight at room temperature. The DCM was washed with 0.1M HCl, brine and dried with Na_2SO_4. The solvent was filtered and evaporated to yield a white solid which was used without further purification (0.176 g, 0.294 mmol, 82%). R_f = 0.64 (95:5 DCM:MeOH). ^{1}H NMR (500 MHz, $CDCl_3$) δ 7.59 (s, 1H, triaz-C*H*), 6.14 (d, *J* = 9.2 Hz, 1H, N*H*CO), 5.10 (dt, *J* = 26.2, 9.5 Hz, 2H, H-3, H-4), 4.98 (q, *J* = 16.4 Hz, 2H, NHCOCH_2), 4.71 (d, *J* = 3.7 Hz, 1H, H-1), 4.29 (ddd, *J* = 10.3, 9.3, 3.7 Hz, 1H, H-2), 4.24 (dd, *J* = 12.4, 4.7 Hz, 1H, H-6), 4.08 (dd, *J* = 12.4, 2.4 Hz, 1H, H-6'), 3.90 (ddd, *J* = 9.8, 4.6, 2.3 Hz, 1H, H-5), 3.37 (s, 3H, OCH_3), 3.21 (t, *J* = 7.1 Hz, 2H, triaz-CH_2), 3.05 (dd, *J* = 10.6, 4.1 Hz, 2H, CH_2CO), 2.84 (s, 4H, CH_2CH_2-succ), 2.09 (s, 3H, CH_3 of OAc), 2.01 (s, 3H, CH_3 of OAc), 1.98 (s, 3H, CH_3 of OAc) ppm. ^{13}C NMR (125 MHz, $CDCl_3$) δ 171.27 (*C*O of OAc), 170.82 (*C*O of OAc), 169.47 (*C*O of OAc), 169.18 (*C*O succ x2), 168.06 (*C*O), 165.36 (NH*C*OCH_2), 145.74 (triaz-*C*), 123.38 (triaz-*C*H), 97.97 (C-1), 71.25 (C-3), 68.09 (C-4), 67.72 (C-5), 62.05 (C-6), 55.70 (O*C*H_3), 52.89 (NHCO*C*H_2), 52.37 (C-2), 30.94 (*C*H_2CO), 25.72 (*C*H_2*C*H_2-succ), 21.06 (triaz-*C*H_2), 20.86 (*C*H_3 of OAc), 20.81 (*C*H_3 of OAc), 20.72 (*C*H_3 of OAc) ppm. IR (ATR) 2960.39, 1731.85, 1542.01, 1366.05, 1208.29, 1031.45, 806.41, 647.24 cm^{-1}. HR-MS (+): m/z calcd for $C_{24}H_{31}N_5O_{13}$ + H^+ $[M+H]^+$ 598.1997, found 598.2036. HR-MS (+): m/z calcd for $C_{24}H_{31}N_5O_{13}$ + Na^+ $[M+Na]^+$ 620.1816, found 620.1860.

*Methyl-N-(2-deoxy-α-D-glucopyranosyl-1,2,3-triazol-4-yl)-propanoic acid (**20**)*

18 (0.297 g, 0.593 mmol) was dissolved in MeOH (6 mL) and H_2O (3 mL). NEt_3 (0.1 mL) was added, and the reaction was stirred at 40°C overnight. The progress was monitored using TLC (90:10 DCM:MeOH). The solvent was dried, and the residue was redissolved in H_2O and stirred with amberlite H^+ resin for 1 hour. The amberlite H^+ was filtered and the filtrate was dried by lyophilisation. Yielding a fluffy white solid (Yield 0.219 g, 0.585 mmol, 98%). ^{1}H NMR (500 MHz, D_2O) δ 7.80 (s, 1H, triaz-C*H*), 5.24 (s, 2H, NHCOC*H₂*), 4.77 (d, *J* = 3.6 Hz, 1H, H-1), 3.96 (dd, *J* = 10.6, 3.6 Hz, 1H, H-2), 3.86 (dd, *J* = 12.3, 2.3 Hz, 1H, H-6), 3.74 (ddd, *J* = 16.1, 11.5, 7.3 Hz, 2H, H-3, H-6'), 3.67 (ddd, *J* = 10.0, 5.4, 2.3 Hz, 1H, H-5), 3.46 (t, *J* = 9.1 Hz, 1H, H-4), 3.38 (s, 3H, OCH_3), 2.98 (t, *J* = 7.3 Hz, 2H, triaz-CH_2), 2.66 (t, *J* = 7.3 Hz, 2H, CH_2CO) ppm. ^{13}C NMR (125 MHz, D_2O) δ 179.02 (*C*O), 168.20 (NH*C*O), 147.20 (triaz-*C*), 124.52 (triaz-*C*H), 97.90 (C-1), 71.65 (C-5), 71.14 (C-3), 69.87 (C-4), 60.50 (C-6), 55.15 (O*C*H₃), 53.81 (C-2), 51.83 (NHCO*C*H₂), 34.53 (*C*H₂CO), 20.73 (triaz-*C*H₂) ppm. IR (ATR) 3287.05, 1664.63, 1562.97, 1562.97, 1114.43, 1050.55, 1030.28 cm^{-1}. HR-MS (+): m/z calcd for $C_{14}H_{22}N_4O_8$ + H^+ $[M+H]^+$ 375.1516, found 375.1510. HR-MS (+): m/z calcd for $C_{14}H_{22}N_4O_8$ + Na^+ $[M+Na]^+$ 397.1335, found 397.1327.

*Methyl-N-(2-deoxy-α-D-glucopyranosyl-1,2,3-triazol-4-yl)-(3-oxopropyl-(oxy(2,5-dioxopyrrolidin-1-yl))) (**21**)*

20 (0.05g, 0.1335 mmol) and TSTU (0.0.44 g, 0.1469 mmol, 1.1 equiv.) were added to a flask, flushed with N_2 and dissolved in DMF (7 mL). NEt_3 (0.02 mL, 0.1469 mmol, 1.1 equiv.) was added to the solution and the reaction was allowed to stir at room temperature for 20 minutes. Checking reaction progress by TLC (60:35:5 DCM:MeOH:H_2O). The DMF was evaporated, and the residue was washed with DCM, collecting the precipitating product by centrifugation. The pink/white solid was washed with diethyl ether and dried *in vacuo* (Yield 0.049 g, 0.103 mmol, 79%). R_f = 0.83 (DCM:MeOH:H_2O 60:35:5). ^{1}H NMR (500 MHz, DMSO) δ 8.33 (d, *J* = 8.4 Hz, 1H, N*H*CO), 7.86 (s, 1H, triaz-C*H*), 5.13 – 5.05 (m, 2H, NHCOC*H₂*), 5.04 (d, *J* = 5.7 Hz, 1H, O*H* of C-4), 4.90 (d, *J* = 5.6 Hz, 1H, O*H* of C-3), 4.56 (d, *J* = 3.5 Hz, 1H, H-1), 4.53 (t, *J* = 5.9 Hz, 1H, O*H* of C-6), 3.71 – 3.62 (m, 2H, H-2, H-6), 3.51 – 3.45 (m, 2H, H-6', H-3), 3.37 – 3.34 (m, 1H, overlaps with H_2O, H-5), 3.27 (s, 3H, OCH_3), 3.18 – 3.12 (m, 1H, H-4), 3.08 – 3.04 (m, 2H, *C*H₂CO), 2.99 (dd, *J* = 11.3, 4.2 Hz, 2H, triaz-CH_2), 2.81 (s, 4H, CH_2CH_2-succ) ppm. ^{13}C NMR (125 MHz, DMSO) δ 170.22 (*C*O succ x2), 168.36 (*C*O), 165.58 (*C*ONH), 144.10 (triaz-*C*), 123.85 (triaz-*C*H), 97.74 (C-1), 72.80 (C-5), 70.83 (C-3), 70.65 (C-4), 60.75 (C-6), 54.33 (O*C*H₃), 54.04 (C-2), 51.49 (NHCO*C*H₂), 29.80 (*C*H₂CO), 25.46 (*C*H₂CH₂-Succ), 20.27 (triaz-*C*H₂) ppm. IR (ATR) 3285.14, 1732.92, 1667.17, 1559.92, 1208.18, 1024.06, 646.62 cm^{-1}. HR-MS (+): m/z calcd for $C_{18}H_{25}N_5O_{10}$ + H^+ $[M+H]^+$ 472.1680, found 472.1696. HR-MS (+): m/z calcd for $C_{18}H_{25}N_5O_{10}$ + Na^+ $[M+Na]^+$ 494.1499, found 494.1534.

*Cis,cis,trans-[$Pt_{IV}(NH_3)_2$(**7**)(OH)Cl_2] (**1**)*

8 (0.130 g, 0.2286 mmol) was added to a round bottom flask containing a suspension of oxoplatin (0.080 g, 0.239 mmol) in DMSO (6 mL) and stirred overnight at 60°C in the dark. The next morning, excess oxoplatin was filtered through cotton and the DMSO was evaporated by lyophilization. The oily residue was redissolved in acetone and the product was precipitated using diethyl ether. The residual solvent was then removed *in vacuo* yielding a yellow solid (0.122 g, 0.154 mmol, 67%). (The ratio of alpha:beta was 1:0.51). ^{1}H NMR (500 MHz, DMSO) δ 8.01 (s, 1H, triaz-C*H*β), 7.92 (s, 0.54H, triaz-C*H*α), 6.35 (d, *J* = 8.7 Hz, 1H, H-1β), 6.26 (d, *J* = 3.5 Hz, 0.51H, H-1α), 6.14 – 5.78 (m, 10.5H, α/β NH_3, H-3β, H-3α), 5.36 (dd, *J* = 11.2, 3.5 Hz, 0.54H, H-2α), 5.19 (t, *J* = 9.85, 0.60H, H-4α), 5.09 (t, *J* = 9.7 Hz, 1H, H-4β), 4.90 (dd, *J* = 10.7, 8.7 Hz, 1H, H-2β), 4.35 (ddd, *J* = 10.1, 4.6, 2.3 Hz, 1H, H-5β), 4.32 – 4.27 (m, 0.64H, H-5α), 4.24 (m, 1.74H, H-6α, H-6β), 4.10 – 4.02 (m, 1.77H, H-6'α, H-6'β), 2.78 (t, *J* = 7.8, 3H, α/β triaz-CH_2), 2.45 – 2.39 (m, 3H, α/β CH_2CO), 2.10 (s, 1.5H, α CH_3 of OAc), 2.03 (d, *J* = 1.6 Hz, 4.5H, α/β CH_3 of OAc), 2.00 (s, 1.5H, α CH_3 of OAc), 1.99 (s, 3H, β CH_3 of OAc), 1.93 (s, 3H, β CH_3 of OAc), 1.80 (s, 3H, β CH_3 of OAc), 1.79 (s, 3H, α CH_3 of OAc) ppm. ^{13}C NMR (125 MHz, DMSO) δ 179.73 (*C*OOPt α), 179.70 (*C*OOPt β), 170.05 (2x *C*O of OAc), 169.35 (*C*O of OAc), 169.28 (*C*O of OAc), 169.22 (*C*O of OAc), 168.91 (*C*O of OAc), 168.51 (*C*O of OAc), 168.29 (*C*O of OAc), 146.66 (triaz-*C*α), 146.56 (triaz-*C*β), 122.11 (triaz-*C*Hβ), 121.92 (triaz-*C*Hα), 91.03 (C-1β), 89.54 (C-1α), 71.70 (C-5β), 71.50 (C-3β), 69.33 (C-

5α), 68.82 (C-3α), 68.00 (C-4α), 67.90 (C-4β), 61.65 (C-2β), 61.44 (C-6β), 61.22 (C-6α), 59.66 (C-2α), 36.17 (α *C*H_2CO), 36.09 (β *C*H_2CO), 21.85 (α/β triaz-*C*H_2), 20.55 (3x *C*H_3 of OAc), 20.39 (*C*H_3 of OAc), 20.37 (*C*H_3 of OAc) 20.28 (*C*H_3 of OAc), 20.11 (*C*H_3 of OAc), 20.02 (*C*H_3 of OAc) ppm. ^{195}Pt{^{1}H} NMR (108 MHz, DMSO) δ 1045.54 ppm. IR (ATR) 3220.55, 1747.64, 1623.98, 1367.12, 1212.66, 1043.50, 906.86 cm^{-1}. Elem. Anal. Calcd (%) for $C_{19}H_{31}Cl_2N_5O_{12}Pt$: C: 28.98, H: 3.97, N: 8.89; found C: 29.36, H: 4.33, N: 9.28. HR-MS (-): m/z calcd for $C_{19}H_{31}Cl_2N_5O_{12}Pt$ – H^- $[M-H]^-$ 786.4560, found 786.0913.

*Cis,cis,trans-[Pt^{IV}(NH$_3$)$_2$(**14**)(OH)Cl$_2$] (**2**)*

15 (0.236 g, 0.436 mmol) was added to a suspension of oxoplatin (0.153 g, 0.458 mmol, 1.05 equiv.) in DMSO (15 mL) and stirred at 55°C overnight. The resulting suspension was filtered, and the filtrate was lyophilized to remove the DMSO. The oily residue was washed then dissolved in acetone and precipitated with diethyl ether to yield a pale yellow powder (0.285 g, 0.375 mmol, 86%). ^{1}H NMR (500 MHz, DMSO) δ 7.81 (s, 1H, triaz-C*H*), 6.13 – 5.81 (br t, *J* = 51.65 Hz, 6H, 2x NH_3), 5.73 – 5.67 (m, 1H, H-3), 5.14 – 5.06 (m, 3H, H-4, H-1, H-2), 4.22 (dd, *J* = 12.3, 5.0 Hz, 1H, H-6), 4.11 (dd, *J* = 12.3, 2.4 Hz, 1H, H-6'), 4.06 (ddd, *J* = 10.2, 4.9, 2.4 Hz, 1H, H-5), 3.32 (s, 3H, OC*H$_3$* overlaps with H_2O), 2.81 – 2.77 (m, 2H, triaz-C*H$_2$*), 2.46 – 2.42 (m, 2H, C*H$_2$*CO), 2.04 (s, 3H, C*H$_3$* of OAc), 2.00 (s, 3H, C*H$_3$* of OAc), 1.75 (s, 3H, C*H$_3$* of OAc) ppm. ^{13}C NMR (125 MHz, DMSO) δ 179.72 (*C*O), 170.08 (*C*O of OAc), 169.31 (*C*O of OAc), 169.22 (*C*O of OAc), 146.46 (triaz-*C*), 121.50 (triaz-*C*H), 97.12 (C-1), 69.08 (C-3), 68.52 (C-4), 67.07 (C-5), 61.75 (C-6), 60.86 (C-2), 55.01 (O*C*H_3), 36.13 (*C*H_2CO), 21.85 (triaz-*C*H_2), 20.53 (*C*H_3 of OAc), 20.38 (*C*H_3 of OAc), 20.06 (*C*H_3 of OAc) ppm. ^{195}Pt{^{1}H} NMR (108 MHz, DMSO) δ 1046.86 ppm. IR (ATR) 3217.18, 1744.63, 1624.51, 1367.45, 1224.71, 1040.54, 928.27 cm^{-1}. Elem. Anal. Calcd (%) for $C_{18}H_{31}Cl_2N_5O_{11}Pt$: C: 28.47, H: 4.11, N: 9.22; found C: 28.86, H: 4.45, N: 9.48. HR-MS (+): m/z calcd for $C_{18}H_{31}Cl_2N_5O_{11}Pt$ + H^+ $[M+H]^+$ 759.1123, found 759.1141. HR-MS (+): m/z calcd for $C_{18}H_{31}Cl_2N_5O_{11}Pt$ + Na^+ $[M+Na]^+$ 781.0943, found 781.0964.

*Cis,cis,trans-[Pt^{IV}(NH$_3$)$_2$(**18**)(OH)Cl$_2$] (**3**)*

19 (0.176 g, 0.294 mmol) was added to a suspension of oxoplatin (0.103 g, 0.309 mmol, 1.05 equiv.) in DMSO (10 mL) and stirred overnight at 60°C in the dark. The excess oxoplatin was filtered through cotton and the DMSO was lyophilized. The residue was redissolved in acetone and the product was precipitated with diethyl ether and dried with the Schlenk line to yield a pale yellow solid (0.197 g, 82%). ^{1}H NMR (500 MHz, DMSO) δ 8.52 (d, *J* = 9.2 Hz, 1H, N*H*CO), 7.80 (s, 1H, triaz-C*H*), 5.97 (br t, *J* = 49.9 Hz, 6H), 5.15 (dd, *J* = 10.9, 9.4 Hz, 1H, H-3), 5.07 – 4.98 (m, 2H, NHCOC*H$_2$*), 4.90 (t, *J* = 9.8 Hz, 1H, H-4), 4.75 (d, *J* = 3.4 Hz, 1H, H-1), 4.17 (dd, *J* = 12.4, 4.9 Hz, 1H, H-6), 4.14 – 4.09 (m, 1H, H-2), 4.04 (dd, *J* = 12.3, 2.3 Hz, 1H, H-6'), 3.93 (ddd, *J* = 10.1, 4.8, 2.4 Hz, 1H, H-5), 3.37 (s, 3H, OC*H$_3$*), 2.81 (t, *J* = 7.5 Hz, 2H, triaz-C*H$_2$*), 2.46 (t, *J* = 7.6 Hz, 2H, C*H$_2$*CO), 2.02 (s, 3H, C*H$_3$* of OAc), 1.97 (s, 3H, C*H$_3$* of OAc), 1.89 (s, 3H, C*H$_3$* of OAc) ppm. ^{13}C NMR (125 MHz, DMSO) δ 179.89 (COOPt), 170.16 (*C*O of OAc), 169.89 (*C*O of OAc), 169.34 (*C*O of OAc), 166.15 (NH*C*OCH_2), 146.11 (triaz-*C*), 123.79 (triaz-*C*H), 97.62 (C-1), 70.35 (C-3), 68.55 (C-4), 66.97 (C-5), 61.87 (C-6), 55.02 (O*C*H_3), 51.25 (NHCO*C*H_2), 51.09 (C-2), 36.14 (*C*H_2CO), 21.92 (triaz-*C*H_2), 20.55 (*C*H_3 of OAc), 20.44 (2x *C*H_3 of OAc) ppm. ^{195}Pt{^{1}H} NMR (108 MHz, DMSO) δ 1046.19 ppm. IR (ATR) 3231.16, 1743.14, 1675.08, 1558.18, 1366.47, 1226.87, 1034.87 cm^{-1}. Elem. Anal. Calcd (%) for $C_{20}H_{34}Cl_2N_6O_{12}Pt$: C: 29.42, H: 4.20, N: 10.29; found C: 29.86, H: 4.49, N: 9.95. HR-MS (+): m/z calcd for $C_{20}H_{34}Cl_2N_6O_{12}Pt$ + H^+ $[M+H]^+$ 817.5140, found 817.1320. HR-MS (+): m/z calcd for $C_{20}H_{34}Cl_2N_6O_{12}Pt$ + Na^+ $[M+Na]^+$ 839.4958, found 839.1130.

*Cis,cis,trans-[Pt^{IV}(NH$_3$)$_2$(**20**)(OH)Cl$_2$] (**4**)*

21 (0.075 g, 0.159 mmol) dissolved in dry DMSO (5 mL) was added dropwise, over 24 hr, to a suspension of oxoplatin (0.106 g, 0.318 mmol, 2 equiv.) in dry DMSO (7 mL) at 40°C. The reaction flask was heated at 40°C for a further 5 days before filtration of excess oxoplatin and removal of solvent via lyophilisation. The resulting oil was rinsed with the minimum amount of MeOH, washed with diethyl ether and dried in vacuo to yield the product as a pale yellow powder (0.043g, 0.062 mmol, 39%). ^{1}H NMR (500 MHz, DMSO) δ 8.34 (d, *J* = 8.5 Hz, 1H, N*H*CO), 7.82 (s, 1H, triaz-C*H*), 5.97 (t, 6H, 2x NH_3),

5.11 – 5.00 (m, 3H, NHCOC*H*$_2$, O*H* of C-4), 4.94 (d, *J* = 5.2 Hz, 1H, O*H* of C-3), 4.63 – 4.49 (m, 2H, H-1, O*H* of C-6), 3.72 – 3.62 (m, 2H, H-2, H-6), 3.51 – 3.32 (m, 3H, H-6', H-3, H-5), 3.27 (s, 3H, OC*H*$_3$), 3.16 – 3.11 (m, 1H, H-4), 2.82 (t, *J* = 7.5 Hz, 2H, triaz-C*H*$_2$), 2.47 (t, *J* = 7.6 Hz, 2H, C*H*$_2$CO) ppm. ^{13}C NMR (125 MHz, DMSO) δ 179.98 (*C*OOPt), 165.77 (*C*ONH), 146.13 (triaz-*C*), 123.83 (triaz-*C*H), 97.79 (C-1), 72.81 (C-5), 70.89 (C-3), 70.68 (C-4), 60.80 (C-6), 54.39 (O*C*H$_3$), 54.06 (C-2), 51.47 (NHCO*C*H$_2$), 36.20 (*C*H$_2$CO), 21.96 (triaz-*C*H$_2$) ppm. ^{195}Pt{^{1}H} NMR (108 MHz, DMSO) δ 1044.47 ppm. IR (ATR) 3256.68, 1676.10, 1559.39, 1021.64, 992.54 cm^{-1}. Elem. Anal. Calcd (%) for $C_{14}H_{28}Cl_2N_6O_9Pt$: C: 24.36, H: 4.09, N: 12.17; found C: 24.76, H: 4.38, N: 12.48. HR-MS (+): m/z calcd for $C_{14}H_{28}Cl_2N_6O_9Pt$ + H^+ $[M+H]^+$ 690.1021, found 690.1102.

NMR Characterisation

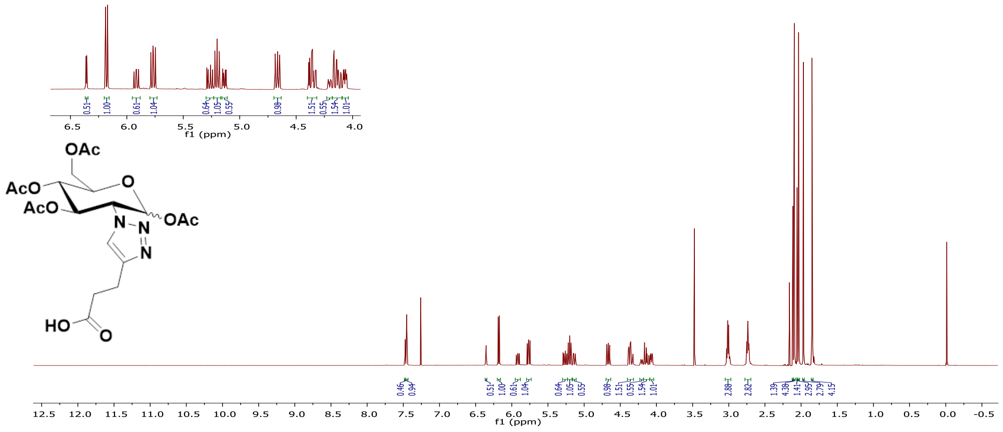

^{1}H NMR spectrum of **7** in $CDCl_3$

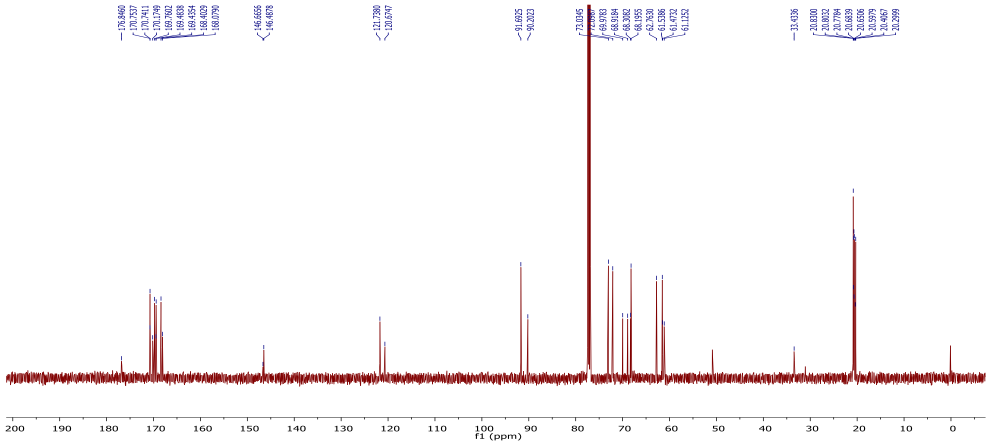

^{13}C NMR spectrum of **7** in $CDCl_3$

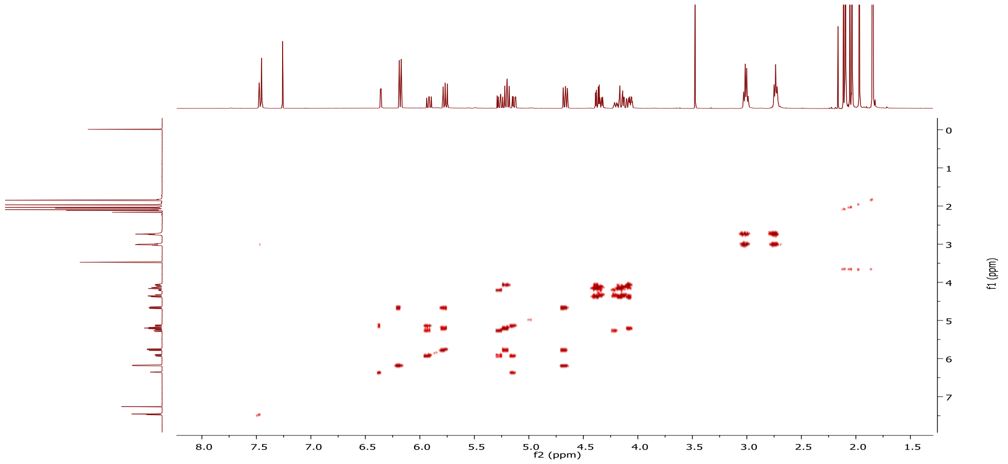

COSY NMR spectrum of **7** in $CDCl_3$

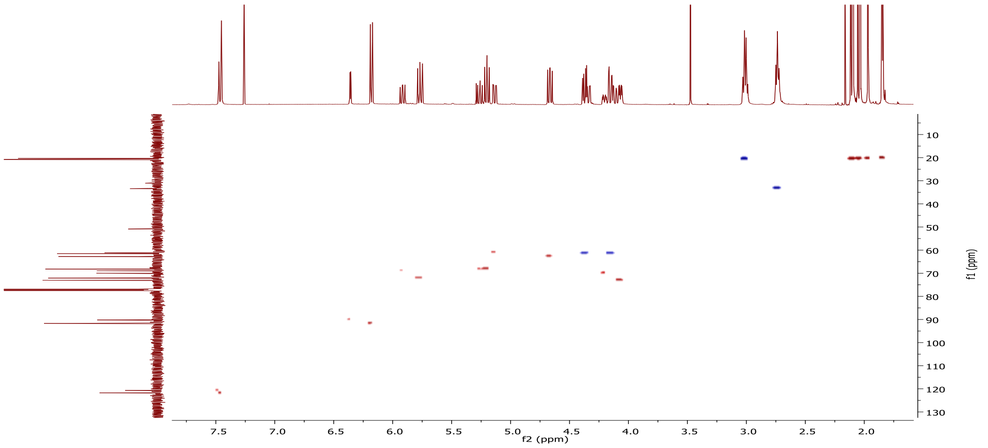

HSQC NMR spectrum of **7** in $CDCl_3$

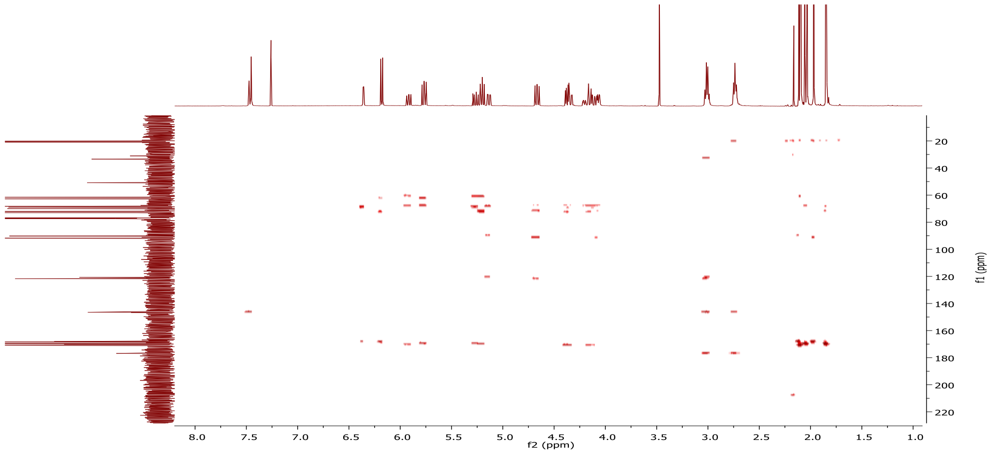

HMBC NMR spectrum of **7** in $CDCl_3$

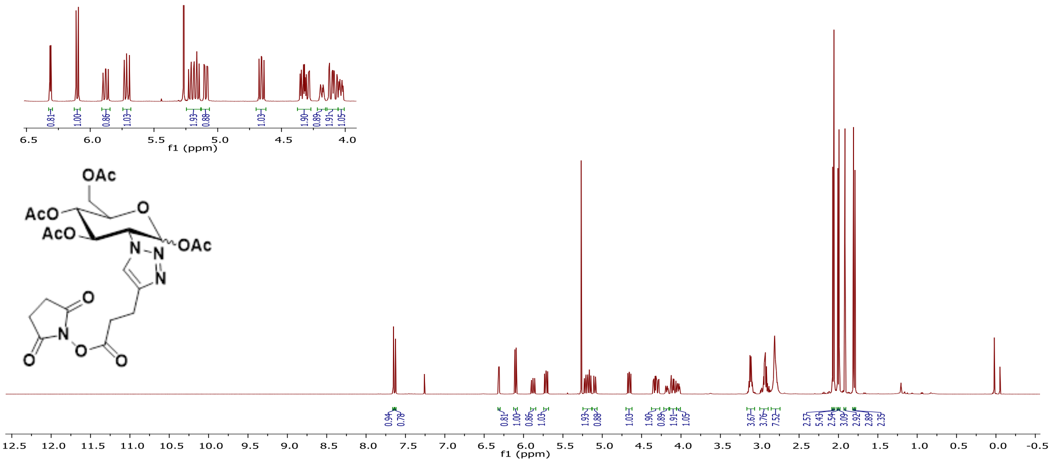

^{1}H NMR spectrum of **8** in $CDCl_3$

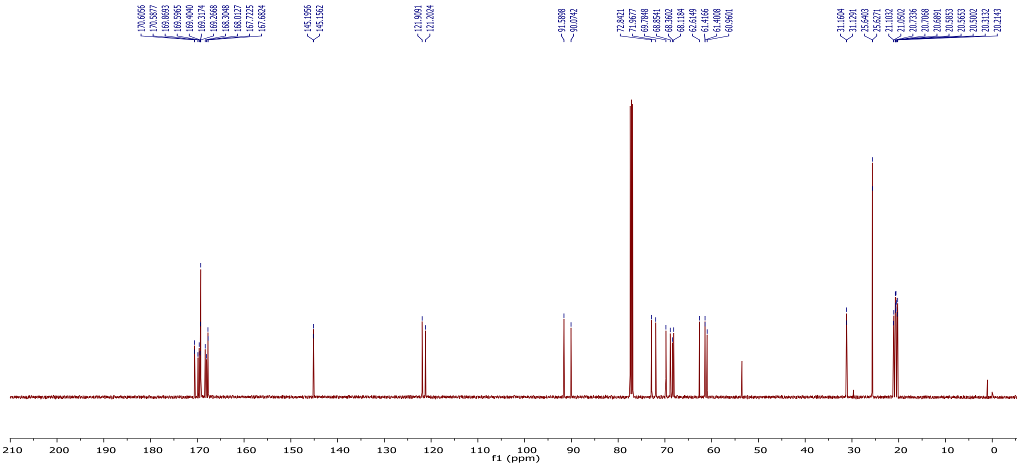

^{13}C NMR spectrum of **8** in $CDCl_3$

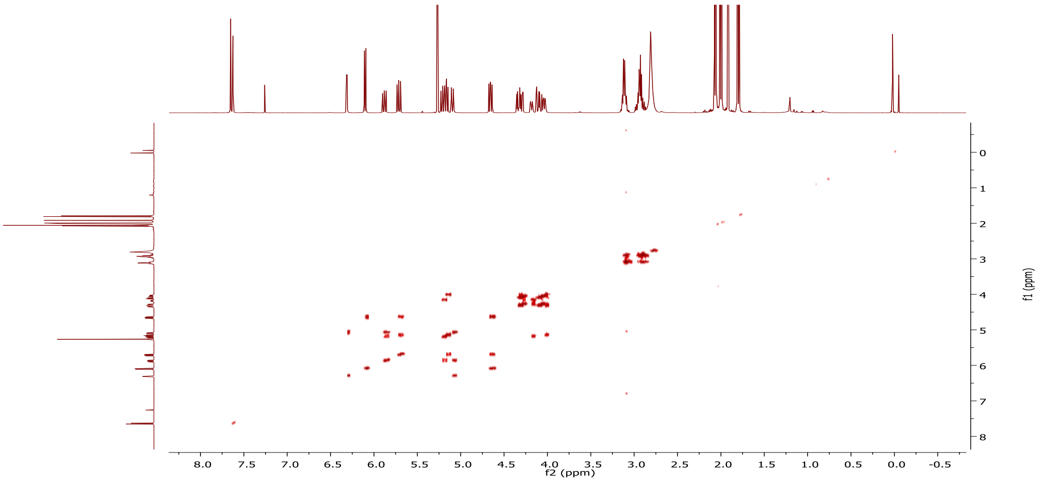

COSY NMR spectrum of **8** in $CDCl_3$

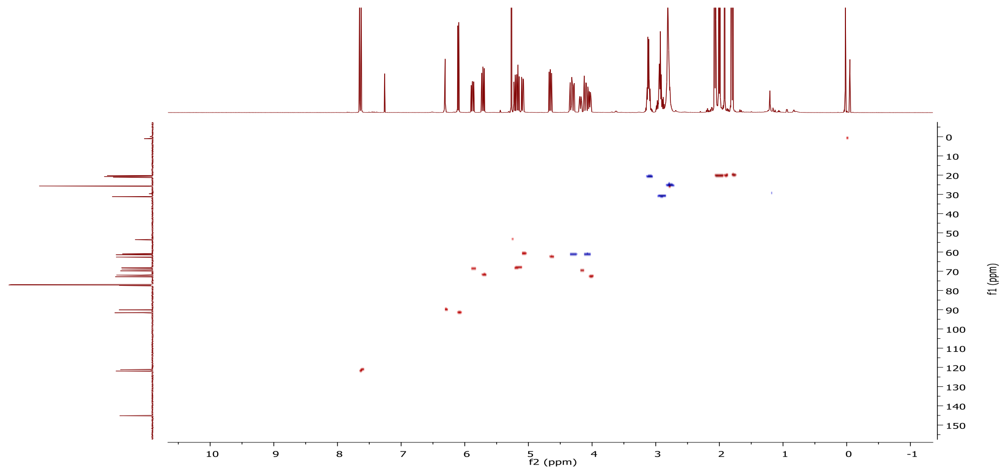

HSQC NMR spectrum of **8** in $CDCl_3$

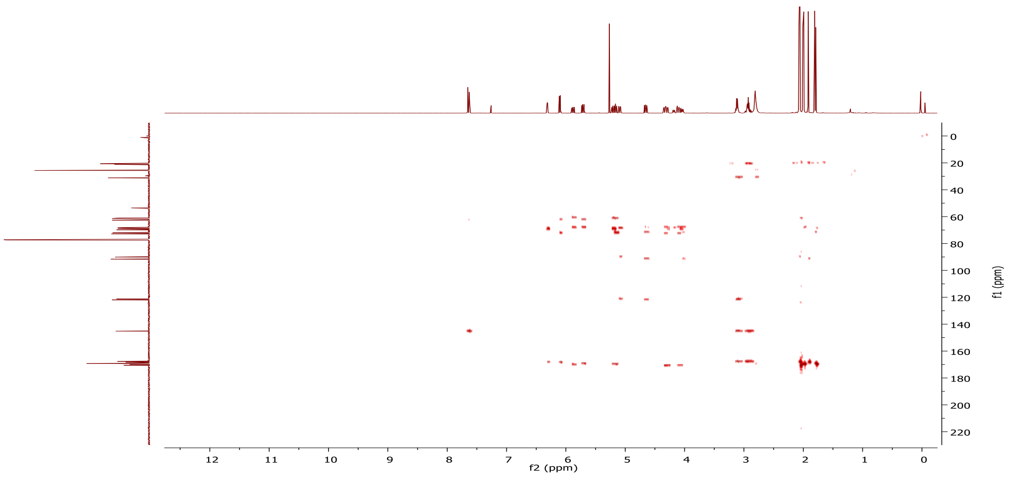

HMBC NMR spectrum of **8** in $CDCl_3$

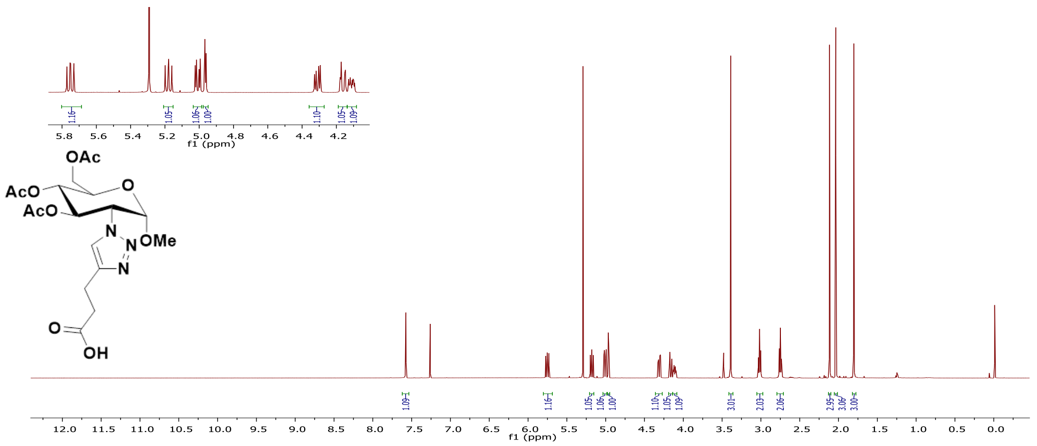

^1H NMR spectrum of **14** in $CDCl_3$

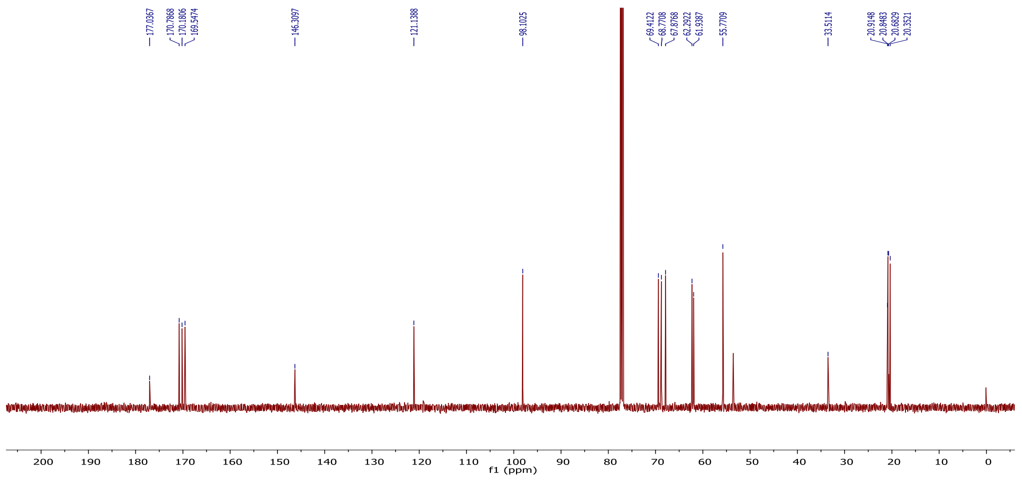

^{13}C NMR spectrum of **14** in $CDCl_3$

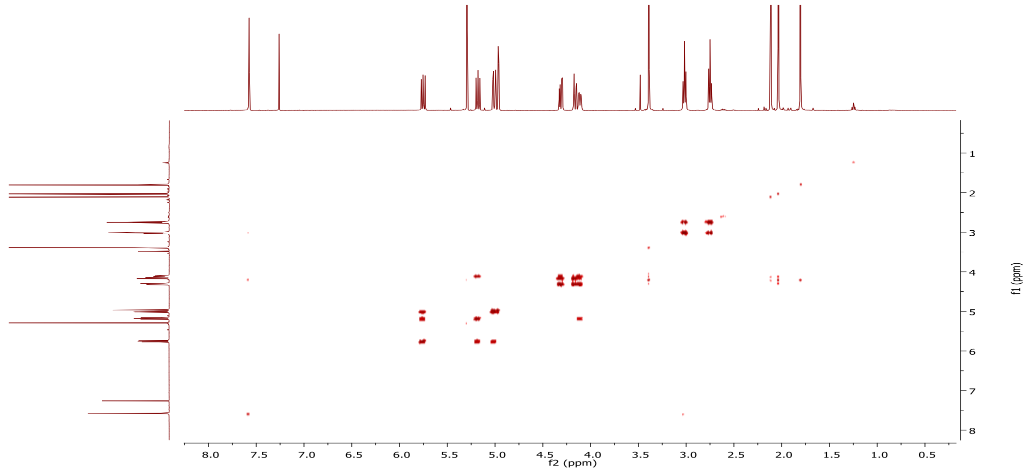

COSY NMR spectrum of **14** in $CDCl_3$

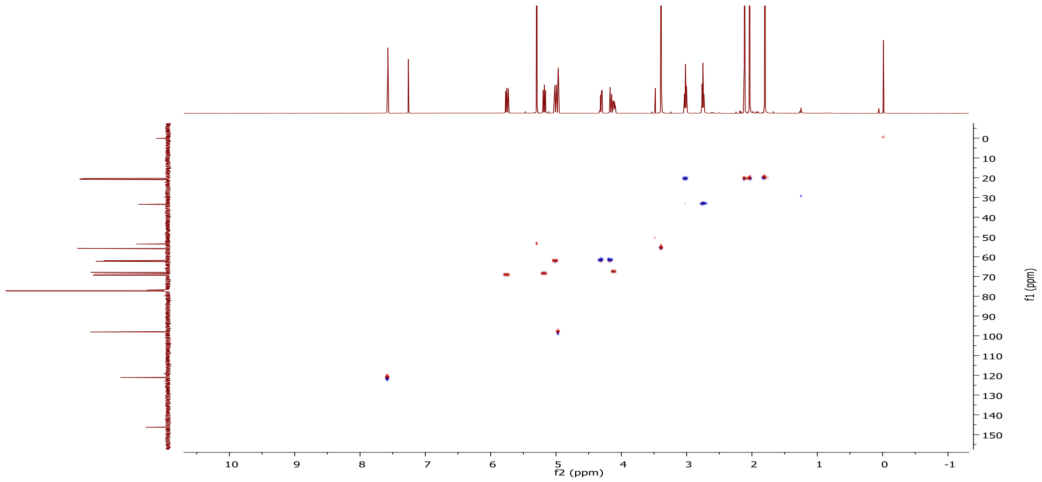

HSQC NMR spectrum of **14** in $CDCl_3$

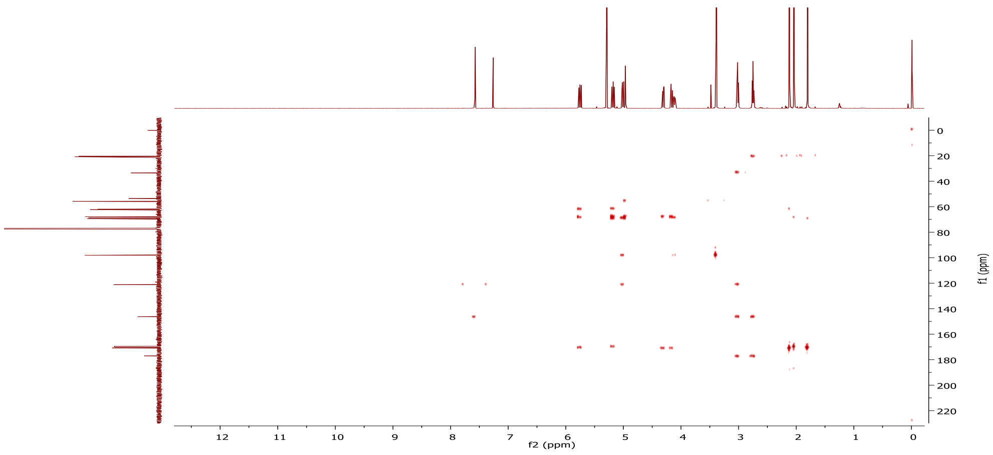

HMBC NMR spectrum of **14** in $CDCl_3$

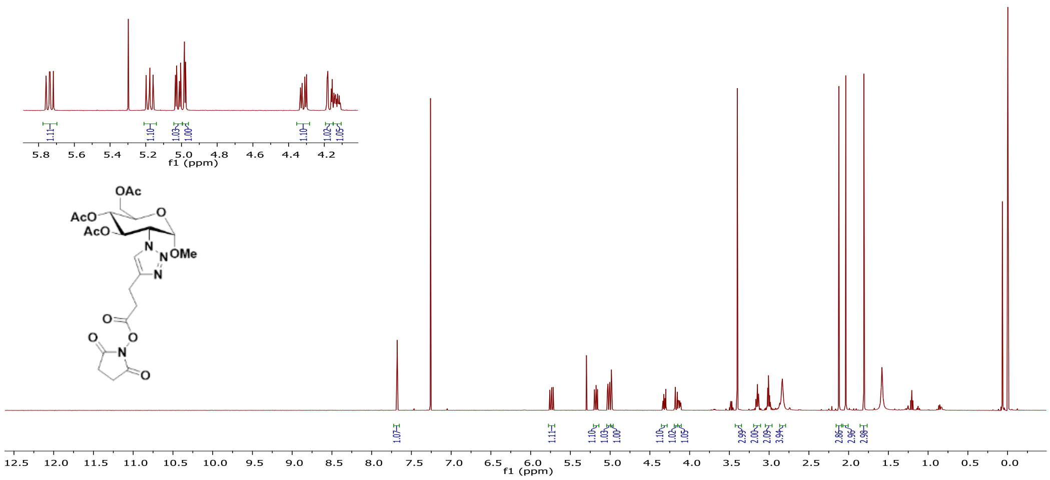

^{1}H NMR spectrum of **15** in $CDCl_3$

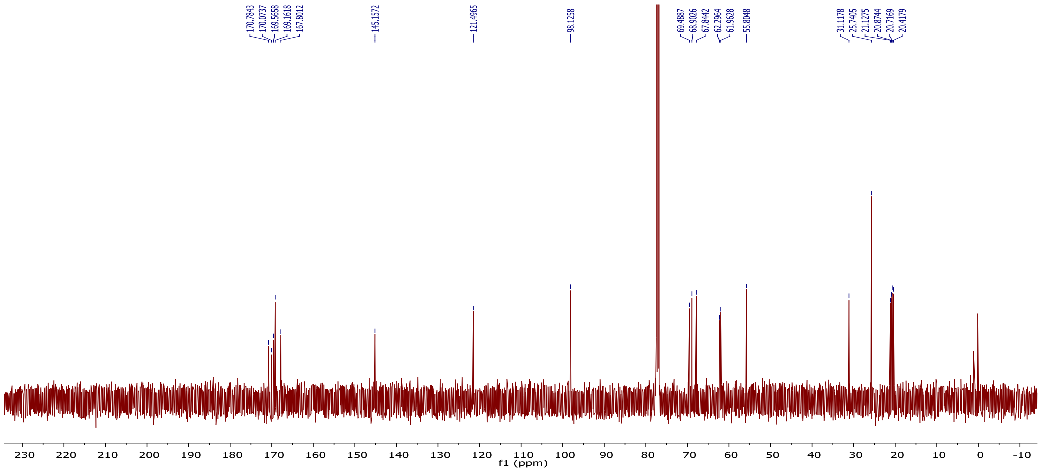

^{13}C NMR spectrum of **15** in $CDCl_3$

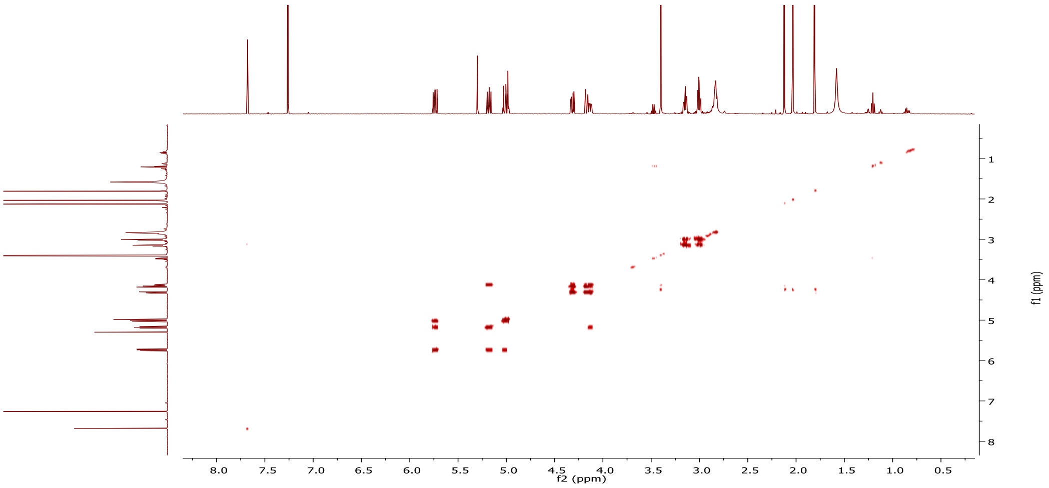

COSY NMR spectrum of **15** in $CDCl_3$

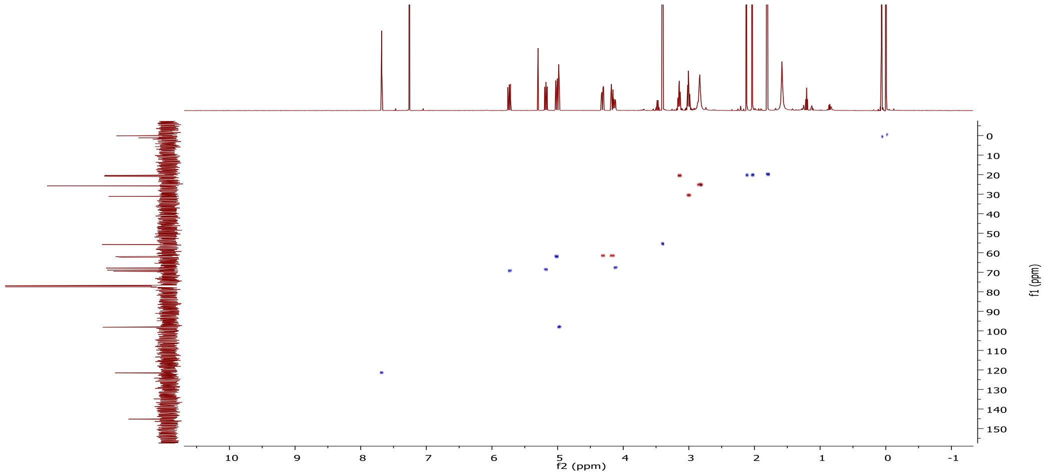

HSQC NMR spectrum of **15** in $CDCl_3$

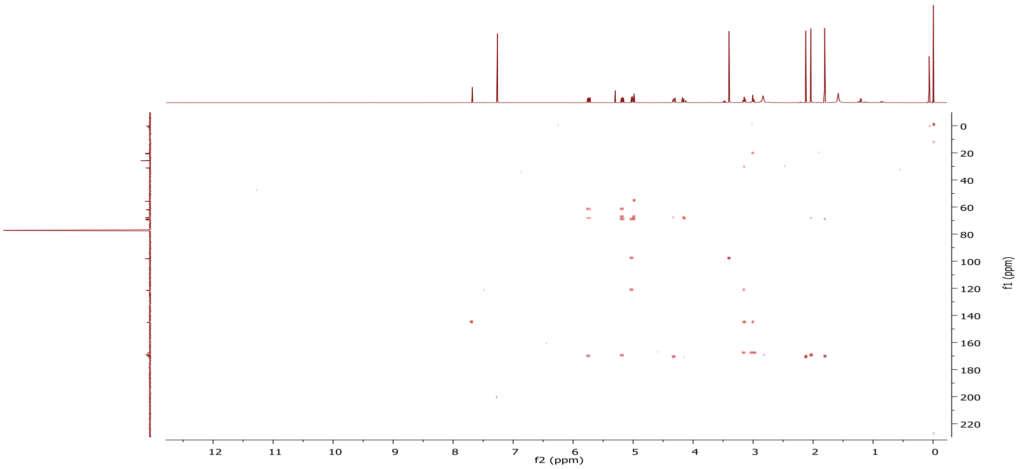

HMBC NMR spectrum of **15** in $CDCl_3$

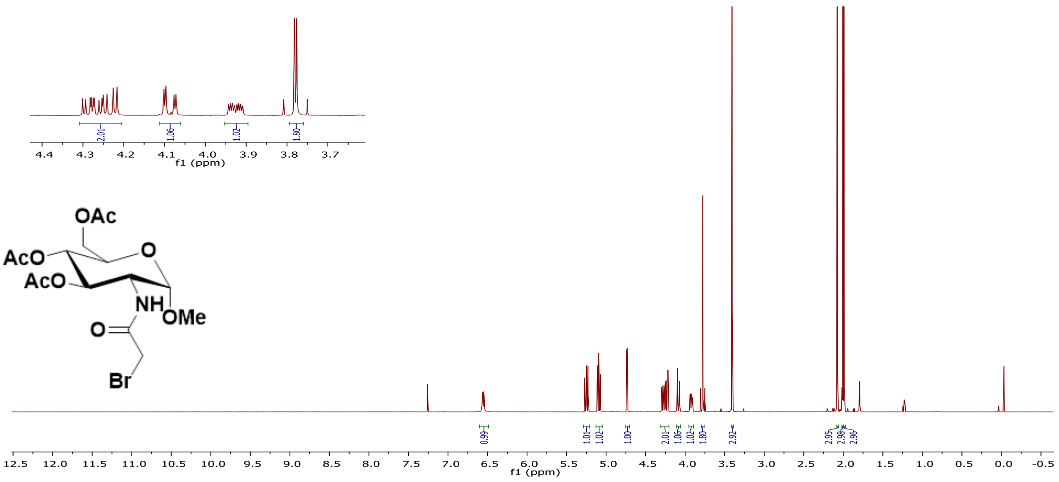

^{1}H NMR spectrum of **16** in $CDCl_3$

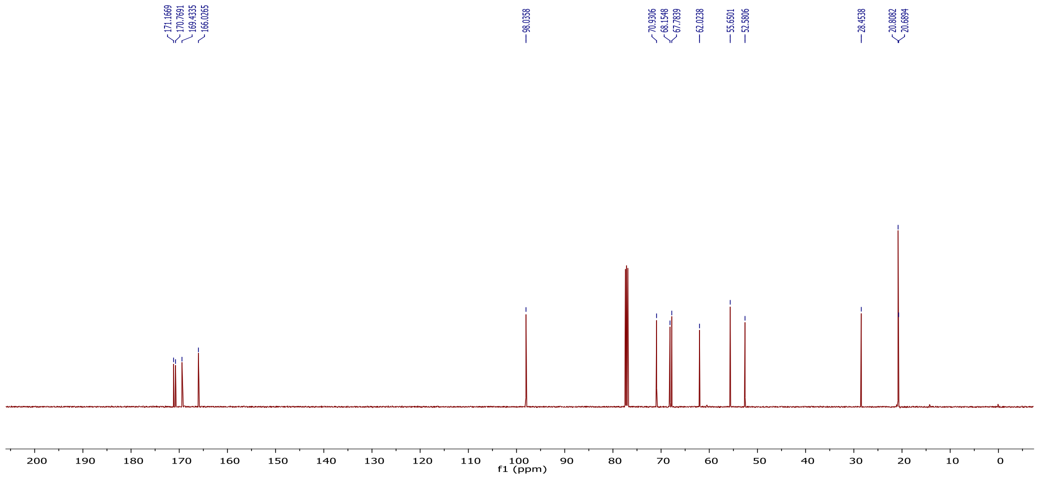

^{13}C NMR spectrum of **16** in $CDCl_3$

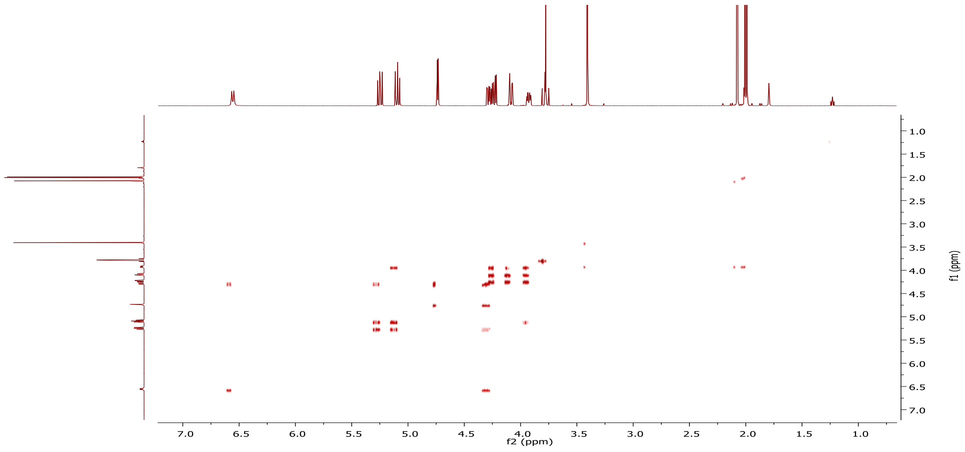

COSY NMR spectrum of **16** in $CDCl_3$

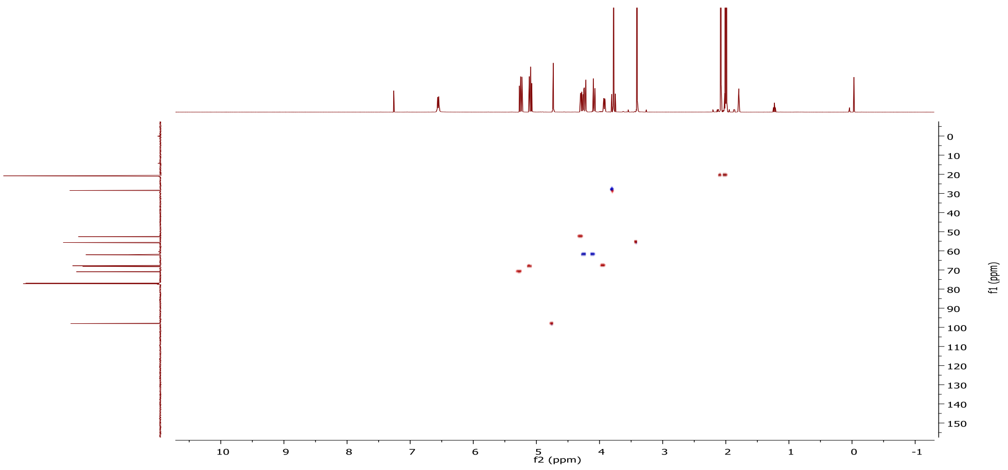

HSQC NMR spectrum of **16** in $CDCl_3$

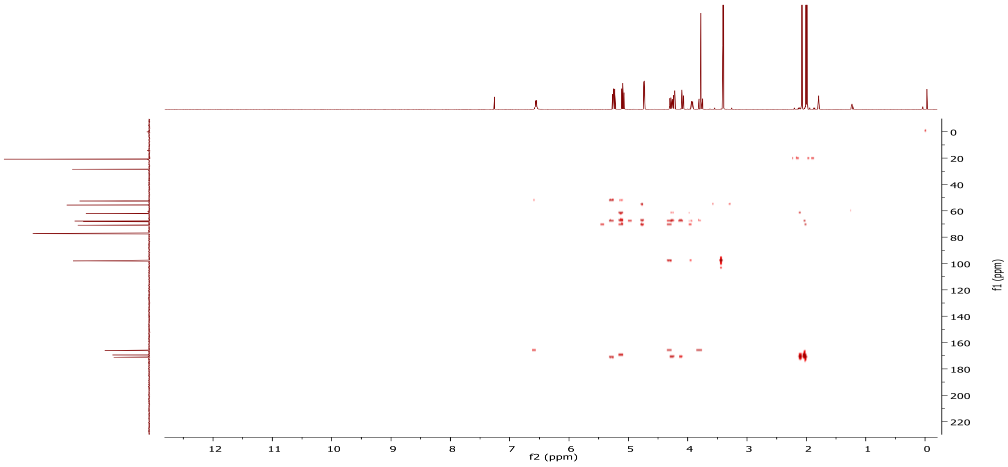

HMBC NMR spectrum of **16** in $CDCl_3$

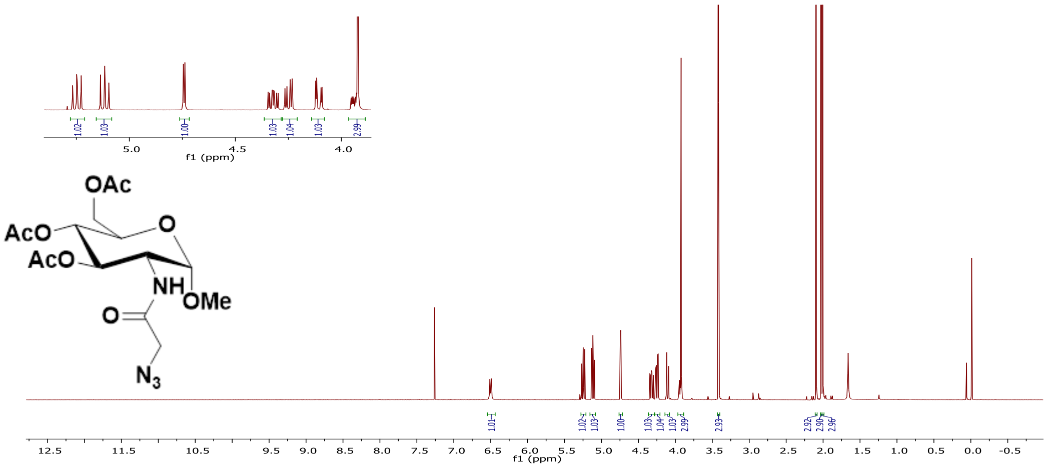

^{1}H NMR spectrum of **17** in $CDCl_3$

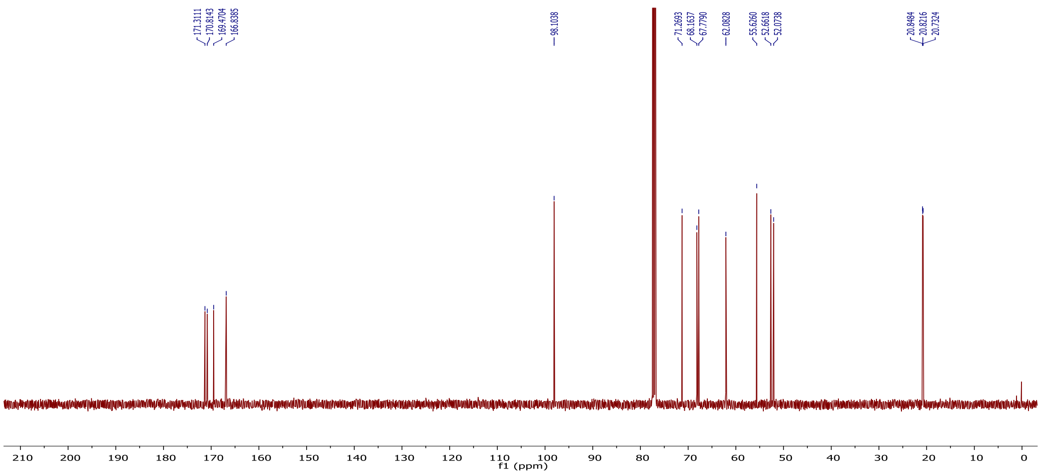

^{13}C NMR spectrum of **17** in $CDCl_3$

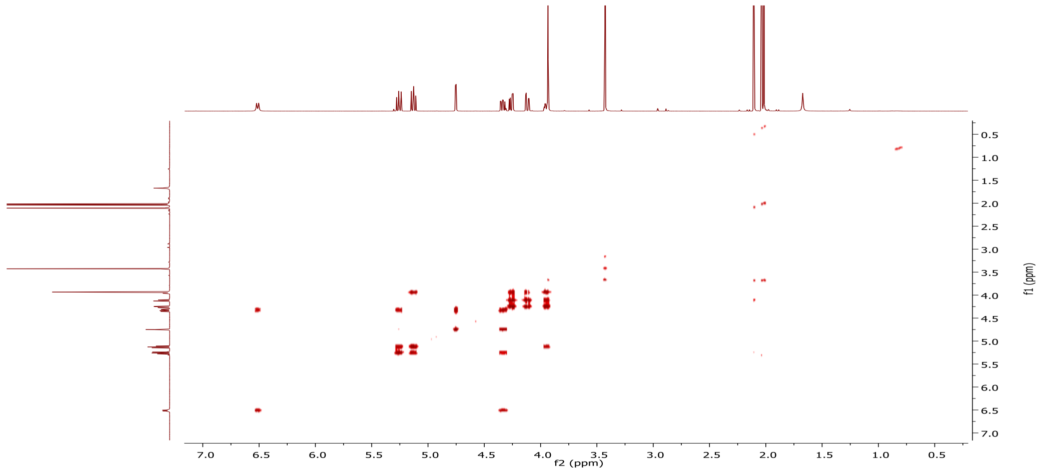

COSY NMR spectrum of **17** in $CDCl_3$

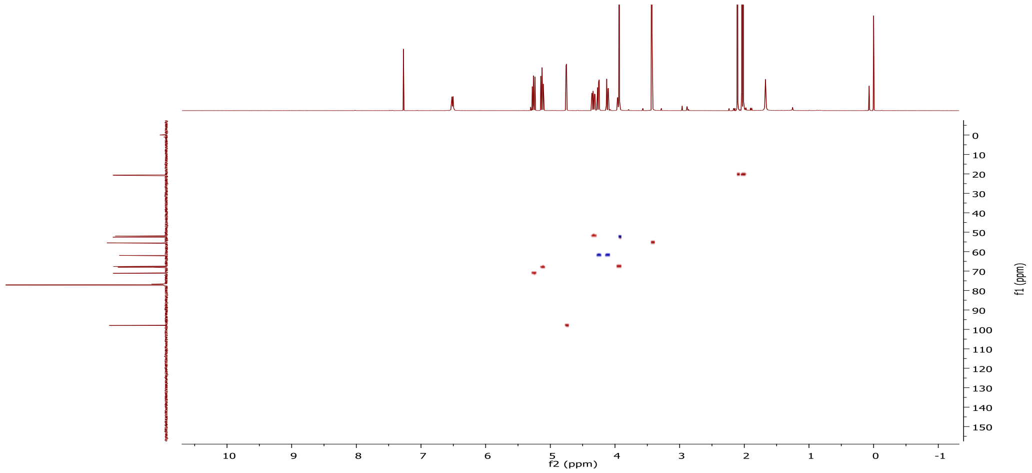

HSQC NMR spectrum of **17** in $CDCl_3$

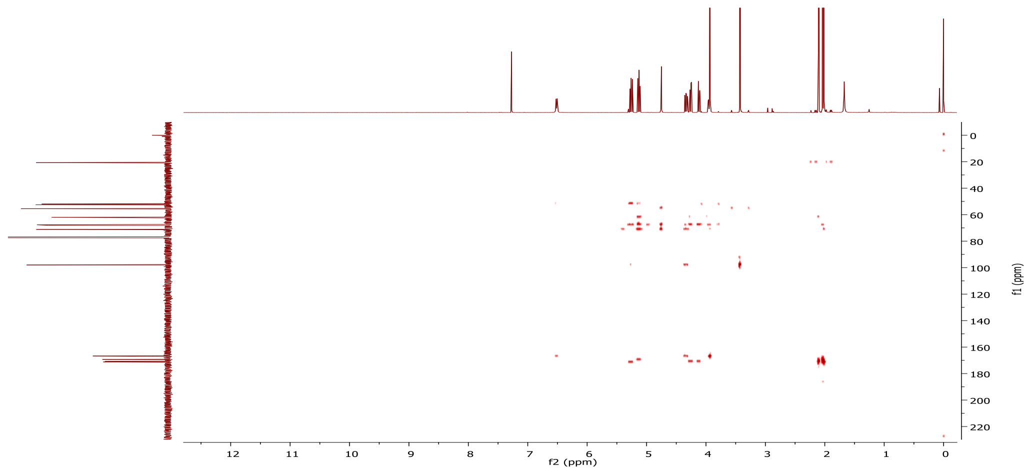

HMBC NMR spectrum of **17** in $CDCl_3$

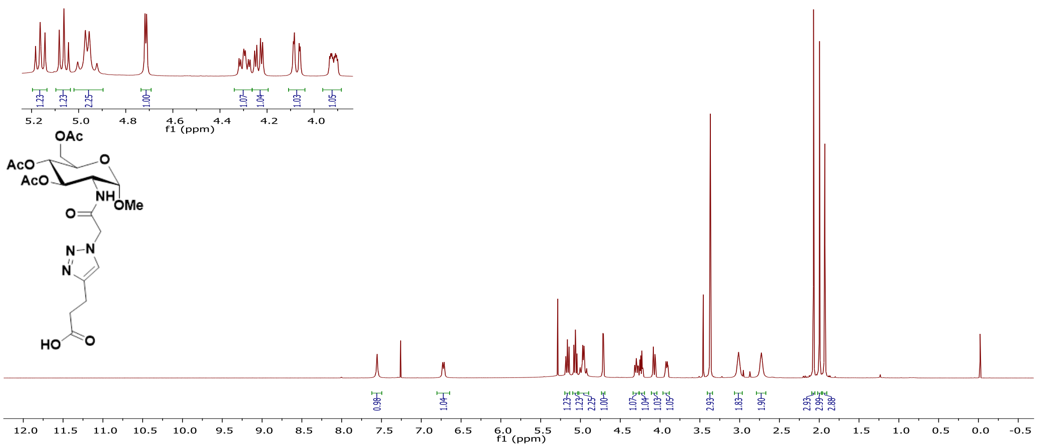

^{1}H NMR spectrum of **18** in $CDCl_3$

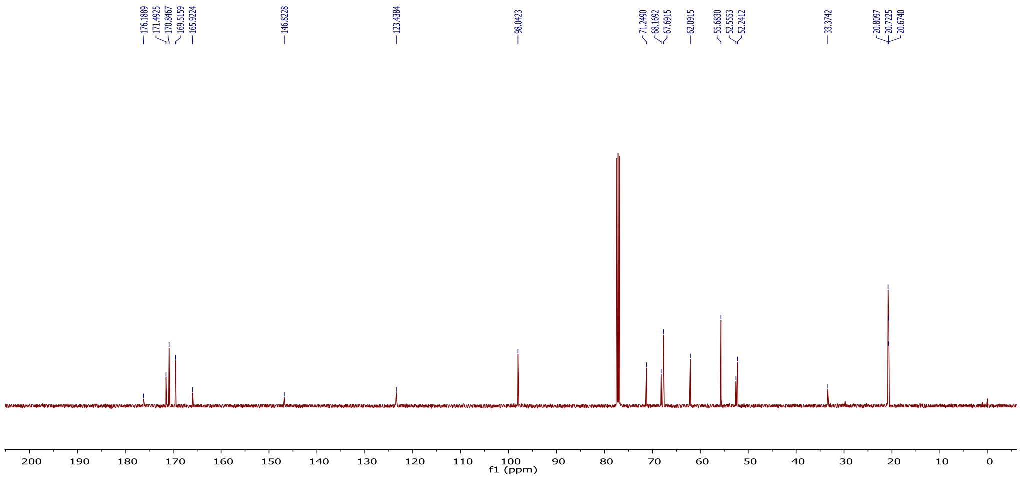

^{13}C NMR spectrum of **18** in $CDCl_3$

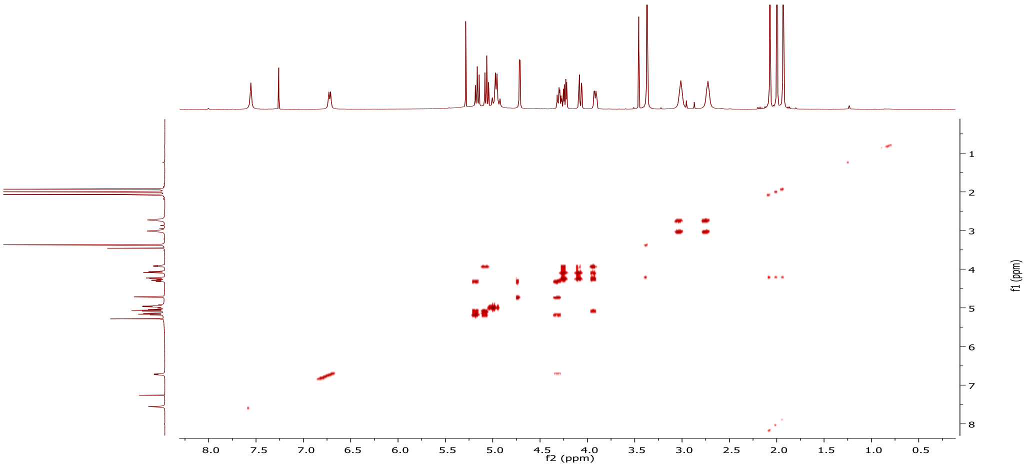

COSY NMR spectrum of **18** in $CDCl_3$

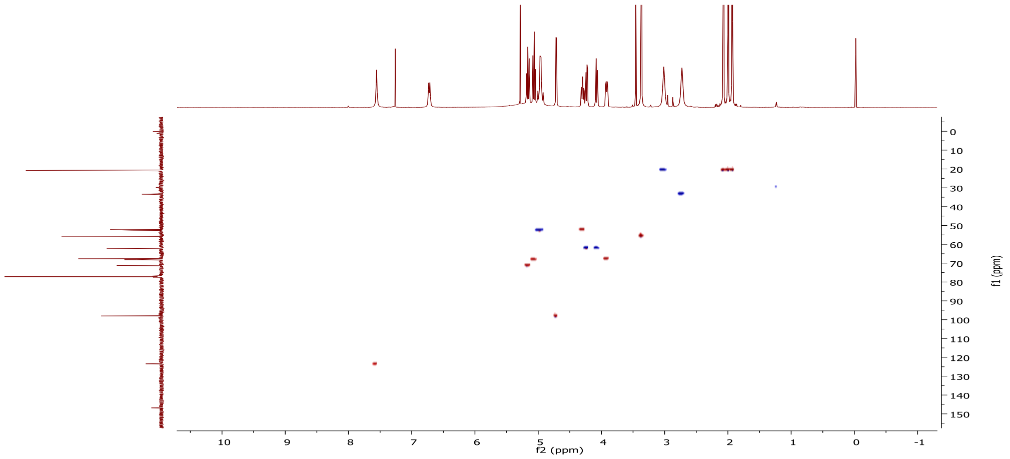

HSQC NMR spectrum of **18** in $CDCl_3$

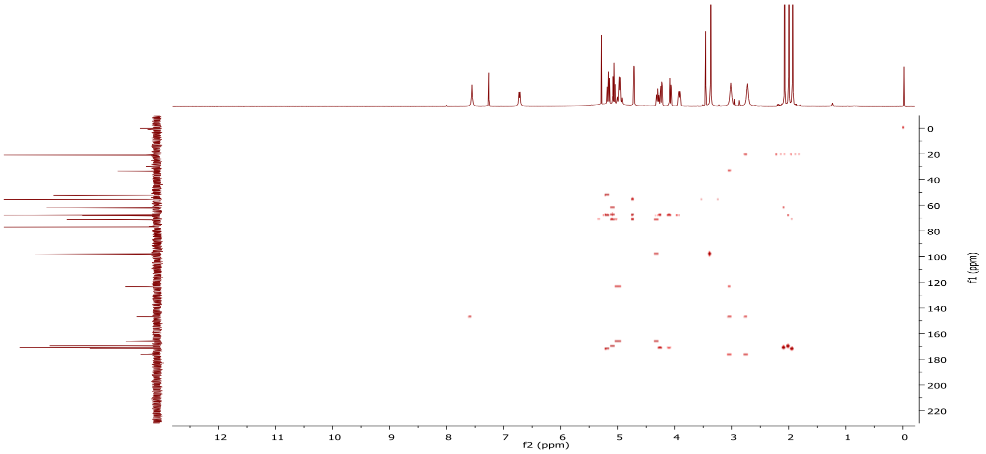

HMBC NMR spectrum of **18** in $CDCl_3$

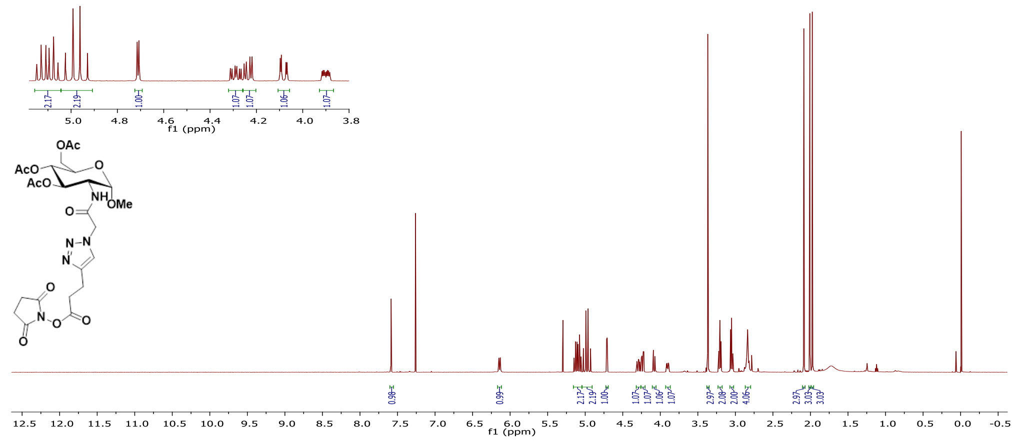

^{1}H NMR spectrum of **19** in $CDCl_3$

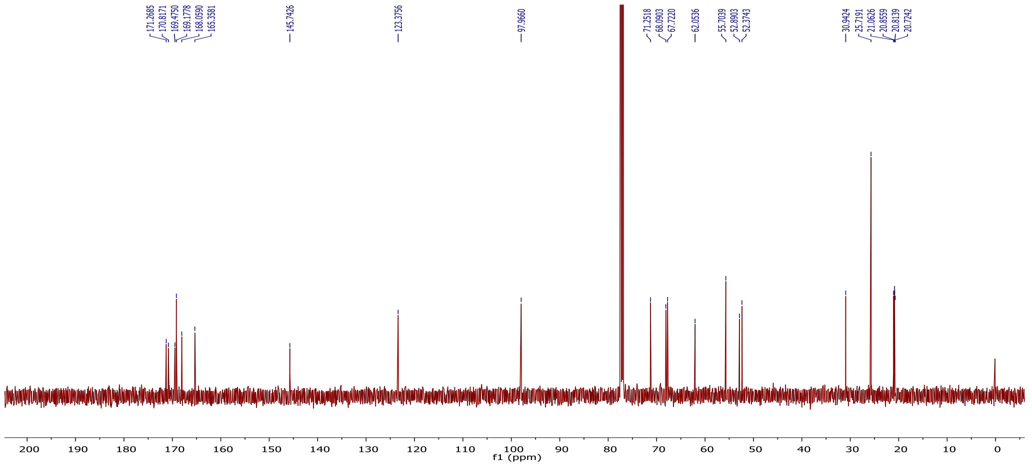

^{13}C NMR spectrum of **19** in $CDCl_3$

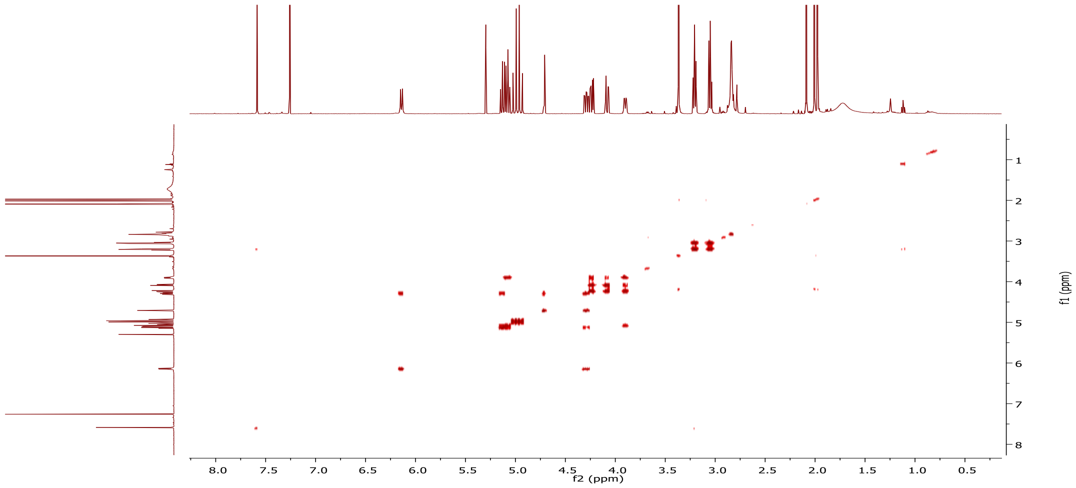

COSY NMR spectrum of **19** in $CDCl_3$

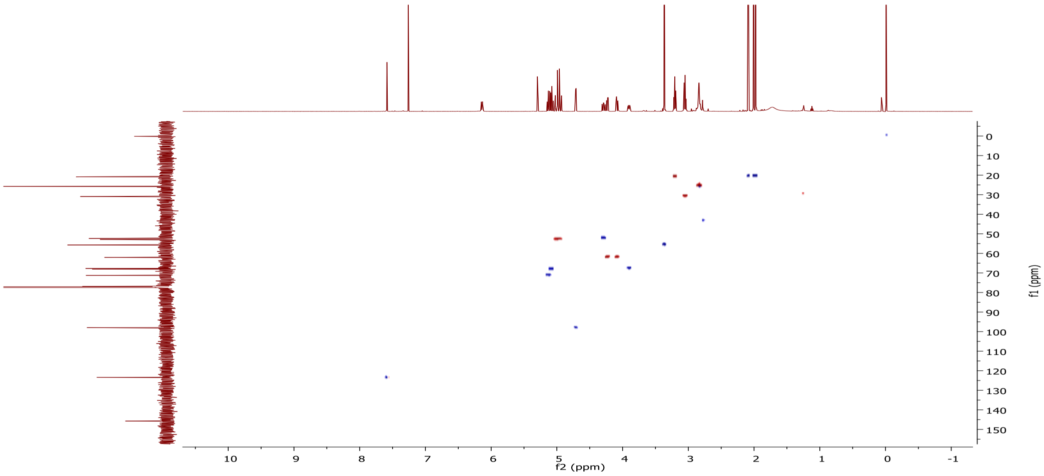

HSQC NMR spectrum of **19** in $CDCl_3$

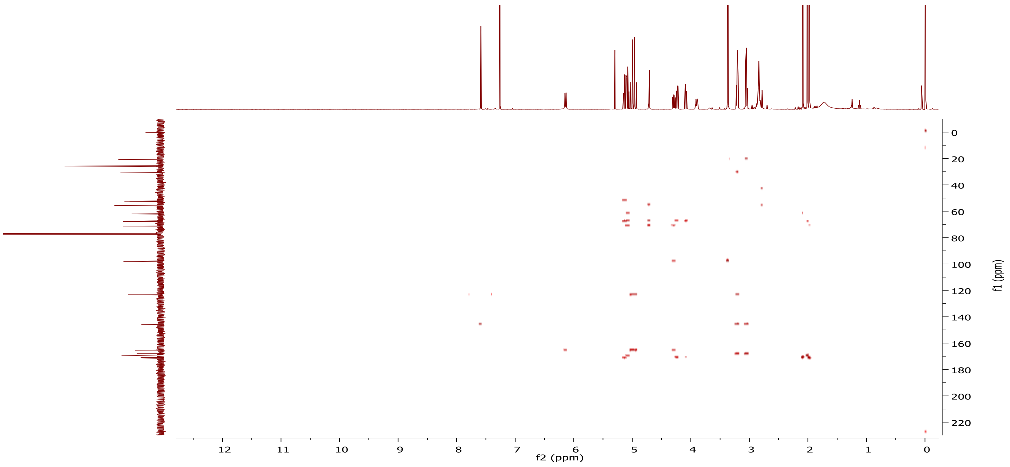

HMBC NMR spectrum of **19** in $CDCl_3$

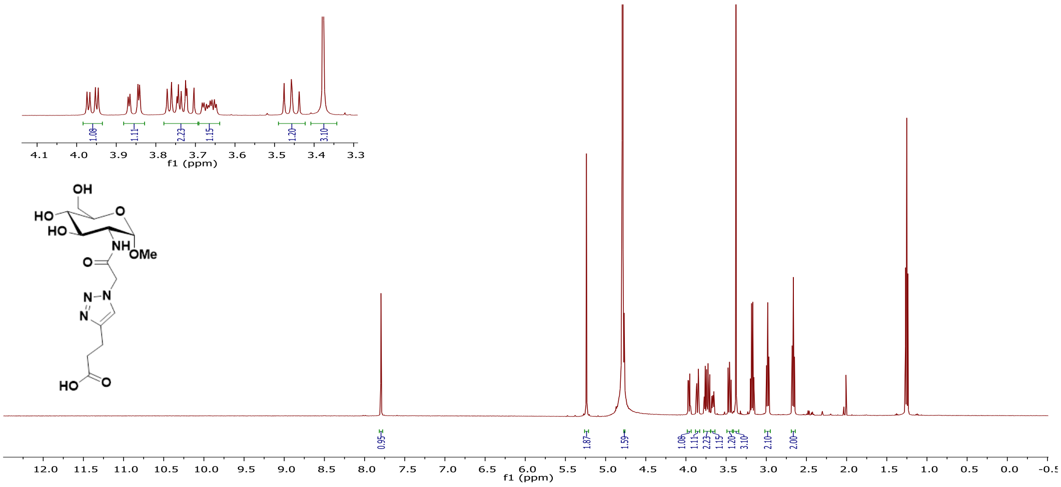

^{1}H NMR spectrum of **20** in DMSO-d_6

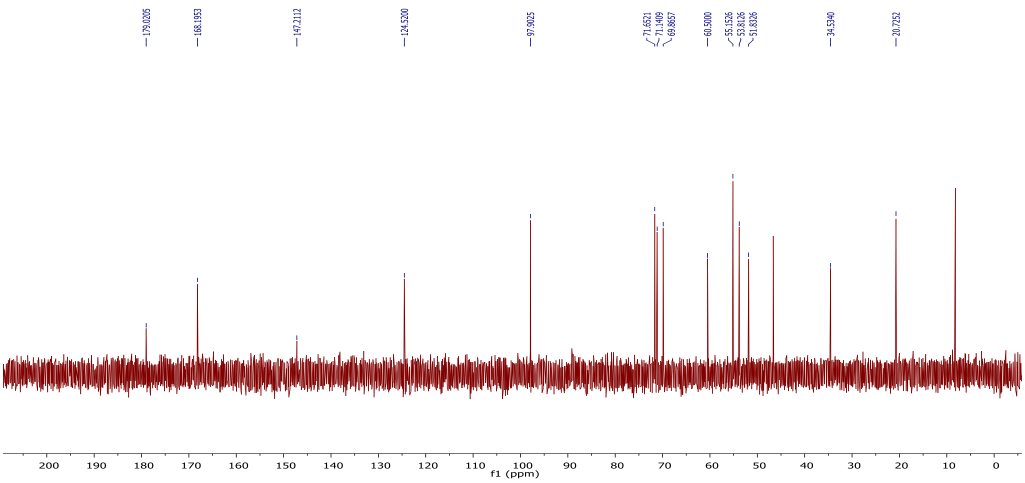

^{13}C NMR spectrum of **20** in DMSO-d_6

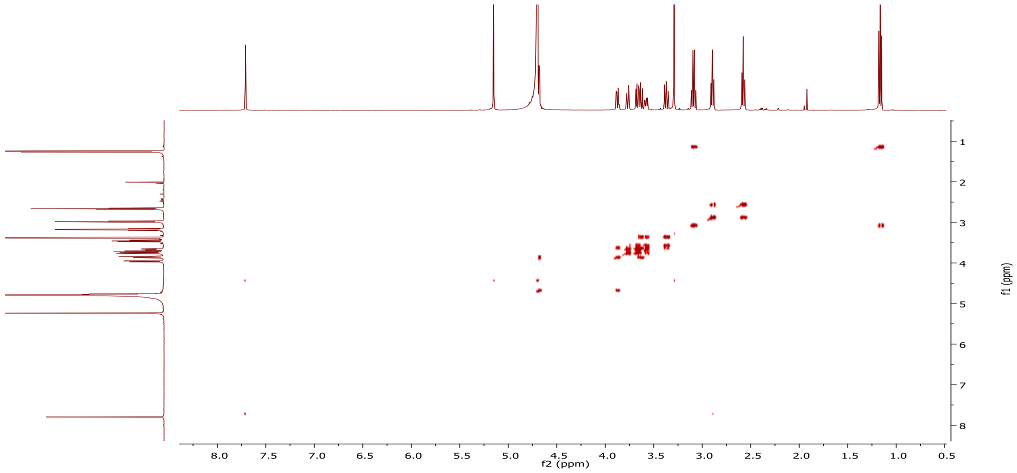

COSY NMR spectrum of **20** in DMSO-d_6

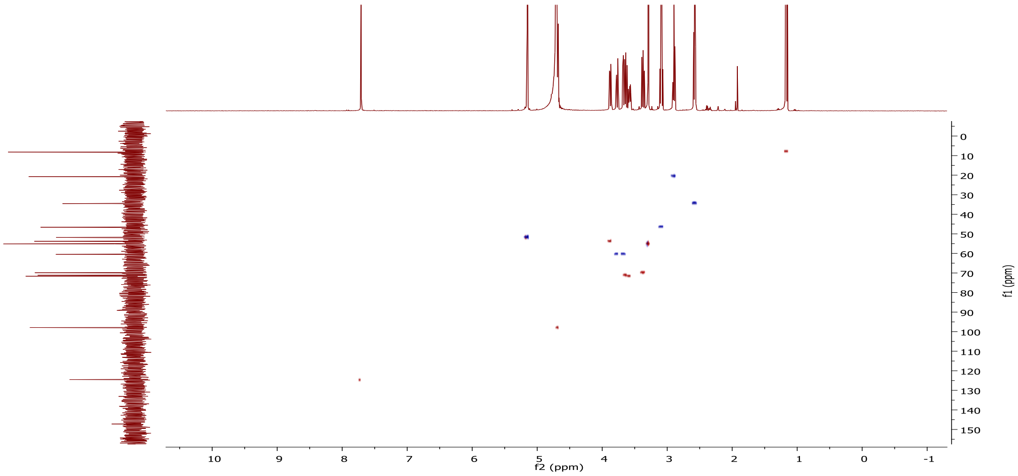

HSQC NMR spectrum of **20** in DMSO-d_6

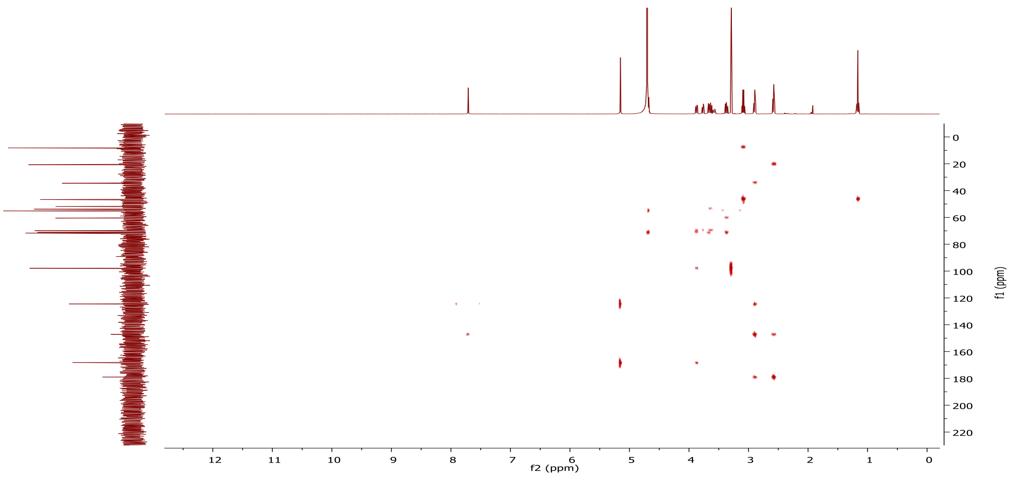

HMBC NMR spectrum of **20** in DMSO-d_6

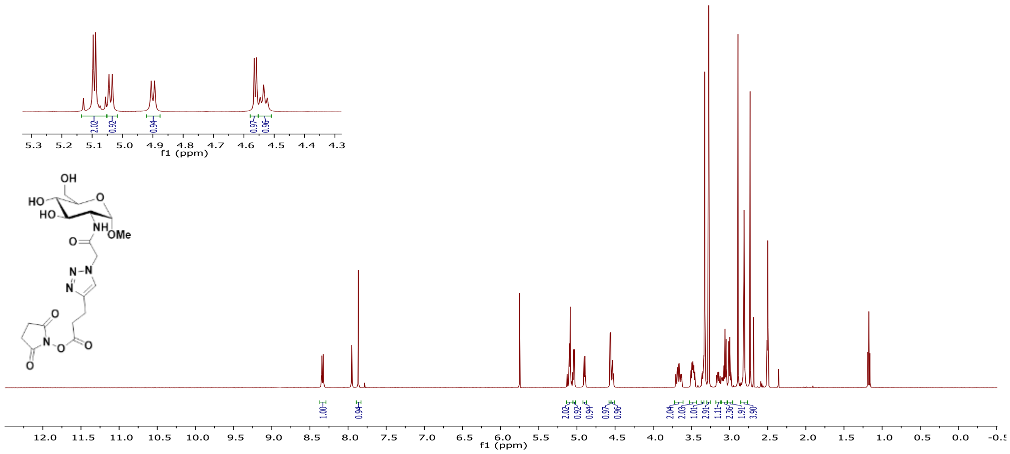

^{1}H NMR spectrum of **21** in DMSO-d_6

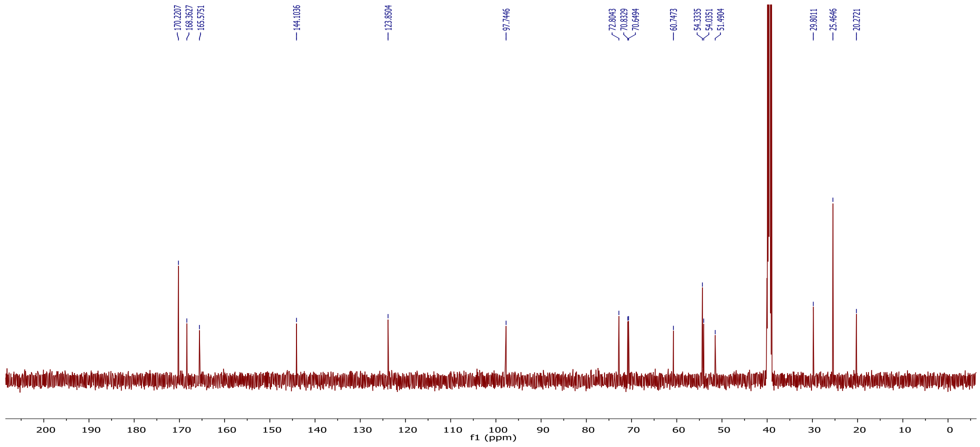

^{13}C NMR spectrum of **21** in DMSO-d_6

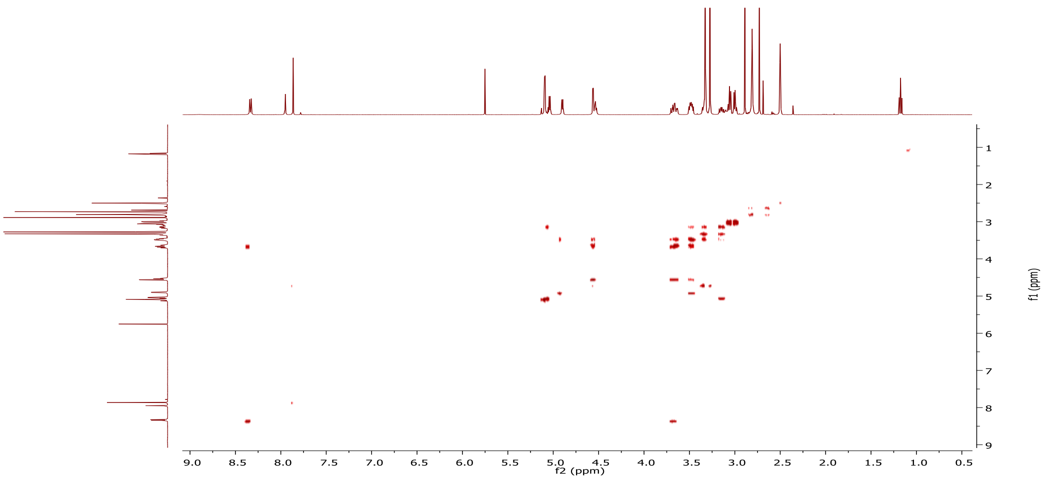

COSY NMR spectrum of **21** in DMSO-d_6

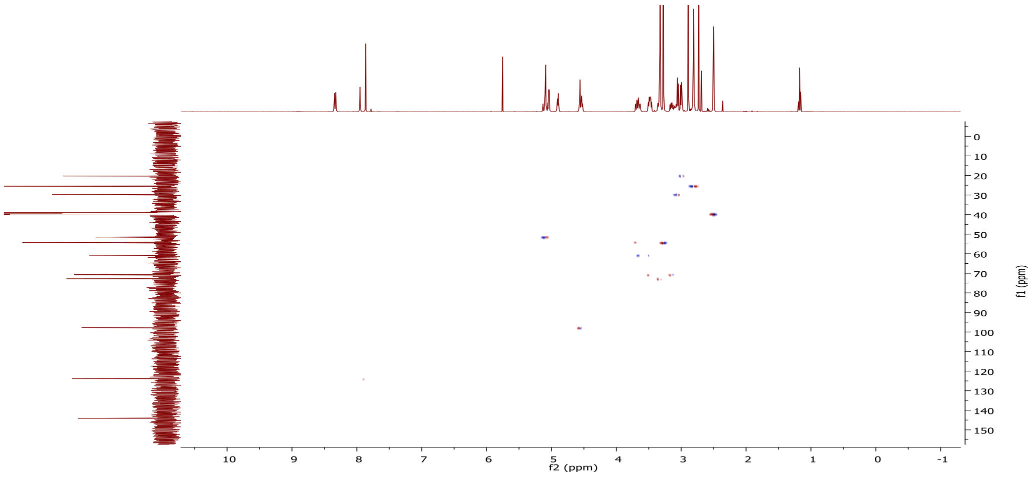

HSQC NMR spectrum of **21** in DMSO-d_6

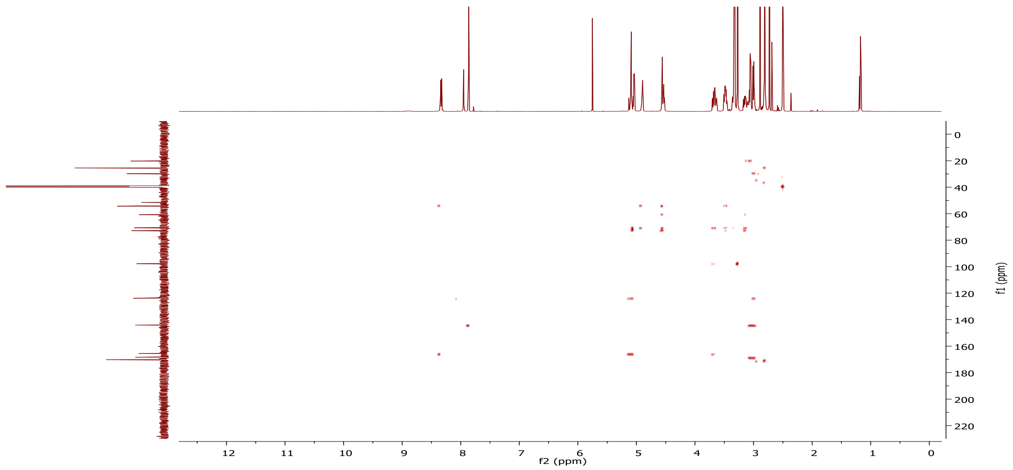

HMBC NMR spectrum of **21** in DMSO-d_6

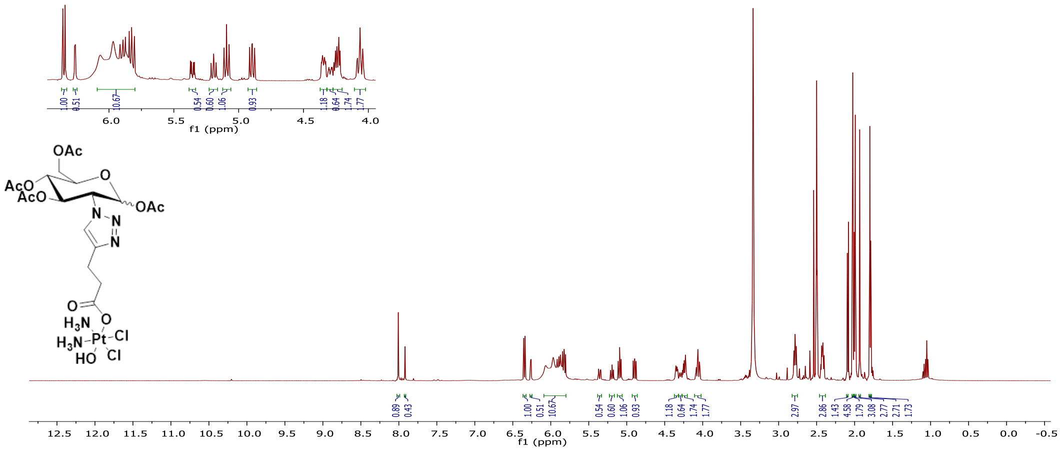

^{1}H NMR spectrum of **complex 1** in DMSO-d_6

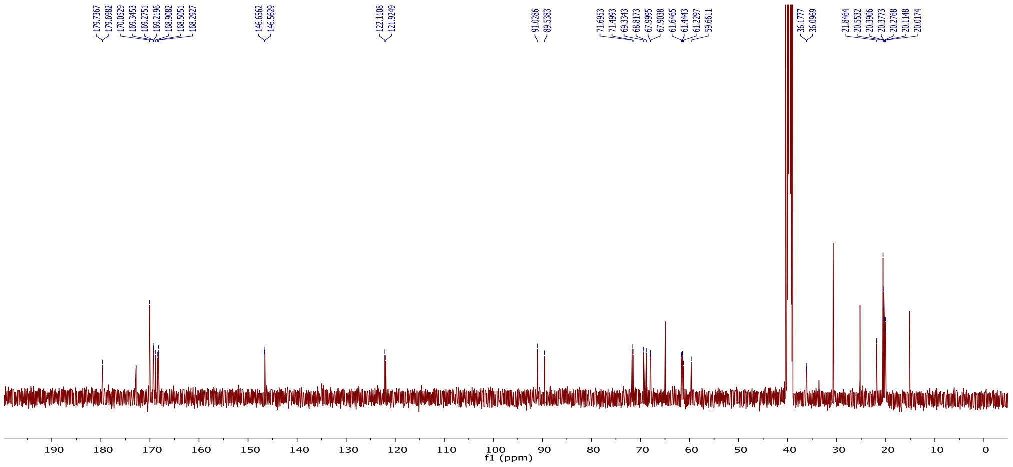

^{13}C NMR spectrum of **complex 1** in DMSO-d_6

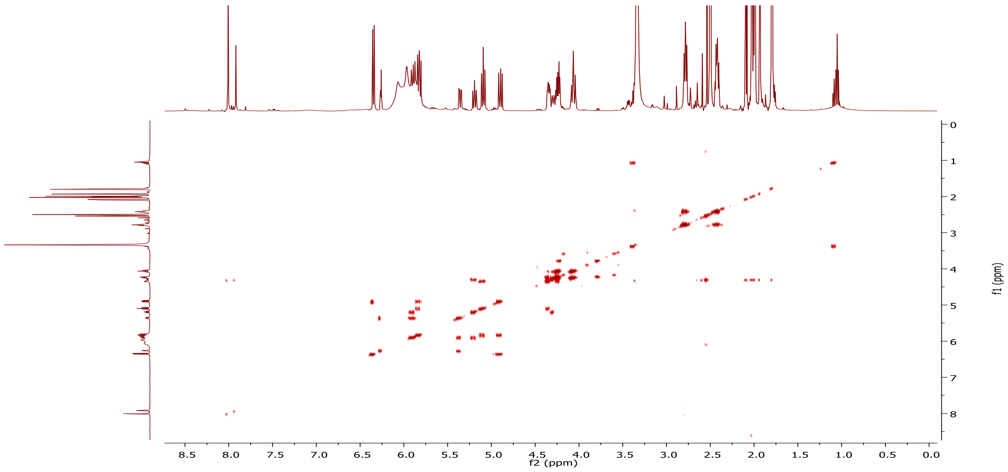

COSY NMR spectrum of **complex 1** in DMSO-d_6

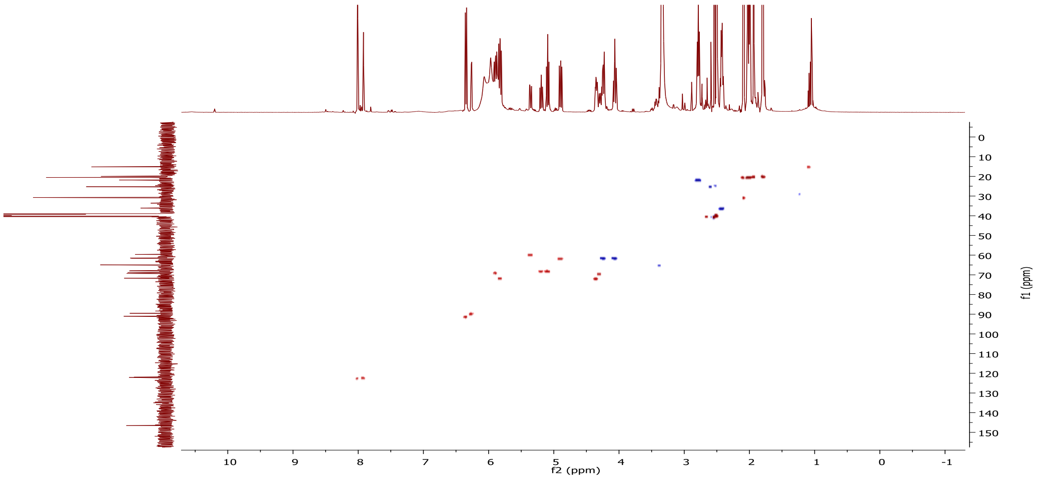

HSQC NMR spectrum of **complex 1** in DMSO-d_6

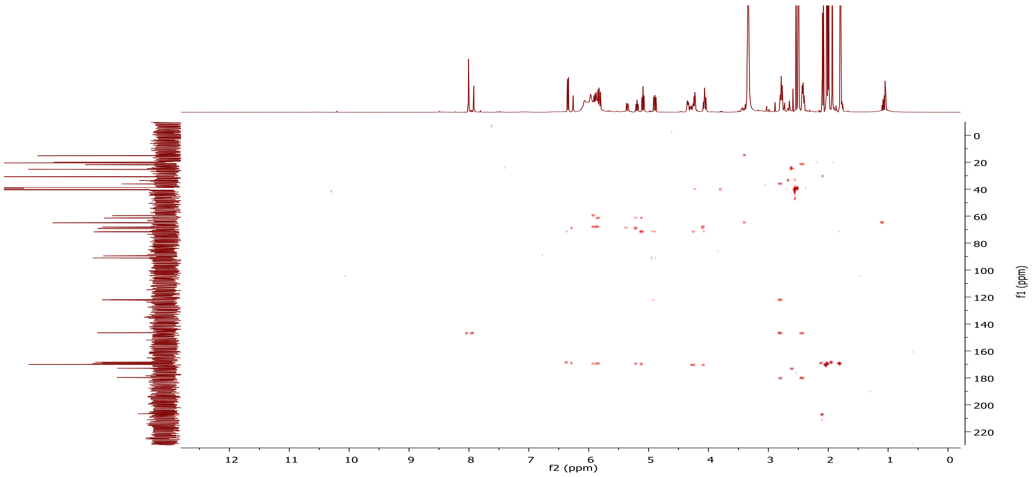

HMBC NMR spectrum of **complex 1** in DMSO-d_6

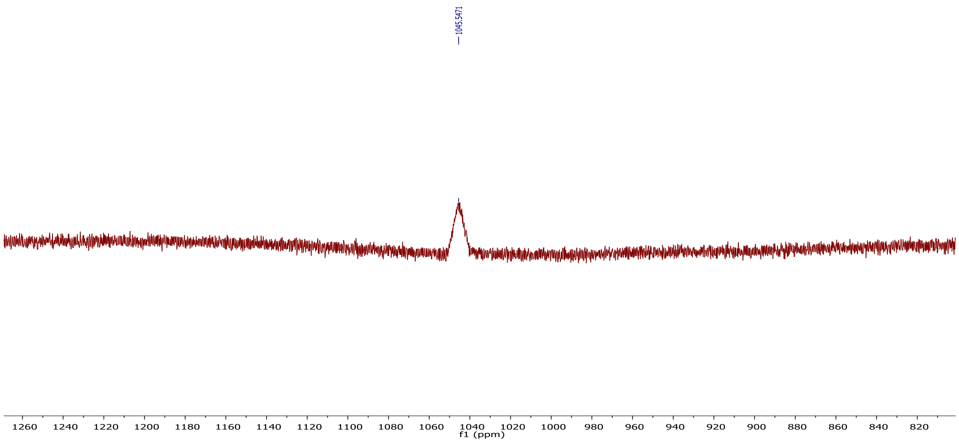

^{195}Pt NMR spectrum of **complex 1** in DMSO-d_6

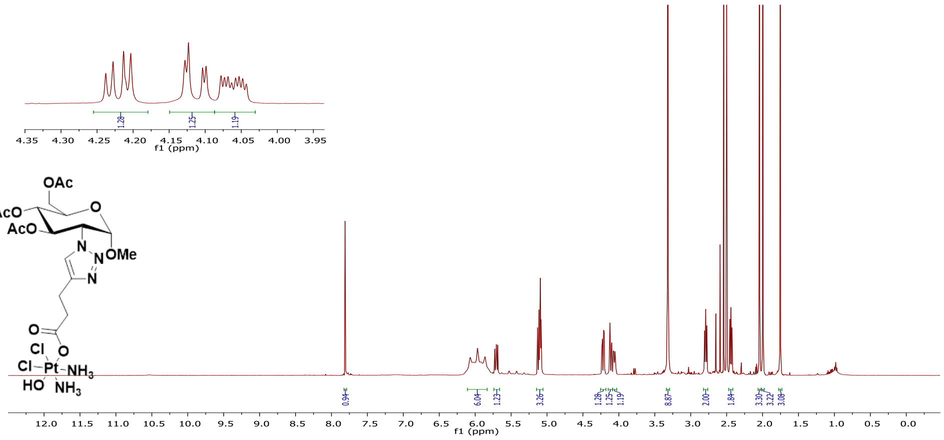

^{1}H NMR spectrum of **complex 2** in DMSO-d$_6$

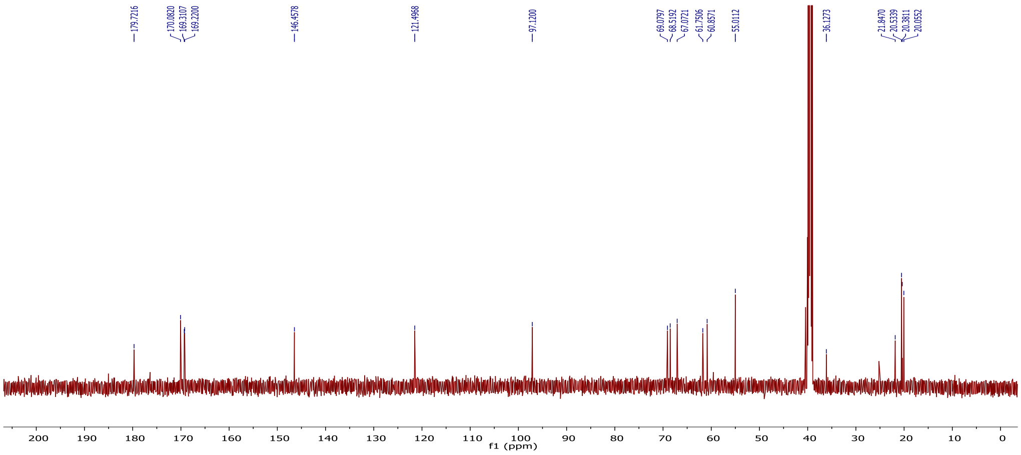

^{13}C NMR spectrum of **complex 2** in DMSO-d$_6$

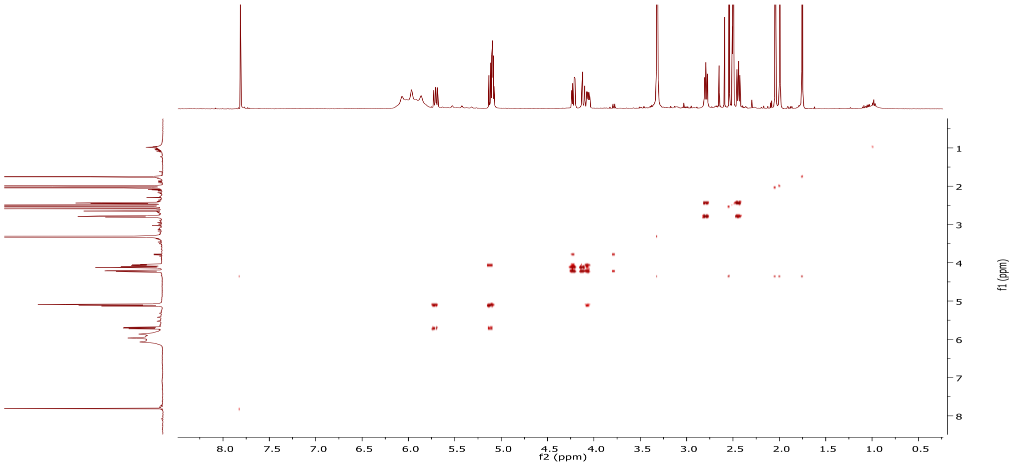

COSY NMR spectrum of **complex 2** in DMSO-d$_6$

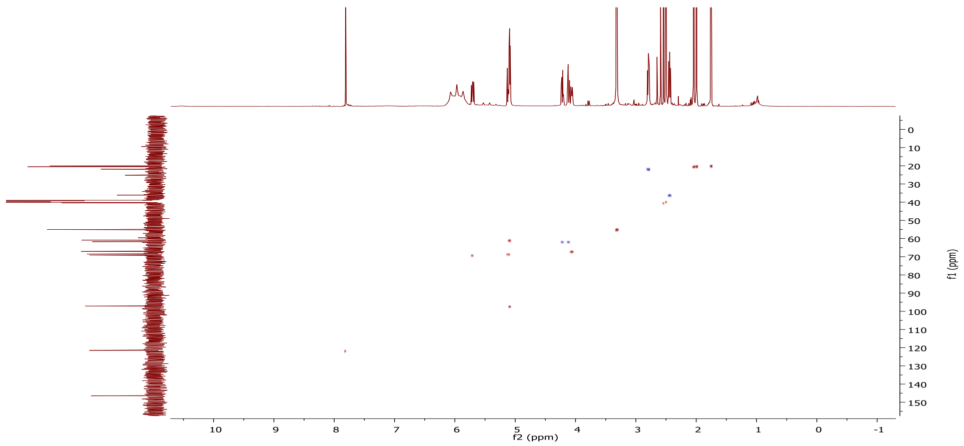

HSQC NMR spectrum of **complex 2** in DMSO-d_6

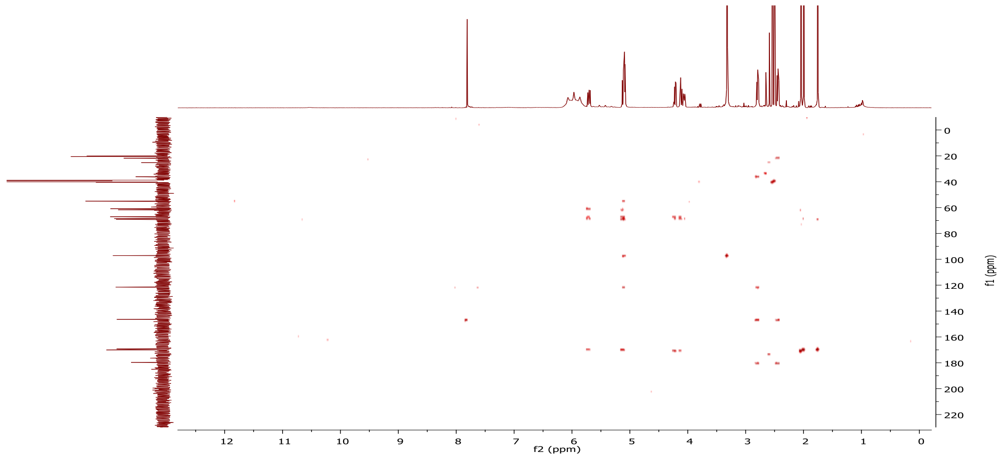

HMBC NMR spectrum of **complex 2** in DMSO-d_6

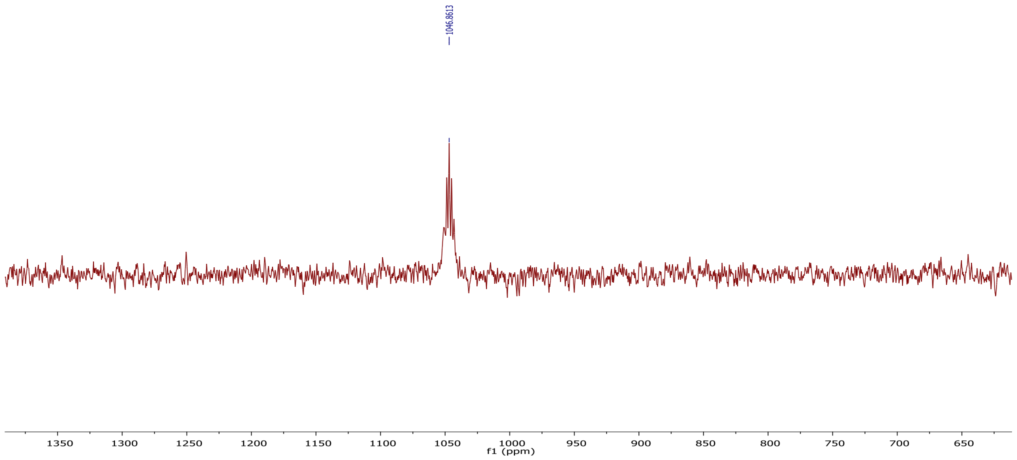

^{195}Pt NMR spectrum of **complex 2** in DMSO-d_6

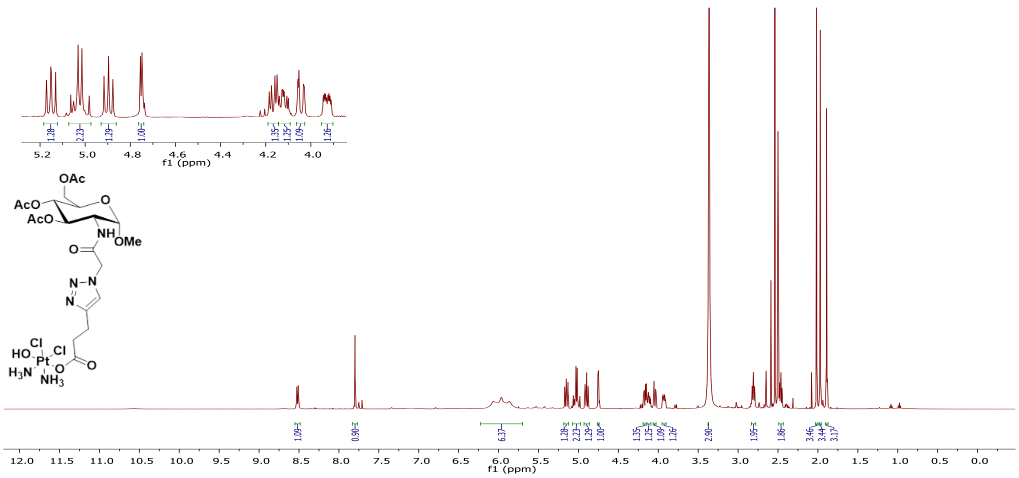

^{1}H NMR spectrum of **complex 3** in DMSO-d_6

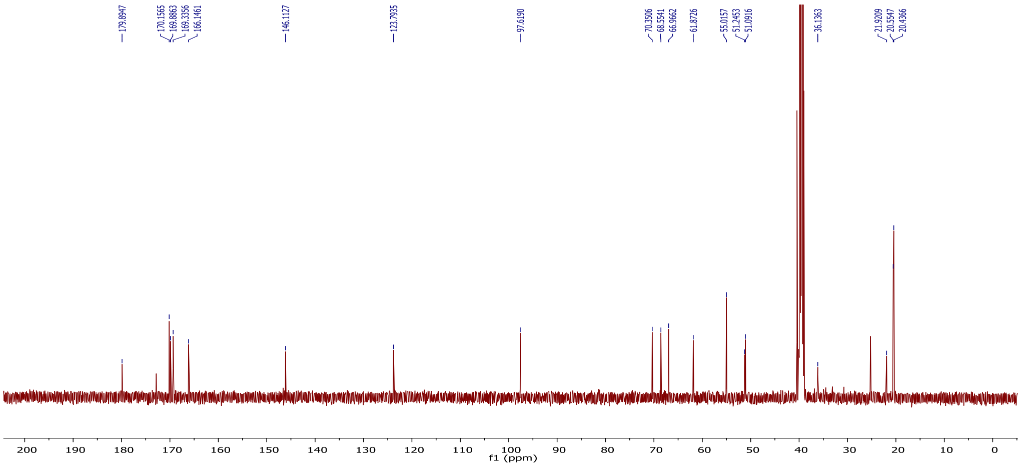

^{13}C NMR spectrum of **complex 3** in DMSO-d_6

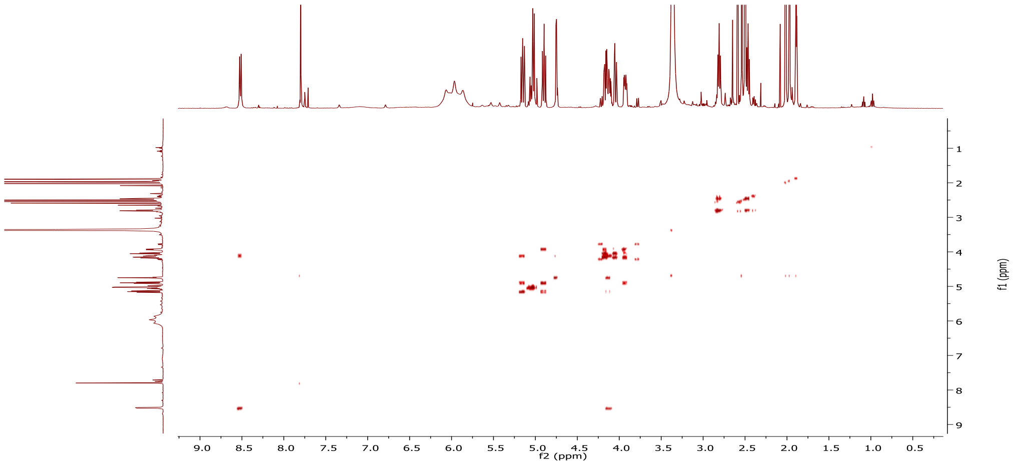

COSY NMR spectrum of **complex 3** in DMSO-d_6

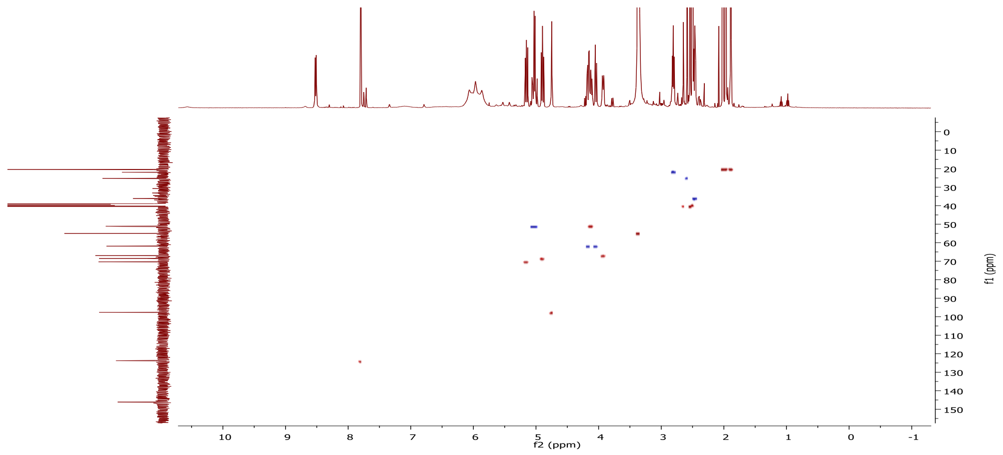

HSQC NMR spectrum of **complex 3** in DMSO-d_6

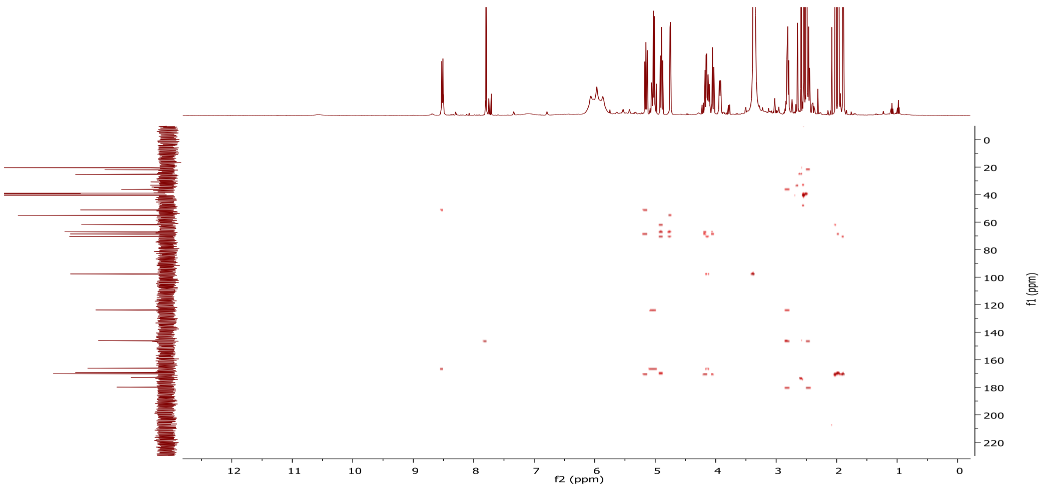

HMBC NMR spectrum of **complex 3** in DMSO-d_6

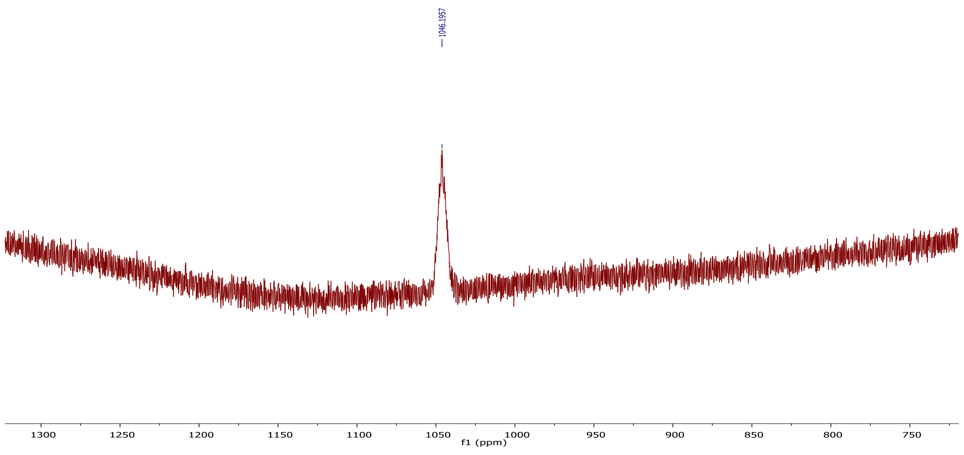

^{195}Pt NMR spectrum of **complex 3** in DMSO-d_6

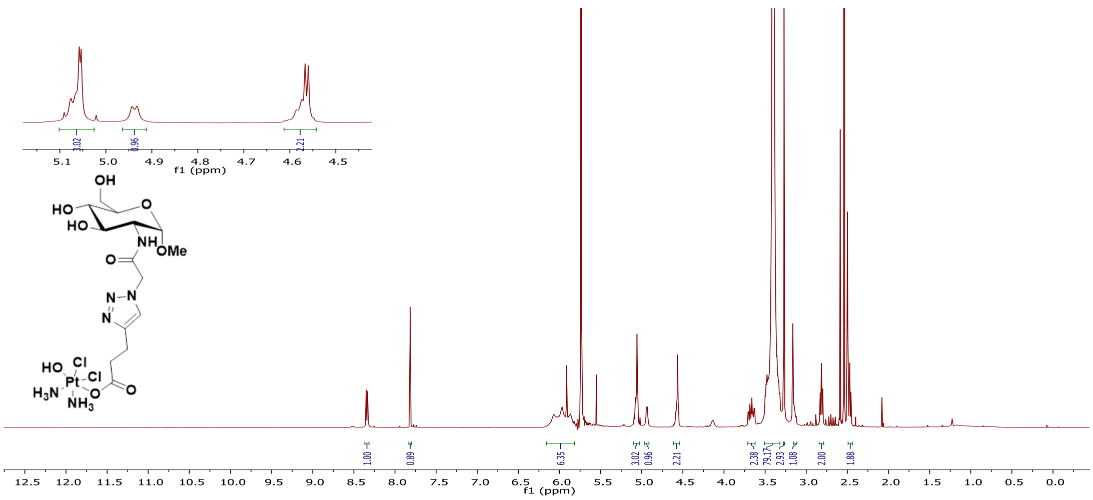

^{1}H NMR spectrum of **complex 4** in DMSO-d_6

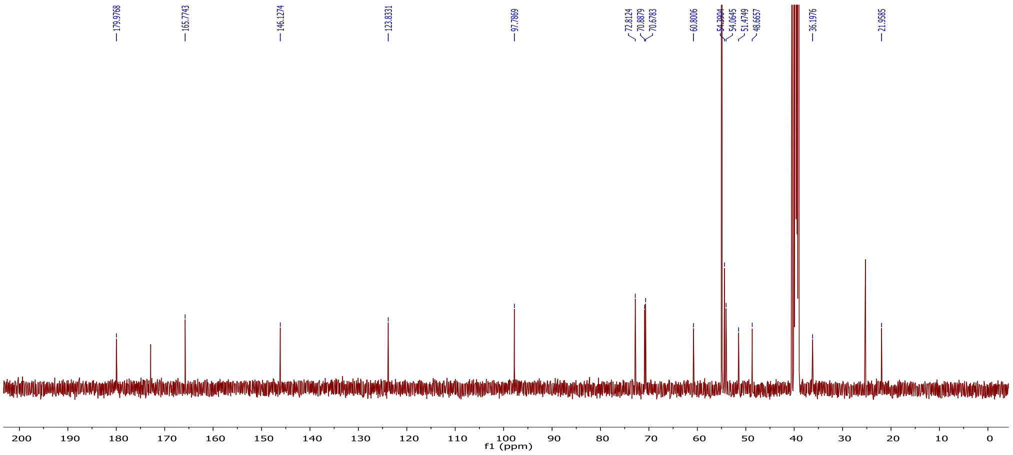

^{13}C NMR spectrum of **complex 4** in DMSO-d_6

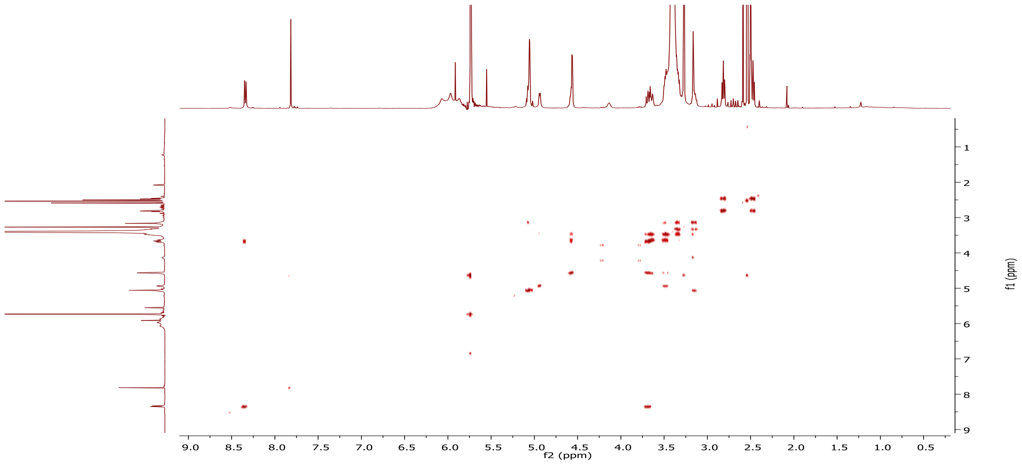

COSY NMR spectrum of **complex 4** in DMSO-d_6

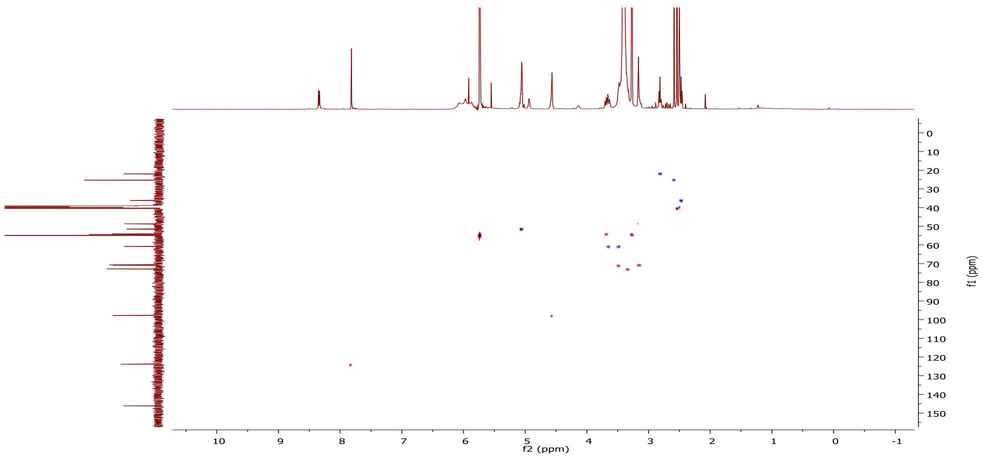

HSQC NMR spectrum of **complex 4** in DMSO-d_6

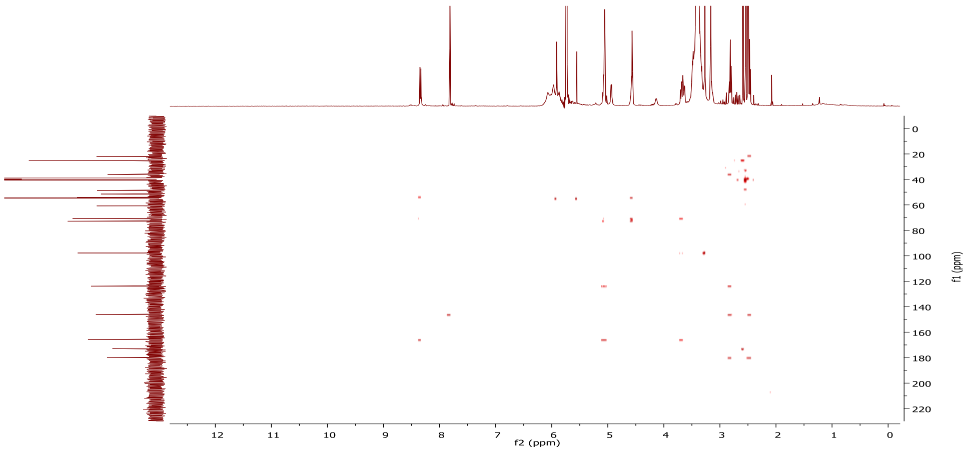

HMBC NMR spectrum of **complex 4** in DMSO-d_6

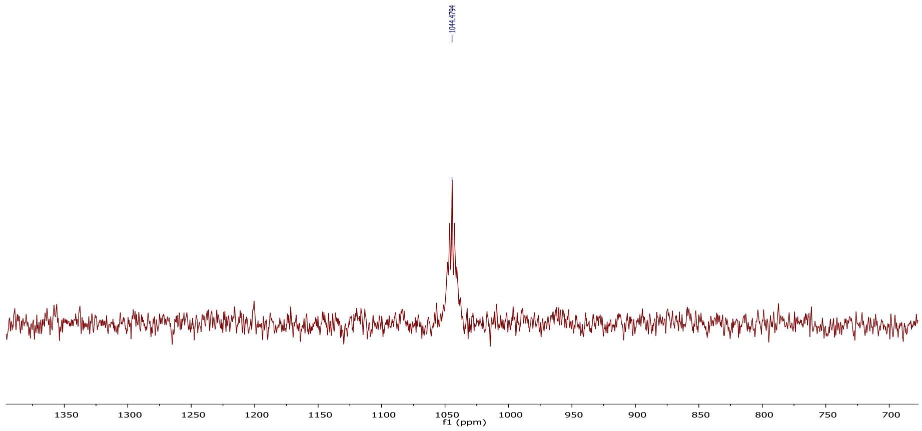

^{195}Pt NMR spectrum of **complex 4** in DMSO-d_6

Mass characterisation

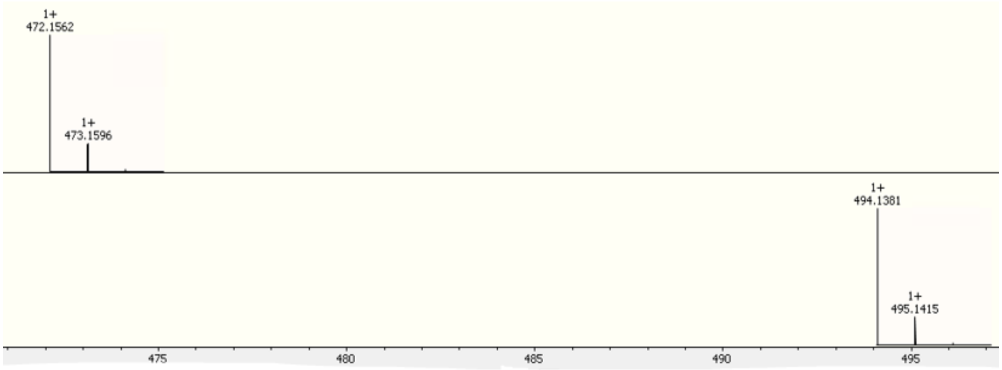

HR-MS spectrum of **7.**

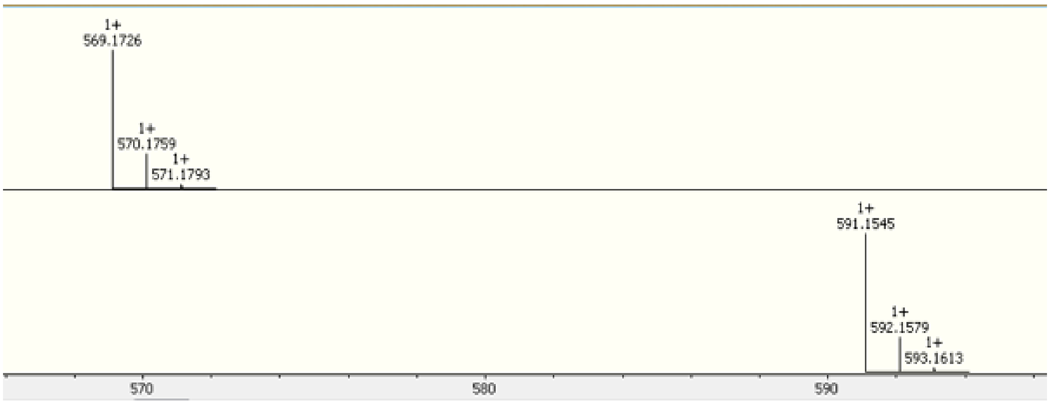

HR-MS spectrum of **8.**

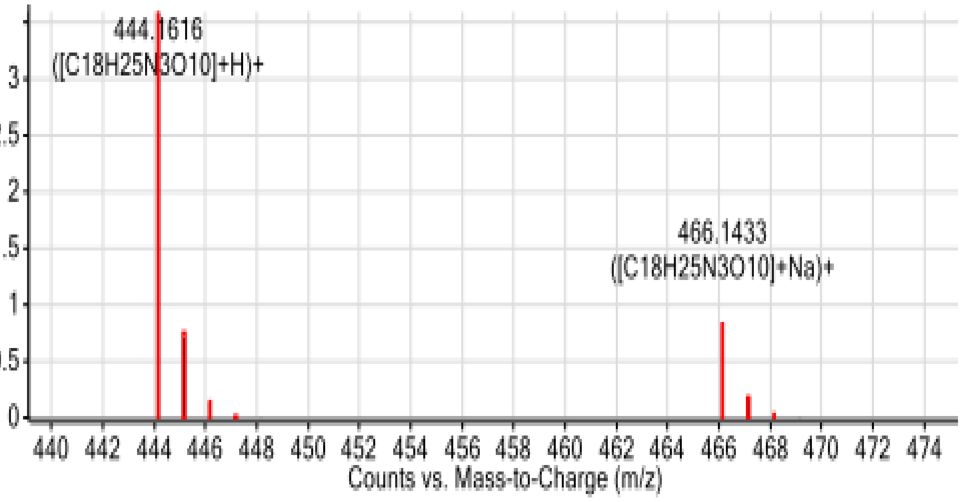

HR-MS spectrum of **14.**

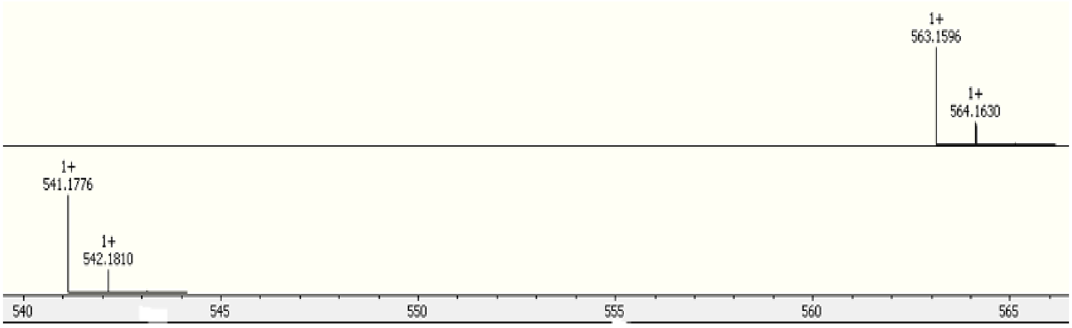

HR-MS spectrum of **15.**

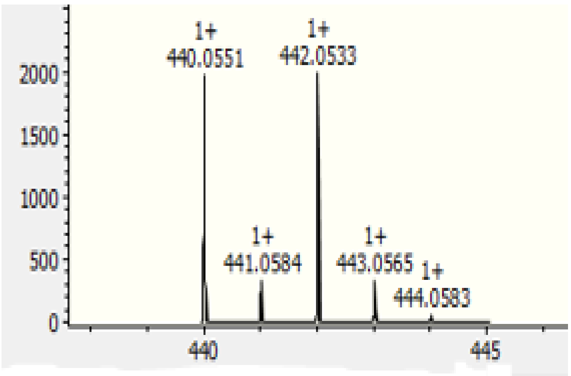

HR-MS spectrum of **16.**

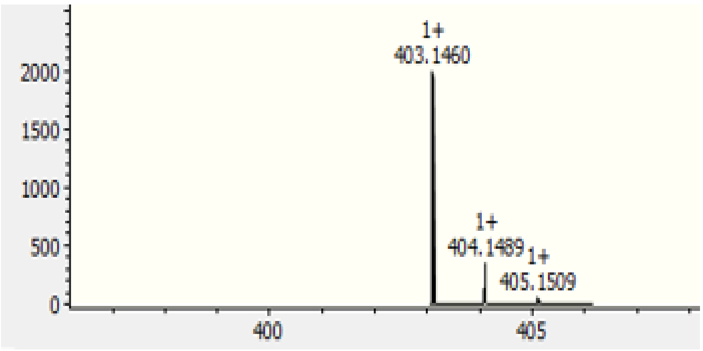

HR-MS spectrum of **17.**

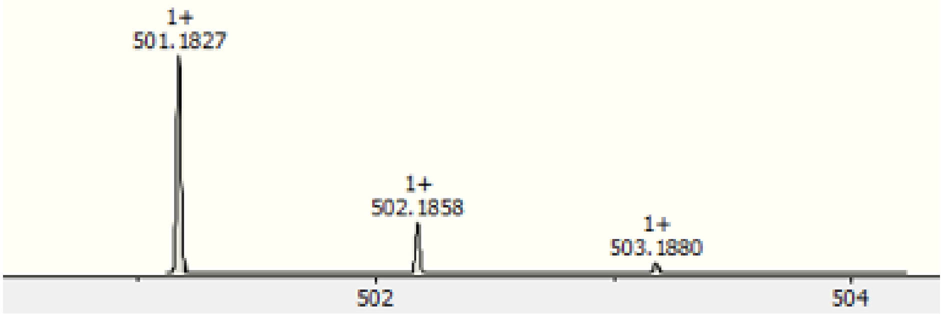

HR_MS spectrum of **18.**

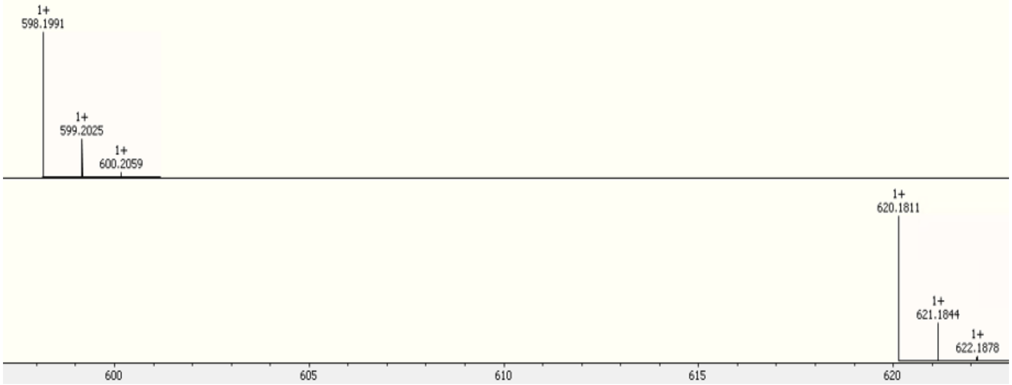

HR-MS spectrum of **19.**

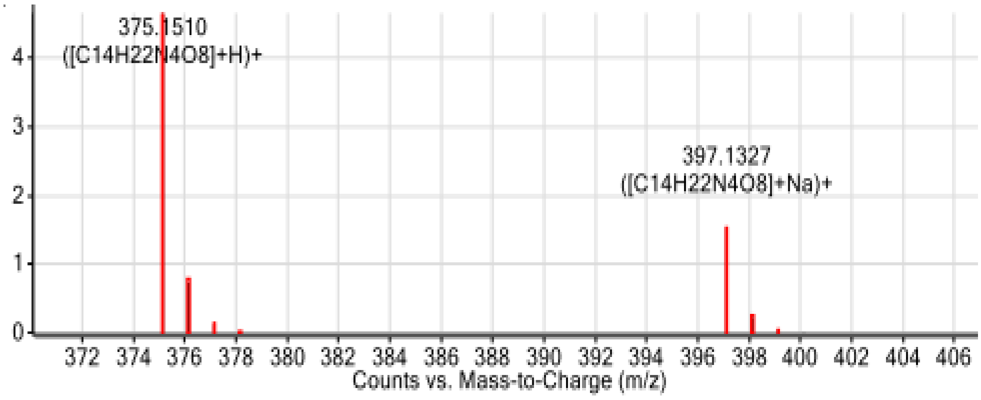

HR-MS spectrum of **20.**

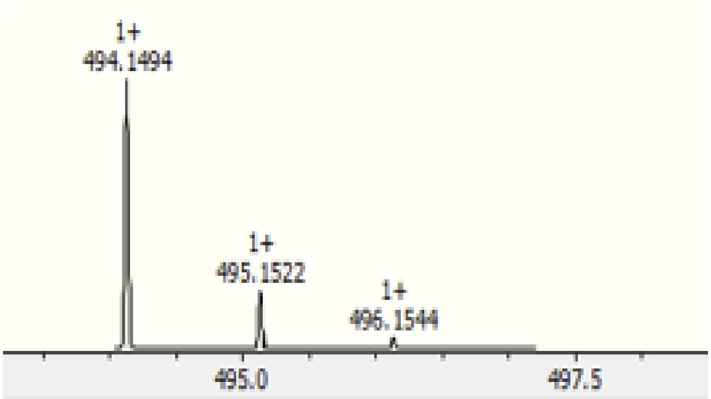

HR-MS spectrum of **21.**

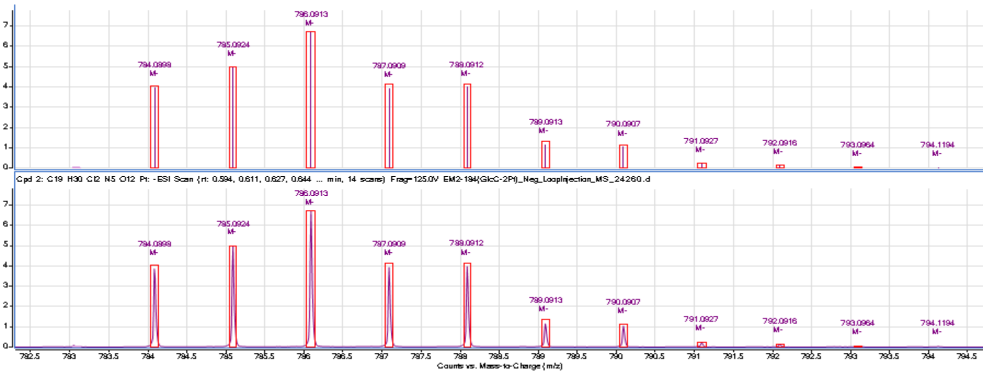

HR-MS spectrum of **complex 1.**

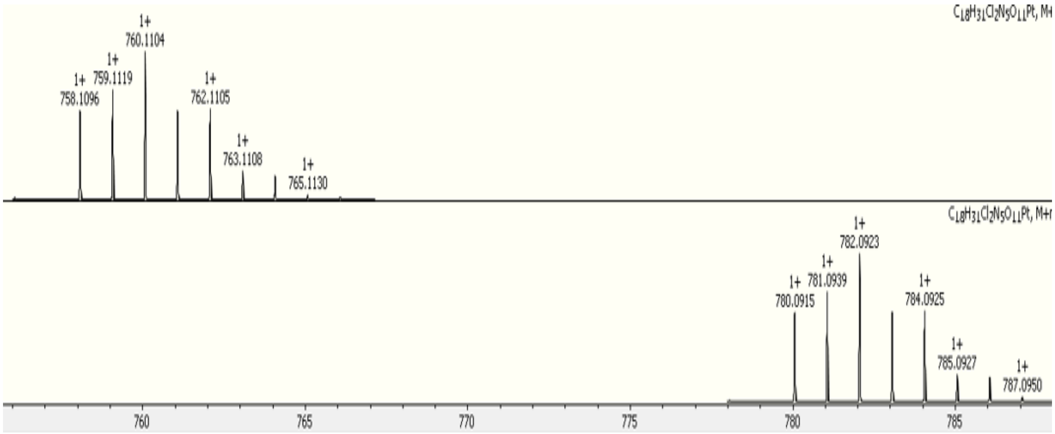

HR-MS spectrum of **complex 2.**

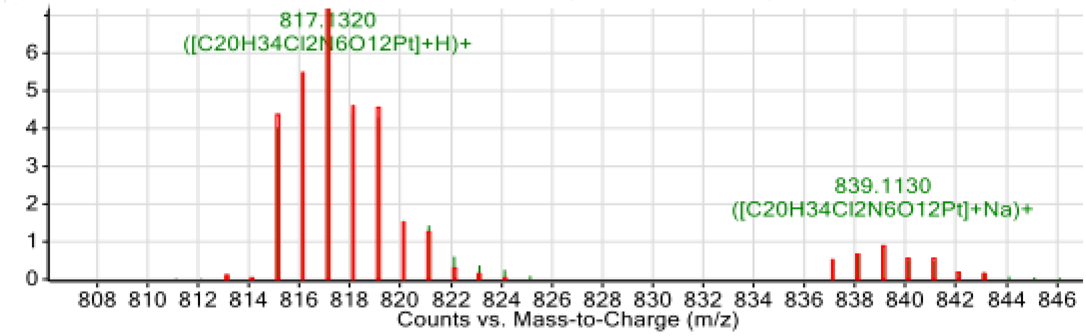

HR-MS spectrum of **complex 3.**

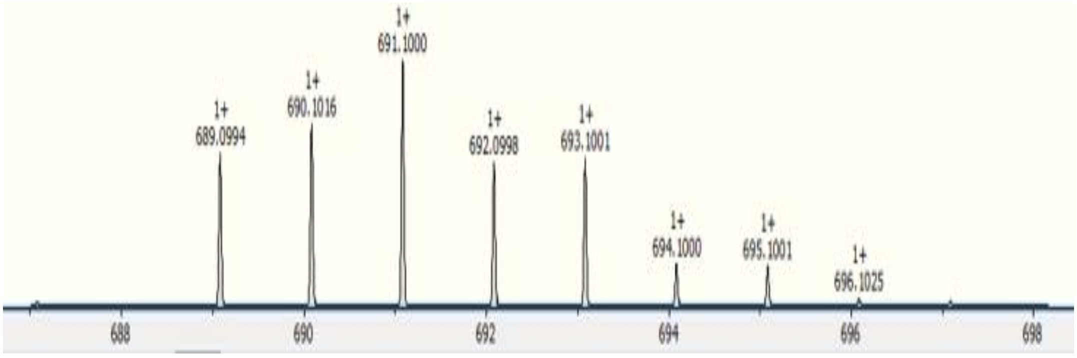

HR-MS spectrum of **complex 4.**

Reduction studies

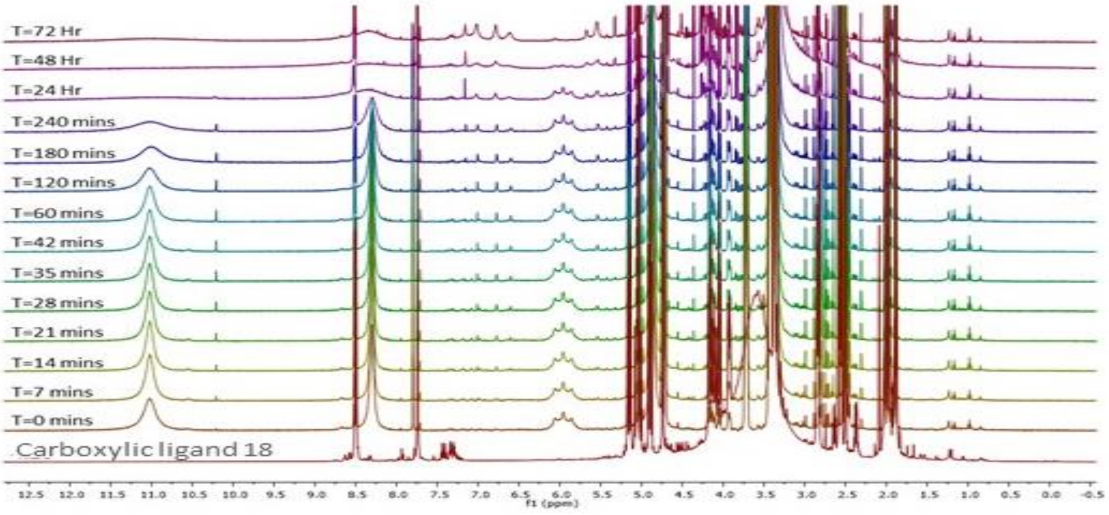

Complex 3: ^{1}H-NMR spectra of the **complex 3** with addition of 10 eq. of ascorbic acid. NMR spectra were collected every 7minutes for 60 minutes and then every hour for 3 hours and finally left to reduce for 3 days.

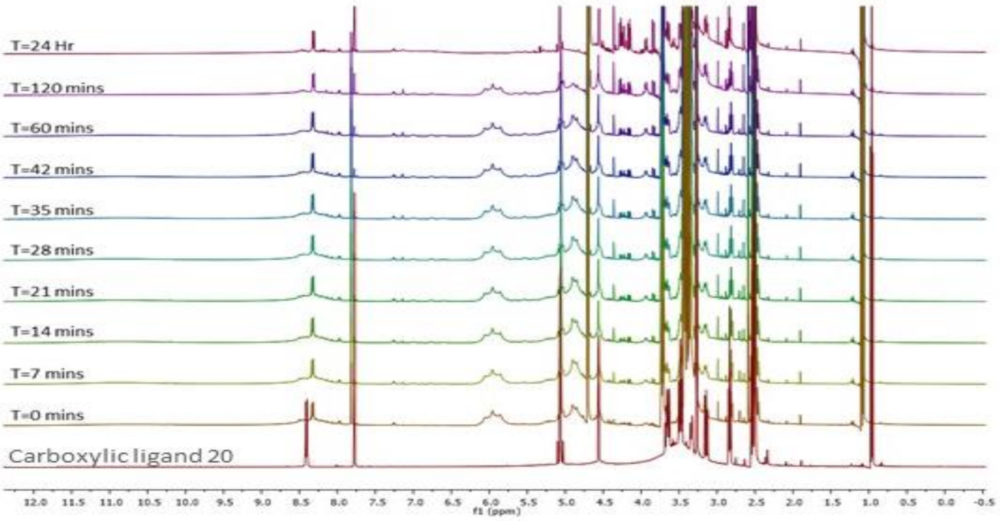

Complex 4: ^{1}H-NMR spectra of the complex **4** with addition of 10 eq. of ascorbic acid. NMR spectra were collected every 7minutes for 60 minutes and then every hour for 2 hours and finally left to reduce for 1 day.
